# Integrated UHPLC-Q-TOF/MS and Liver-on-a-Chip Evaluation of Chemical Composition Changes and Hepatotoxicity Differences in Yaomu Before and After Fermentation

**DOI:** 10.3390/molecules31060994

**Published:** 2026-03-16

**Authors:** Kexin Ma, Lijun An, Guo Feng, Wei Li, Tingting Liu, Jinxin Hou, Ping Wang, Yibao Jin, Bing Wang, Xie-An Yu

**Affiliations:** 1 Department of Chinese Materia Medica, Guizhou University of Traditional Chinese Medicine, Guiyang 550025, China; makexin@stu.gzy.edu.cn (K.M.); lw64@sina.com (W.L.); liutingting@stu.gzy.edu.cn (T.L.); houjinxin@stu.gzy.edu.cn (J.H.); 2 Guizhou Inheritance Base of Traditional Chinese Medicine Processing Technology, Guiyang 550025, China; 3 NMPA Center for Innovation and Research in Regulatory Science, Shenzhen Institute for Drug Control, Shenzhen 518057, China; anlijunyxa@126.com (L.A.); wangping662@sina.com (P.W.); jinyibao2006@126.com (Y.J.)

**Keywords:** Yaomu, fermentation, toxicity, multivariate statistical analysis, quantification, liver on-a-chip

## Abstract

Background: Huafeng Dan (HFD) is a traditional famous medicine from Guizhou Province, commonly used for the treatment of stroke-induced hemiplegia and epilepsy. Yaomu is a key component and serves as the sovereign herb in the formula. Most of the components of Yaomu are toxic Chinese herbal medicines. Traditional fermentation processing methods are required to reduce its toxicity. Purpose: Current studies have not yet systematically analyzed the chemical constituents before and after fermentation. Meanwhile, there is a lack of safety evaluation before and after the fermentation of Yaomu, which can provide a basis for safe clinical medication. Method: Chemical constituents of Yaomu before and after processing were analyzed using UHPLC-Q/TOF-MS to compare compositional changes induced by fermentation. To further screen potential toxic components, representative compounds were selected from these differential compounds based on statistical indicators (such as VIP value), low cost and easy availability, as well as criteria from the literature, and the content changes before and after fermentation were investigated. In vitro toxicity was evaluated using a microfluidic liver organ-on-a-chip model to assess the toxic effects of Yaomu extracts before and after fermentation. Results: Studies have shown that in both positive and negative ionization modes, a total of 361 compounds were annotated in unfermented Yaomu. After fermentation, a total of 350 compounds were annotated. Multivariate statistical analysis revealed significant differences in the chemical composition of Yaomu before and after fermentation. Quantitative analysis demonstrated that the levels of diester-type diterpenoid alkaloids were significantly reduced after fermentation, accompanied by concurrent decreases in lysophosphatidylcholine (LPC) species, compared with unfermented Yaomu. In contrast, the concentrations of amino alcohol-type diterpenoid alkaloids were significantly increased. The microfluidic liver organ-on-a-chip results demonstrated that the post-fermentation extract caused significantly attenuated impairment of hepatocellular function and viability. The in vitro toxicity findings showed good concordance.

## 1. Introduction

HFD is a traditionally famous medicine of Guizhou Province, composed of 14 Chinese medicinal materials—including Perilla Leaf, Bombyx batryticatus, Scorpion, and Atractylodes, among others. It is listed in the National Compilation of Standards for Traditional Chinese Patent Medicines–Internal Medicine Volume, and was recognized in 1951 as one of the four famous traditional medicines protected by the State Council of China. Clinically, Huafeng Dan is commonly prescribed for the treatment of phlegm-wind obstruction syndrome, hemiplegia caused by stroke, and epilepsy. Yaomu, serving as the sovereign drug in the formula, mainly consists of *Typhonii Rhizoma* (Baifuzi), *Pinelliae Rhizoma* (Banxia), *Arisaematis Rhizoma* (Tiannanxing), *Aconiti Radix* (Chuanwu), and *Curcumae Radix* (Yujin). These herbs are fermented together with bovine bile and *Massa Medicata Fermentata* (Shenqu) to produce the final Yaomu preparation. The previous study conducted by our research group investigated the synergistic mechanism of fermented Yaomu in enhancing the therapeutic efficacy of HFD against ischemic stroke. Metabolomic analysis identified five key metabolic pathways involved in the therapeutic effect of HFD on ischemic stroke and revealed that fermentation of Yaomu primarily enhances the efficacy of HFD through modulation of the tryptophan metabolism pathway [1]. Yaomu is not only the key component responsible for the primary therapeutic effects of Huafeng Dan, but its complex formulation and unique fermentation process also embody the wisdom of traditional Chinese medicine in toxicity reduction and efficacy enhancement. It serves as an essential material basis for the treatment of central nervous system diseases by Huafeng Dan. Therefore, it is of great significance to conduct in-depth research on the safety of Yaomu.

The primary objective of traditional Chinese medicine (TCM) processing, or *Paozhi*, is to reduce or eliminate the toxicity and adverse effects of medicinal substances, as well as to alter their therapeutic properties. For centuries, this practice has been employed to mitigate the toxicity of alkaloid-rich herbs [2]. Fermentation is a traditional processing method in Chinese medicine. China has a long history of fermentation technology spanning thousands of years. Building upon this foundation, extensive research has gradually revealed the potential of microbial metabolism to modify medicinal properties, leading to the systematic application of fermentation in TCM processing [3]. Microbial fermentation can alter the properties and efficacy of herbal medicines, reduce their toxicity and adverse effects, and enhance the therapeutic efficacy of TCM decoctions, thereby improving their suitability for clinical applications. Under appropriate conditions, TCMs undergo fermentation through the metabolic activities of microorganisms [4,5]. Yaomu contains a complex mixture of components, many of which are highly irritating. Therefore, Yaomu is also processed through fermentation.

The unique fermentation process of Yaomu involves thoroughly mixing the raw medicinal materials with bovine bile, placing the mixture in a sealed container, and fermenting it under controlled conditions of 20–40 °C and 50–80% relative humidity for 30–35 days. After fermentation, the material is removed and dried to obtain the final product. During the fermentation of Huafeng Dan Yaomu, Cao et al. (2020) [6] employed high-throughput sequencing to characterize the dynamic succession of microbial communities. At the initial stage of fermentation (day 0), Bacillus (≈58.38%), Enterobacter (≈16.05%), and Enterococcus (≈4.10%) were identified as the dominant bacterial genera. After 7 days of fermentation, Saccharomycopsis was increased to 90.84%. After 14 days of fermentation, Pichia became the main fungal genera in the fermentation process. Moreover, their correlation analysis revealed that 16 bacterial genera and 7 fungal genera were significantly associated with changes in toxic alkaloids during the fermentation process [6]. However, critical knowledge gaps remain regarding the comprehensive chemical changes induced by fermentation and their associated toxicological profiles, which have yet to be systematically investigated.

UHPLC-QTOF-MS/MS is used for chemical characterization because it combines high chromatographic resolution (UPLC) with high-resolution, accurate-mass full-scan MS capability (Q-TOF), making it especially suited for untargeted metabolomics and comprehensive profiling of complex TCM matrices [7,8]. Hence, the main objective of the present study was to characterize the chemical profiles of Yaomu before and after fermentation using UHPLC-QTOF-MS/MS and to use multivariate statistical analysis to identify differential compounds. Given the limited reports on the quantitative changes of potential toxic components in Yaomu before and after fermentation, this study establishes an LC-MS/MS method for the simultaneous determination of these candidate toxic candidates, to provide quantitative evidence for the detoxification effect of fermentation. Moreover, recent advances in microfluidic Lab-on-a-chip (LOC) technologies have provided powerful platforms for preclinical toxicity evaluation by more closely resembling human physiological and microenvironmental conditions than traditional static 2D cultures. LOC devices integrate microfluidic flow and 3D tissue architecture to simulate dynamic organ-specific functions such as metabolic activity, shear stress exposure, and nutrient gradients, thereby improving the relevance of toxicity assessments to human biology [9]. In particular, liver-on-a-chip models have demonstrated enhanced maintenance of hepatocellular function and drug metabolism capabilities, enabling the study of drug-induced liver injury and xenobiotic metabolism in vitro with higher physiological fidelity [10,11]. The continuous perfusion and microenvironmental control of these systems facilitate nutrient supply and waste removal, promote expression of key metabolic enzymes, and allow integration of drug biotransformation and toxic responses in a single platform [11,12]. Human organ-on-a-chip systems—such as liver chips—portray key aspects of human organ physiology, including metabolic function, microarchitecture, and cellular heterogeneity, under controlled microfluidic environments, thereby providing human-relevant mechanistic insights into drug metabolism and toxicity [13,14].

Herein, this study will unveil the toxicity reduction effect of the fermentation of Yaomu from two dimensions: component changes and toxicity mechanisms. Comprehensive analysis methods such as UHPLC-QTOF-MS/MS, multivariate statistical data processing methods, and UHPLC -QQQ– MS/MS quantification were employed to study the changes in chemical components and the transformation mechanism. The components of Yaomu are highly complex, and after oral administration most compounds undergo first-pass hepatic metabolism, making the liver a primary target for their toxic effects; therefore, systematic evaluation of hepatotoxicity is critically important for safe clinical use. A liver microfluidic chip was used to explore the different impacts on liver toxicity before and after processing. The possible mechanism of Yaomu’s liver toxicity was investigated from the aspect of molecular biology and the overall toxicity impacts before and after Yaomu fermentation were explored. The comprehensive results provide strong evidence for the rationality of the processing methods of ethnic medicines, and further provide strong evidence for the safe, effective, and clinically rational application of HFD.

## 2. Results

### 2.1. Compositional Profiling of the Yaomu Before and After Fermentation

Extracted ion chromatograms (EICs) of identified compounds were acquired in both positive and negative ion modes, as illustrated in Figure S1. The results demonstrated superior response signals for Yaomu compounds in positive ion mode, with quasi-molecular ions predominantly observed as [M+H]^+^. The chemical composition information of Aconite (Fuzi) [15,16,17], Pinellia (Banxia) [18,19,20,21,22], Arisaema (Tiannanxing) [23,24,25], Sichuan Aconite (Chuanwu) [26,27,28,29,30,31,32,33,34], Curcuma (Yujin) [35], and bile fermentation products [1,36,37] was collected through searches in databases such as PubChem (https://pubchem.ncbi.nlm.nih.gov, accessed on 10 September 2024), CNKI (https://www.cnki.net, accessed on 12 September 2024), and ChemSpider (https://www.chemspider.com, accessed on 15 September 2024). This information was compared with the retention times, accurate molecular weights, fragment ions, and fragmentation behaviors of the compounds for identification and verification.

**Figure 1:**
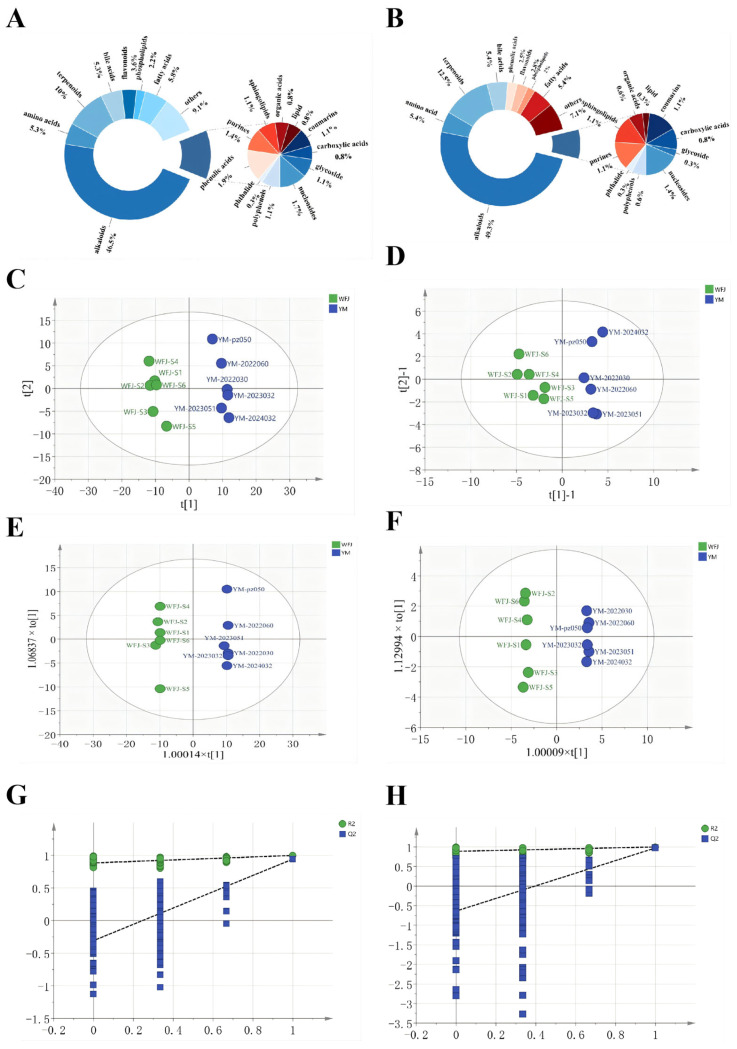
Dynamic changes of chemical components in the Yaomu during the fermentation process. (**A**) Unfermented Yaomu samples; (**B**) fermented Yaomu samples; (**C**) PCA score plots in positive; (**D**) PCA score plots in negative; (**E**) OPLS-DA score plots in positive; (**F**) OPLS-DA score plots in negative; (**G**) OPLS-DA permutation test plot in positive (200 times); (**H**) OPLS-DA permutation test plot in negative (200 times). Unfermented Yaomu samples are shown in green, and fermented Yaomu samples in blue.

In both positive and negative ionization modes, a total of 361 compounds were annotated in WFJ, including 168 alkaloids, 19 amino acids, 36 terpenoids, 19 bile acids, 4 polyphenols, 1 phthalide, 7 phenolic acids, 13 flavonoids, 8 phospholipids, 5 purines, 4 sphingolipids, 21 fatty acids, 3 organic acids, 3 lipids, 4 coumarins, 3 carboxylic acids, 4 glycosides, 6 nucleosides, and 33 other compounds. After fermentation, a total of 350 compounds were annotated, comprising 174 alkaloids, 19 amino acids, 44 terpenoids, 19 bile acids, 2 polyphenols, 1 phthalide, 9 phenolic acids, 10 flavonoids, 7 phospholipids, 4 purines, 4 sphingolipids, 19 fatty acids, 2 organic acids, 1 lipid, 4 coumarins, 3 carboxylic acids, 1 glycoside, 5 nucleosides, and 25 other compounds. Detailed data are provided in Tables S1–S4. The composite pie charts (Figure 1A,B) illustrate the distribution of compounds in Yaomu samples before and after fermentation. The annotated compounds encompassed diverse chemical classes, including alkaloids, terpenoids, lipids, polyphenols, and others. Consistent with Yaomu’s phytochemical profile, alkaloids constituted the most abundant class, accounting for over 45% of the total compounds. Terpenoids ranked second, contributing more than 10% of the detected compounds. The reduction in chemical components after fermentation may result from microbial enzymatic metabolism and changes in environmental conditions during the fermentation process, leading to their breakdown into simpler metabolic products or conversion into other structures [38,39].

### 2.2. Multivariate Statistical Analysis Results

#### 2.2.1. Principal Component Analysis

Principal Component Analysis (PCA) is an unsupervised multivariate statistical technique used to reduce the dimensionality of complex datasets while preserving as much variance as possible and to reflect the grouping patterns of the original samples [40]. A data matrix was constructed using the peak areas of common compounds annotated in 12 batches of Yaomu samples before and after fermentation. PCA was performed using SIMCA 14.1 (Sartorius Stedim Data Analytics AB, Umeå, Sweden). As shown in Figure 1C, under the positive ion mode, R^2^X [1] = 0.413 and R^2^X [2] = 0.112. The unfermented samples (WFJ) clustered in quadrants II and III, while fermented samples (YM) clustered in quadrants I and IV, indicating clear separation between groups. Under the negative ion mode (Figure 1D), R^2^X [1] = 0.397 and R^2^X [2] = 0.155. Similarly, unfermented samples distributed in quadrants II and III, and fermented samples in quadrants I and IV. These results suggest that there are notable differences in the chemical composition of Yaomu before and after processing under both positive and negative ionization modes.

#### 2.2.2. Orthogonal Partial Least Squares Discriminant Analysis

Orthogonal partial least squares discriminant analysis (OPLS-DA) was applied to identify and distinguish differential variables between groups, enhancing model interpretability and predictive performance [41]. The data matrix from Section 2.2.1 was subjected to OPLS-DA for further analysis. As shown in Figure 1E, under the positive ion mode, R^2^X = 0.517, R^2^Y = 0.998, and the Q^2^ value of the model was 0.942. Under the negative ion mode (Figure 1F), R^2^X = 0.612, R^2^Y = 0.998, and the Q^2^ value was 0.974. All three parameters exceeded 0.5, confirming good model stability and predictive ability under both positive and negative ionization modes. To assess whether the models were overfitted, 200 permutation tests were conducted. All the blue Q^2^ values on the left were lower than the original point on the right, and the regression line of the Q^2^ values intersected the vertical axis below zero, suggesting that the models were not overfitted and that the original models were valid [42] (Figure 1G,H). These results indicate significant differences in the chemical composition of Yaomu before and after processing under both ion modes. Differential compounds were screened based on the criteria: variable importance in projection (VIP) > 1, *p* < 0.05, and fold change (FC) ≥ 2 or ≤0.5. A total of 107 differential compounds were identified under the positive ion mode (Table S5). Among them, there were 52 alkaloids, 7 LPCs, 11 lipids and their derivatives, 4 bile acids, 8 terpenoids and sesquiterpenoids, 6 amino acids and amino lipids, and 19 other compounds.

Although high R^2^Y and Q^2^ values were obtained, several measures were implemented to reduce the risk of model overfitting. First, only compounds consistently detected in both pre- and post-fermentation samples were included (281 features in positive mode and 34 features in negative mode), which reduced noise introduced by missing variables. Second, permutation testing demonstrated that model performance was not driven by random class separation. Third, the multivariate analysis was applied primarily as an exploratory tool to visualize global metabolic differences rather than for predictive modeling. Therefore, the obtained models should be interpreted as acceptable exploratory representations of group separation rather than definitive predictive classifiers.

**Figure 2:**
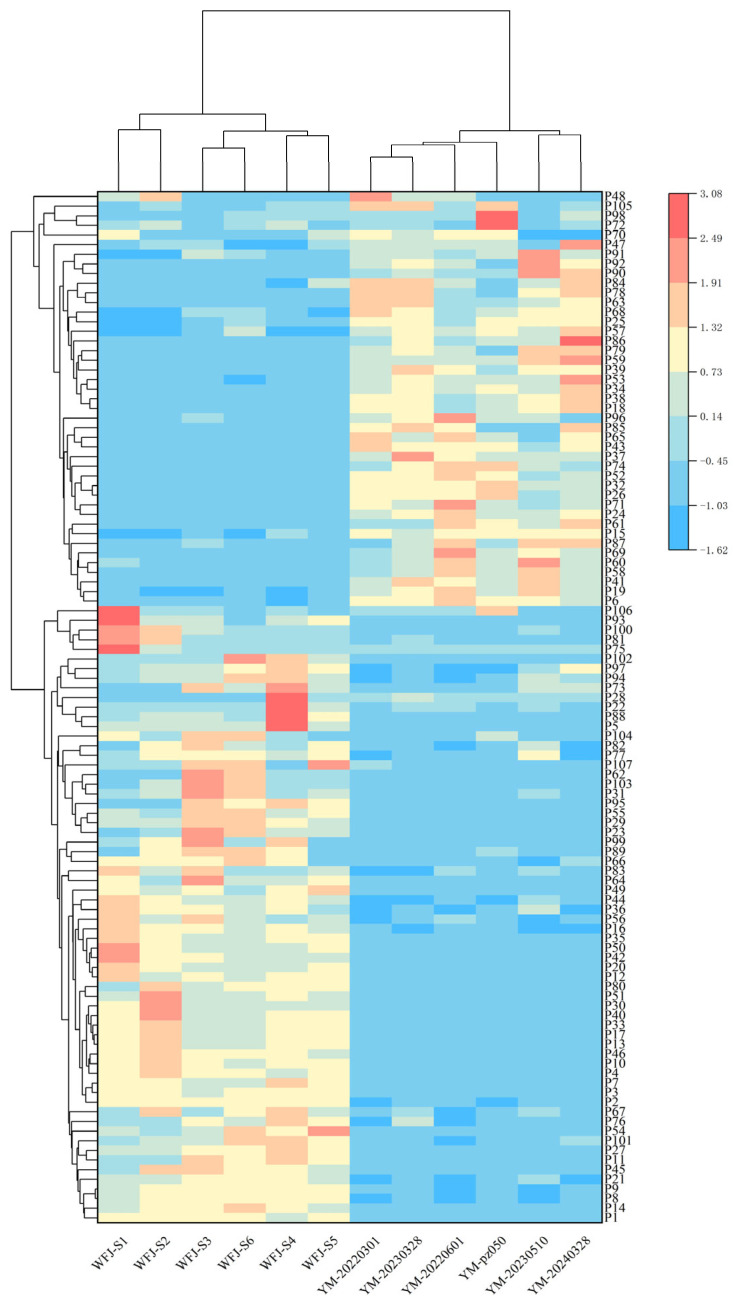
Heatmap of significantly differential compounds between unfermented Yaomu and fermented Yaomu. The horizontal axis represents 12 batches of Yaomu samples; the vertical axis represents differential compounds.

The 107 differential compounds identified through OPLS-DA analysis were further subjected to hierarchical cluster analysis (HCA) based on their relative content in Yaomu samples before and after fermentation. The clustering results clearly distinguished the pre-fermentation and post-fermentation groups, indicating significantly altered compound content due to fermentation. The heatmap visualization revealed consistent patterns within each group and distinct differences between groups, suggesting that these compounds could serve as a potential marker for monitoring chemical transformation or detoxification during fermentation (Figure 2).

### 2.3. Quantitative Analysis

To further screen potential toxic components, nine representative compounds were selected from these differential compounds based on statistical indicators (such as VIP values), low cost and easy availability, as well as literature evidence. The alkaloids included aconitine (AC), hypaconitine (HA), mesaconitine (MA), benzoylhypaconitine (BHA), benzoylmesaconitine (BMA), aconine (ACN), and hypaconine (HAN). The two LPCs analyzed were 1-Stearoyl-2-Hydroxysn-Glycero-3-Phosphatidylcholine (LPC (18:0)) and 1-oleoyl-2-hydroxy -sn-glycero-3-phosphocholine (LPC (18:1)).

#### 2.3.1. UHPLC-MS/MS Optimization

In this study, berberine (BBR) [43] and miltefosine (MF) [44] were chosen as internal standards (IS) due to their similar retention times to the target compounds under the applied analytical conditions. BBR closely matched the retention times of the alkaloid compounds, while MF was comparable to the lysophosphatidylcholine (LPC) compounds. Both internal standards demonstrated good chromatographic separation, signal intensity, and stability. Optimization solutions of the nine compounds and two ISs were injected into the mass spectrometer to optimize MS signal intensities. Following optimization under both positive and negative ion modes, it was observed that all compounds exhibited stronger signal intensities in the positive ion mode. Key mass spectrometric parameters—including Quadrupole 1 (Q1), Quadrupole 3 (Q3), declustering potential (DP), collision energy (CE), entrance potential (EP), and collision cell exit potential (CXP)—were optimized to ensure sensitive MRM (multiple reaction monitoring) transitions. The optimized MRM parameters for each compound are listed in Table S6.

#### 2.3.2. Specificity

UHPLC-MS/MS analysis was performed on mixed standard solutions, WFJ sample solutions, and YM sample solutions. As shown in Figure S2, there was no overlap between the chromatographic peaks of the target analytes and internal standards. No significant endogenous interference peaks were observed near the retention times of the analytes and internal standards, indicating that the method possesses good specificity.

#### 2.3.3. Linearity and Sensitivity

The linearity range and sensitivity for analysis of nine alkaloids and two LPCs were evaluated with standard solution and listed in Table S7. Good linearity at the respective ranges for all analytes were obtained with R^2^ ≥ 0.9900. The LOQs for AC, HA, MA, BHA, BMA, ACN, HAN, LPC (18:0) and LPC (18:1) were 0.05, 0.05, 0.1, 0.05, 0.10, 0.05, 0.05, 0.1 and 0.05 ng/mL, respectively. The LODs for AC, HA, MA, BHA, BMA, ACN, HAN, LPC (18:0) and LPC (18:1) were 0.015, 0.015, 0.03, 0.015, 0.03, 0.015, 0.015, 0.03, and 0.015 ng/mL, respectively.

#### 2.3.4. Precision, Accuracy, and Stability

The intra- and inter-day precisions, accuracies, and stabilities of the method were evaluated for nine samples at three quality control concentration levels. As shown in Table S8, satisfactory intra- and inter-day precision, accuracies, and stabilities were achieved, with relative standard deviations (RSD) ≤ 7.45%. The accuracy of the tests ranged from 85% to 115%. These findings indicate that the method has good intra-day precision, inter-day precision, and stability. Therefore, the method is reliable, reproducible, accurate, and stable.

#### 2.3.5. Repeatability

Six WFJ-S4 samples were taken, and the contents of AC, HA, MA, BHA, BMA, ACN, HAN, LPC (18:0), and LPC (18:1) were calculated according to the standard curve. As shown in Table S9, the RSD for the nine compounds in WFJ was ≤2.68%, indicating that the method exhibits good repeatability.

#### 2.3.6. Extration Recovery

As shown in Table S10, under the conditions of adding standard samples at three concentration levels (high, medium, and low), the accuracy of the extraction recovery rate for the nine compounds ranged from 90.93% to 105.44%, with an RSD ≤ 7.77%. These results meet the extraction recovery standards, demonstrating that the method exhibits good extraction recovery performance.

#### 2.3.7. The Content of 9 Compounds in Yaomu

Six batches each of YM and WFJ samples were analyzed, and the contents of AC, HA, MA, BHA, BMA, ACN, HAN, LPC (18:0), and LPC (18:1) were calculated according to the standard curves, as shown in Table S11. The results indicated that after fermentation, the AC content in the six YM batches ranged from 0.53 μg/g to 2.84 μg/g, HA from 18.95 μg/g to 41.64 μg/g, MA from 1.01 μg/g to 6.39 μg/g, BHA from 25.32 μg/g to 40.23 μg/g, BMA from 60.37 μg/g to 100.81 μg/g, ACN from 3.65 μg/g to 13.41 μg/g, HAN from 5.76 μg/g to 31.74 μg/g, LPC (18:0) from 0.04 μg/g to 0.69 μg/g, and LPC (18:1) from 0.41 μg/g to 3.03 μg/g. Before fermentation, the AC content in the six WFJ batches ranged from 8.79 μg/g to 19.13 μg/g, HA from 71.11 μg/g to 177.35 μg/g, MA from 20.50 μg/g to 84.89 μg/g, BHA from 10.09 μg/g to 26.35 μg/g, BMA from 108.70 μg/g to 179.27 μg/g, ACN from 1.52 μg/g to 3.40 μg/g, HAN from 1.34 μg/g to 3.51 μg/g, LPC (18:0) from 35.87 μg/g to 58.40 μg/g, and LPC (18:1) from 142.98 μg/g to 594.67 μg/g.

**Figure 3:**
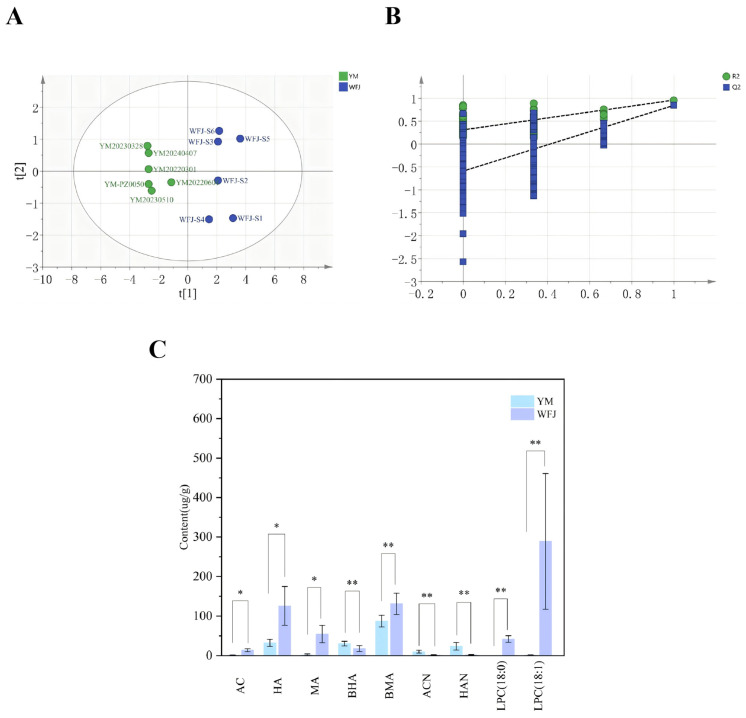
Multivariate statistical analysis of compound content before and after Yaomu fermentation. (**A**) PCA score plot showing overall metabolic differences between pre- and post-fermentation samples. Pre-fermentation samples are shown in blue, and post-fermentation samples in green; (**B**) 200-time permutation test of the OPLS-DA model, where all permuted Q^2^ values are lower than the original, and the regression line intercepts the vertical axis below zero, indicating model stability without overfitting; (**C**) bar chart of the content of 9 representative compounds (mean ± SD), with significance levels indicated as * *p* < 0.05 and ** *p* < 0.01. Pre-fermentation samples are shown in purple, and post-fermentation samples in blue.

PCA and OPLS-DA were applied to the quantitative data of potential toxic constituents in Yaomu before and after fermentation. As shown in Figure 3A, WFJ samples are distributed in quadrants I and IV, while YM samples are distributed in quadrants II and III, demonstrating a clear distinction. The PCA score plot showed a clear clustering trend, with distinct separation between the unfermented and fermented groups, suggesting substantial differences in potentially toxic compound profiles. OPLS-DA was applied for further analysis, yielding R^2^X = 0.802, R^2^Y = 0.958, and a Q^2^ value of 0.843. All three parameters exceeded 0.5, indicating that the model exhibited good stability and predictive ability (Figure 3B). Potential toxic components showed significantly decreased or transformed concentrations after fermentation, suggesting that the fermentation process may contribute to the detoxification of Yaomu (Figure 3C). These results provide evidence that fermentation plays a key role in modulating the toxicity of Yaomu through changes in its chemical composition.

### 2.4. Liver On-a-Chip Results

#### 2.4.1. Cytotoxicity Test

**Figure 4:**
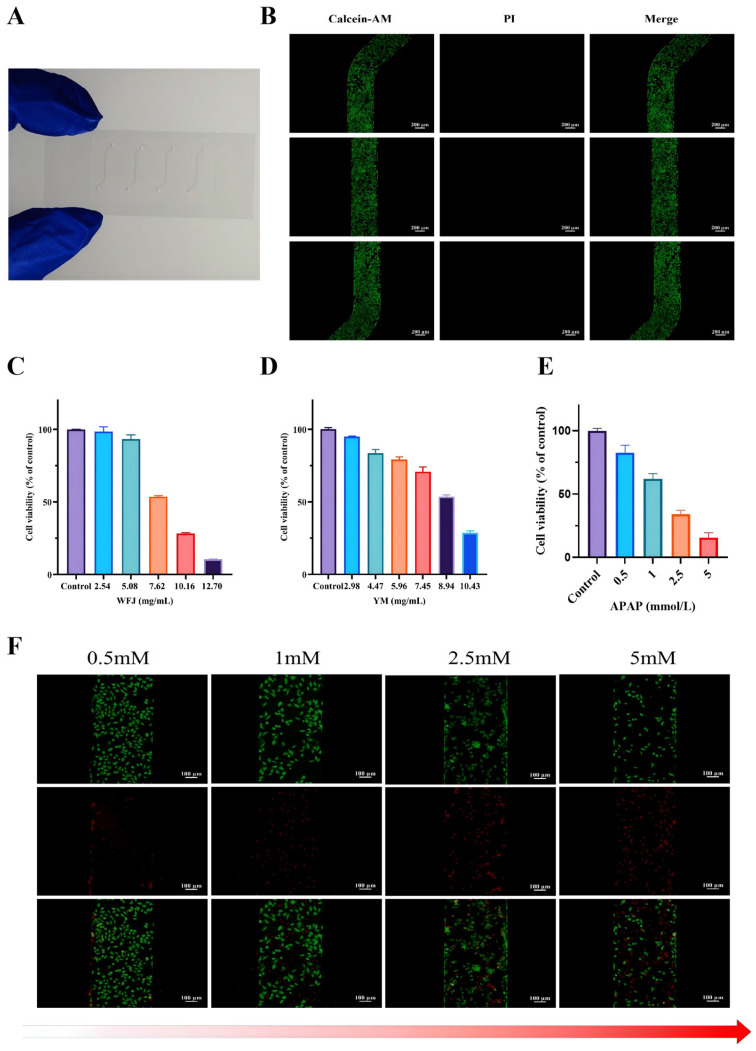
Structure and function verification of liver-on-a-chip. (**A**) Fabricated liver-on-a-chip. (**B**) Fluorescence imaging of cell viability levels for THLE-2 cells cultured with liver-on-a-chip model. (**C**) Cell viability stimulated by WFJ with different concentration. (**D**) Cell viability stimulated by YM with different concentration. (**E**) Cell viability stimulated by APAP with different concentration. (**F**) Immunofluorescent images of levels of live and dead THLE-2 cells stimulated by APAP with different concentration in liver-on-a-chip model.

The cytotoxic effects of unfermented and fermented Yaomu on THLE-2 cells were evaluated using the CCK-8 colorimetric assay. As shown in Figure 4C,D, cell viability exhibited a clear concentration-dependent decrease after 24 h of exposure to different doses of unfermented and fermented Yaomu. Since the yields of Yaomu before and after fermentation vary greatly, the administration concentration is converted into the amounts of crude drugs for calculation and comparison. The IC_50_ values of the unfermented and fermented Yaomu were 7.87 mg/mL and 9.98 mg/mL (crude drug equivalents). According to this result, the liver-on-a-chip was randomly divided into three groups: control, unfermented Yaomu (WFJ, 7.8 mg/mL), and fermented Yaomu (YM, 7.8 mg/mL).

#### 2.4.2. Function Verification of Liver-on-a-Chip

This study detected the viability of THLE-2 cells after 24 h of dynamic perfusion inoculation. Most cells maintained strong physiological activity (Figure 4B). The results indicate that this microfluidic chip is suitable for in vitro cell culture, can be used for subsequent research, and has no obvious cytotoxicity. In addition, different concentrations of Acetaminophen (APAP) (0.5, 1, 2.5, 5 mM) also had obvious hepatotoxic effects on THLE-2 cells, and there was a dose-dependent relationship (Figure 4E,F). Therefore, our method effectively established a liver-on-a-chip model that can be used for the assessment of hepatotoxicity of drugs.

#### 2.4.3. Function Verification of Liver-on-a-Chip Results

**Figure 5:**
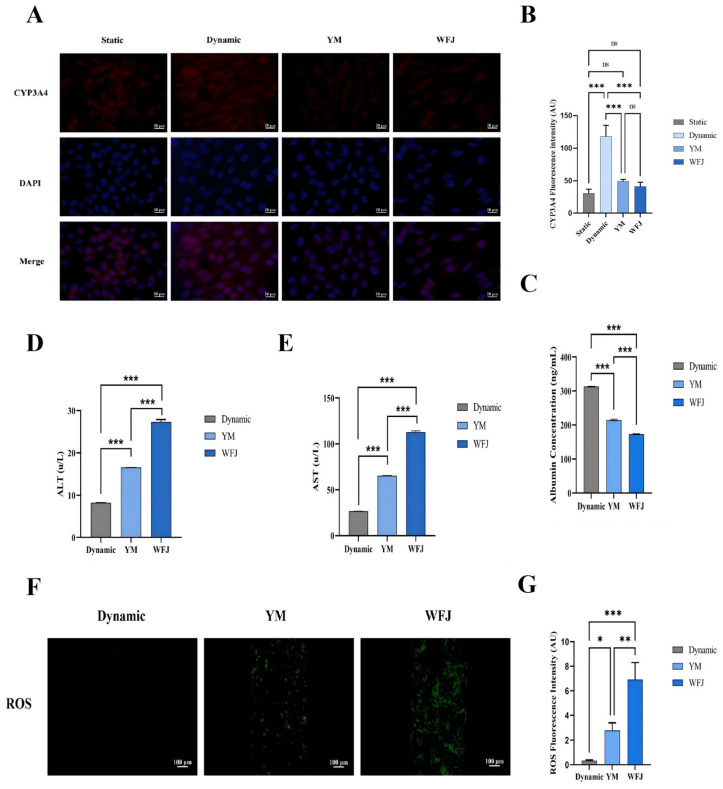
Function verification and Oxidative stress of liver-on-a-chip. (**A**) Fluorescence imaging of CYP3A4 for THLE-2 cells cultured with liver-on-a-chip model. (**B**) Semi-quantitative results of CYP3A4 immunofluorescence. (**C**) After 12 h of dynamic perfusion in the blank group, perfusion administration of WFJ (7.8 mg/mL) and YM (7.8 mg/mL), and the released amount of ALB. (**D**) After 12 h of dynamic perfusion in the blank group, perfusion administration of WFJ (7.8 mg/mL) and YM (7.8 mg/mL), and the released amount of ALT. (**E**) After 12 h of dynamic perfusion in the blank group, perfusion administration of WFJ (7.8 mg/mL) and YM (7.8 mg/mL), and the released amount of AST. (**F**) Fluorescence imaging of ROS for THLE-2 cells cultured with liver-on-a-chip model. (**G**) Semi-quantitative results of ROS immunofluorescence. Significance levels indicated as * *p* < 0.05, ** *p* < 0.01, and *** *p* < 0.001.

First, hepatic function of the liver on-a-chip was evaluated. The expression levels of CYP3A4 and albumin were compared among liver on-a-chip models under static culture, dynamic culture, dynamic perfusion with WFJ, and dynamic perfusion with YM. Compared with static culture, dynamic perfusion significantly increased CYP3A4 expression and albumin secretion in the liver on-a-chip, indicating enhanced hepatic metabolic and synthetic functions under physiologically relevant flow conditions. Therefore, all subsequent toxicity evaluations were performed under dynamic perfusion conditions. Compared with the dynamically perfused blank, both WFJ and YM significantly reduced CYP3A4 expression in the liver on-a-chip, with no significant difference observed between the two treatments. In contrast, albumin secretion was markedly decreased in both groups; however, the reduction was significantly attenuated in the YM chips, which exhibited higher albumin levels than those treated with WFJ (Figure 5A,C). This finding suggests that CYP3A4 is highly sensitive to the overall chemical exposure and may not directly reflect differences in toxicity severity. In contrast, albumin secretion, which is closely associated with hepatocellular integrity and synthetic function, was markedly less reduced in the fermented Yaomu group, indicating an improved cellular functional state following fermentation.

Serum Alanine Aminotransferase (ALT) and Aspartate Aminotransferase (AST) are widely recognized biomarkers of hepatocellular injury. In this study, ALT and AST levels were systematically compared among dynamically cultured control liver chips, YM–treated chips, and WFJ–treated chips under dynamic perfusion conditions. In liver chips subjected only to dynamic perfusion, ALT levels remained below 10 U/L, indicating stable hepatic function and preserved cellular integrity. Following drug perfusion, ALT levels increased to approximately 10–20 U/L in the YM group, whereas a further elevation was observed in the WFJ group (Figure 5D). Consistent with the changes in ALT, AST levels were also significantly increased after drug exposure, with the WFJ group exhibiting a more pronounced elevation compared with the YM group (Figure 5E). These results indicate differential degrees of hepatocellular injury induced by YM and WFJ under dynamic perfusion conditions.

**Figure 6:**
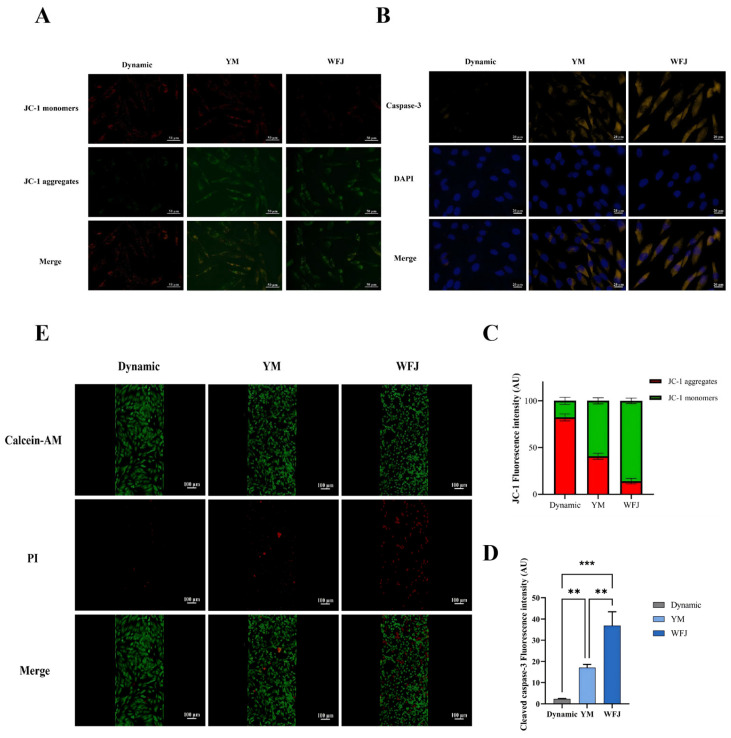
Mitochondrial damage and apoptosis of liver-on-a-chip. (**A**) Fluorescence imaging of JC-1 for THLE-2 cells cultured with liver-on-a-chip model. (**B**) Fluorescence imaging of cleaved caspase-3 for THLE-2 cells cultured with liver-on-a-chip model. (**C**) Semi-quantitative results of JC-1 immunofluorescence. (**D**) Semi-quantitative results of cleaved caspase-3 immunofluorescence. (**E**) Immunofluorescence images of the levels of alive and dead THLE-2 cells stimulated by the dynamic perfusion blank group, YM group (7.8 mg/mL), and WFJ (7.8 mg/mL) in the liver-on-a-chip. Significance levels indicated as ** *p* < 0.01, and *** *p* < 0.001.

Intracellular reactive oxygen species (ROS) levels were significantly increased in liver on-a-chip models treated with both unfermented and fermented Yaomu compared with the dynamically perfused blank. Notably, the fluorescence intensity of ROS was markedly higher in the WFJ group than in the YM group (Figure 5F,G). Moreover, JC-1 staining was performed to assess changes in mitochondrial membrane potential (ΔΨm) in the liver-on-a-chip model. In the control group, JC-1 fluorescence was predominantly red, indicating intact mitochondrial membrane potential with no obvious depolarization. Following treatment with YM, a reduction in mitochondrial membrane potential was observed, as reflected by a mixed distribution of red and green fluorescence, with approximately comparable proportions. In contrast, treatment with WFJ resulted in a more pronounced loss of mitochondrial membrane potential, characterized by a predominance of green fluorescence (Figure 6A,C).

Compared with the dynamically perfused blank, caspase-3 fluorescence intensity was significantly increased in liver organ-on-a-chip models treated with both unfermented and fermented Yaomu, indicating activation of apoptosis. Notably, the WFJ group exhibited markedly stronger caspase-3 fluorescence than the YM group (Figure 6B,D), suggesting that fermentation attenuated Yaomu-induced apoptotic signaling in hepatocytes. Calcein-AM and propidium iodide (PI) were used as dual fluorescent probes to evaluate apoptosis induced by unfermented and fermented Yaomu, with viable cells emitting green fluorescence and dead cells exhibiting red fluorescence. After perfusion treatment, the apoptotic rate of THLE-2 cells was significantly increased. Notably, the cell death rates in the dynamically perfused blank control and YM groups were lower than those in the WFJ group (Figure 6E), suggesting that fermented Yaomu may exhibit a reduced capacity to induce cell apoptosis.

## 3. Discussion

As the monarch drug of HFD, Yaomu is an important component for HFD to exert its therapeutic effects. It is a representative Ethnic medicine with both toxic and therapeutic effects. Since most of its components are toxic Chinese herbal medicines, its clinical application is restricted. Appropriate processing methods to reduce toxicity and preserve efficacy are the key to the safe use of Yaomu. The key to reducing toxicity lies in the change of chemical components before and after processing. This study is the first to systematically analyze the complex chemical composition of Huafeng Dan’s monarch drug, Yaomu, before and after bovine bile-mediated fermentation using a multi-dimensional UHPLC-Q/TOF-MS strategy. A total of 361 and 350 compounds were annotated before and after fermentation, respectively, with 107 differential compounds revealed through untargeted screening and multivariate analysis. In addition, based on statistical indicators (e.g., VIP values), low cost and easy accessibility, and literature evidence, nine chemical constituents were selected for LC-MS quantitative analysis, including two diester-type diterpenoid alkaloids, two monoester-type diterpenoid alkaloids, two alcoholamine-type diterpenoid alkaloids, and two LPCs. A rapid and sensitive LC-MS/MS method was established to simultaneously quantify these nine compounds in Yaomu extracts before and after fermentation. The developed analytical method demonstrated high specificity, sensitivity, precision, and accuracy in quantifying these nine target compounds. Comparative analysis of quantitative results revealed significant compositional differences between six batches of YM and six batches of WFJ. Post-fermentation, the contents of diester-type diterpenoid alkaloids (AC, HA and MA), monoester-type diterpenoid alkaloids (BMA), and LPCs (LPC (18:0) and LPC (18:1)) in six batches of YM were significantly lower than those in six batches of WFJ (* *p* < 0.05, ** *p* < 0.01, ** *p* < 0.01), while concentrations of monoester-type diterpenoid alkaloids (BHA) and alcoholamine-type diterpenoid alkaloids (ACN and HAN) showed a statistically significant increase (** *p* < 0.01, ** *p* < 0.01). This result indicates that fermentation has a significant regulatory effect on the chemical composition of Yaomu, effectively reducing the proportion of highly toxic components. These observations do not explicitly prove a detoxification mechanism but rather elucidate the step-wise bioconversion pathway: diester-type diterpenoid alkaloids undergo bovine bile-mediated fermentation to form less toxic monoester-type derivatives, which are subsequently transformed into minimally toxic alcoholamine-type alkaloids. These findings provide key insights into the transformation process of Yaomu during fermentation.

LPCs are endogenous phospholipid derivatives that are widely present in plasma and cell membranes and play important roles in lipid transport and signal transduction processes. Different from aconitine-type diterpenoid alkaloids, which have well-defined intrinsic toxicity, LPC substances are not the inherent toxic components of Yao medicines. However, increasing evidence suggests that changes in LPC levels reflect the disorder of membrane phospholipid metabolism during cell stress, inflammation, and oxidative damage [45]. In this context, LPCs may participate as downstream mediators of inflammatory signaling and membrane remodeling rather than as major toxic substances. Therefore, in this study, LPC (18:0) and LPC (18:1) were interpreted as toxicity-related metabolic indicators or co-mediators, reflecting the biochemical responses to toxic exposure and fermentation-induced metabolic reprogramming rather than the direct toxic components of Yaomu themselves. This distinction provides a more mechanistically consistent framework for understanding the observed lipidomic changes.

Given the complex chemical composition of Yaomu, in which aconitine-type alkaloids represent only one of the major constituent classes among numerous coexisting components, a comprehensive toxicity evaluation is required. Because the liver serves as the major site of first-pass metabolism, it is frequently the primary target organ for drug-induced toxicity, highlighting the importance of physiologically relevant liver models for safety evaluation. A microfluidic liver-on-a-chip platform was established in this study to evaluate hepatotoxicity before and after fermentation. The liver-on-a-chip system provides continuous perfusion, enabling physiologically relevant shear stress, enhanced oxygen and nutrient delivery, and efficient metabolic waste removal, thereby supporting prolonged maintenance of hepatocyte viability and function. Increasing evidence has shown that microfluidic liver-chip platforms maintain higher albumin secretion, improved cell polarity, and enhanced cytochrome P450 enzyme activities compared with static cultures, reflecting a more in vivo-like metabolic competency essential for reliable hepatotoxicity assessment [46,47]. Collectively, these advantages highlight liver-on-a-chip technology as a more physiologically relevant and predictive in vitro platform, bridging the gap between traditional culture models and in vivo studies.

Using this platform, fermentation with bovine bile was associated with attenuated hepatotoxic responses compared with unfermented materials. Under dynamic perfusion alone, liver chips maintained low ALT levels (<10 U/L), reflecting preserved membrane integrity and stable hepatic function, whereas drug exposure induced enzyme leakage in a preparation-dependent manner. Both ALT and AST levels were elevated following treatment, with markedly greater increases observed in the WFJ group compared with the YM group, indicating more severe hepatocellular damage induced by the unfermented materials. Consistent with these biochemical alterations, hepatocytes in the WFJ group exhibited a constellation of injury phenotypes, including reduced albumin secretion, excessive ROS accumulation, loss of mitochondrial membrane potential as indicated by JC-1 staining, increased caspase-3 activation, and higher proportions of dead cells in live/dead assays. These observations are consistent with oxidative stress-associated mitochondrial dysfunction and activation of apoptosis-related pathways; however, the present data do not establish a direct causal sequence linking individual chemical components to specific toxicity mechanisms.

CYP3A4 expression and albumin secretion, commonly used indicators of hepatocyte metabolic competence, were reduced in the WFJ group, further suggesting impaired hepatocellular function under these conditions. In contrast, fermented samples were associated with comparatively milder alterations in biochemical, oxidative stress, and apoptosis-related endpoints. Collectively, the current results should be interpreted cautiously as associative observations rather than definitive mechanistic proof. Given the limited number of targeted markers and the absence of dedicated mechanistic validation experiments, the present study should be regarded as providing hypothesis-generating evidence supporting a potential relationship between fermentation-induced chemical transformation and altered hepatotoxic responses.

## 4. Materials and Methods

### 4.1. Chemicals and Reagents

Methanol (HPLC grade) was purchased from Shanghai Xingke High Purity Solvents Co., Ltd. (Shanghai, China). Formic acid (HPLC grade) was purchased from Aladdin Biochemical Technology Co., Ltd. (Shanghai, China). Acetonitrile (LC-MS grade) was purchased from Merck (Darmstadt, Germany). Deionized water was further purified using a Milli-Q system (Millipore, Billerica, MA, USA). For alkaloid determination, aconitine (No. BBP03866, 98%), hypaconitine (No. BBP03972, 98%), mesaconitine (No. BBP04014, 98%), benzoylhypaconine (No. BBP60533, 98%), benzoylmesaconine (No. BBP60534, 98%), aconine (No. BBP60412, 98%), hypaconine (No. BBP60695, 98%), and berberine (No. BBP01161, 98%) were purchased from Yunnan Xili Biotechnology Co., Ltd. (Yunnan, China). For phospholipid determination, LPC (18:0) (No. 24W110-L3, 95.11%), LPC (18:1) (No. 24W139-V3, 99.5%), and Miltefosine (No. 24W216-Y6) were purchased from Shanghai Zhenzhun Biological Technology Co., Ltd. (Shanghai, China). APAP (No. AFCC1554, 98%) was purchased from Chengdu Efa Biotechnology Co., Ltd. (Chengdu, China). All analytical standards were stored at −20 °C. A total of 12 Yaomu samples were kindly provided by Zunyi Liaoyuan Hetang Pharmaceutical Co., Ltd. (Zunyi, China), including six samples before fermentation and six samples after fermentation (No. YM20220301, No. YM20230510, No. YM20220601, No. YM20230328, No. YM-PZ0050, No. YM20240407, No. WFJ-S1, No. WFJ-S2, No. WFJ-S3, No. WFJ-S4, No. WFJ-S5, No. WFJ-S6).

The THLE-2 cell line and the dedicated culture medium were acquired from Shanghai EK-Bioscience Biotechnology Co., Ltd. (Shanghai, China). The organ-on-a-chip was self-made within the research group. The human albumin ELISA Kit (J1876) was purchased from Wuhan Jilide Biotechnology Co., Ltd. (Wuhan, China). The CYP3A4 rabbit polyclonal antibody (pAb) was purchased from Abclonal (Cat. No. A2544), the Cy3-conjugated goat anti-rabbit IgG secondary antibody was obtained from Absin (Cat. No. abs20024), and the cleaved caspase-3 polyclonal antibody was purchased from Proteintech (Cat. No. 25128-1-AP). 6-Carboxy-2′,7′-dichlorodihydrofluorescein (DCFH-DA) diacetate was purchased from Thermo Fisher (Waltham, MA, USA). Type I mouse tail collagen was purchased from Shanghai Yuanye Bio-Technology Co., Ltd. (Shanghai, China). Calcein AM/PI (Cat. No. C1371M), JC-1 (Cat. No. C2006), 4′,6-Diamidino-2-phenylindole dihydrochloride (DAPI) (Cat. No. C1006), Amplex Red Aspartate Aminotransferase Activity Assay Kit (Cat. No. P2715S) and Amplex Red Alanine Aminotransferase Activity Assay Kit (Cat. No. P2711S) were both purchased from Beyotime Biotechnology Co., Ltd. (Shanghai, China).

### 4.2. Laboratory Apparatus

Chromatographic separation was achieved using a Shimadzu LC system (Shimadzu Corporation, Kyoto, Japan). High-resolution mass spectrometric data were acquired on an AB Sciex X500R quadrupole time-of-flight mass spectrometer (AB Sciex LLC, Framingham, MA, USA). Quantification was performed on an AB Sciex 4500 triple quadrupole mass spectrometer (AB Sciex LLC, Framingham, MA, USA). Enzyme-labeled detector (Model: VarioskanFlash; Manufacturer: ThermoScientific). An enzyme-labeled detector (Model: Varioskan™ Flash, Thermo Fisher Scientific, Waltham, MA, USA) was used for absorbance measurements. Sample extraction was performed using an ultrasonic cleaner (Model: KQ-500DE, Kunshan Ultrasonic Instruments Co., Ltd., Beijing, China). Samples were freeze-dried using a stoppering-type freeze dryer (Model: LGJ-18, Manufacturer: Beijing Sihuan Scientific Instrument Factory Co., Ltd., Beijing, China). A microcentrifuge (Model: Fresco™ 21, Manufacturer: Thermo Fisher Scientific, Waltham, MA, USA) was used for sample centrifugation. Chip perfusion was performed using a syringe pump (Model LSP02-2Y, Baoding Rongbai Constant Flow Pump Manufacturing Co., Ltd., Hebei, China). Images were acquired using an automated inverted fluorescence microscope (Model: Axio Observer 7, Manufacturer: Zeiss, Jena, Germany) equipped with a digital camera and ZEN imaging software (version 3.8, Carl Zeiss Microscopy GmbH, Jena, Germany).

### 4.3. Sample Preparation

Twelve samples were pulverized and passed through a No. 5 mesh sieve. Exactly 5.0 g of each sample was weighed and placed into a conical flask, followed by the addition of 50 mL of 70% Methanol in Water (*v*/*v*). After recording the weight and mixing thoroughly, the mixture was ultrasonicated at 80 W for 30 min. The samples were cooled to room temperature, and the lost weight was replenished. The solution was transferred to a centrifuge tube and centrifuged at 5000 r/min for 10 min. The supernatant was filtered through a 0.22 μm microporous membrane, with the first three drops discarded. The remaining filtrate was collected into HPLC vials and stored at 4 °C for further analysis.

### 4.4. Multivariate Statistical Analysis

#### 4.4.1. UHPLC-Q-TOF-MS Analysis

The instrument used was UPLC-Q-TOF-MS with an Acquity™ UPLC HSS T3 column (dimensions: 150 mm × 2.1 mm, and 1.8 μm particle size; Milford, MA, USA). The column oven temperature was maintained at 35 °C with a temperature limit of 90 °C to ensure optimal separation conditions. The mobile phase composition comprised a mixture of H_2_O (A, containing 0.1% formic acid) and acetonitrile (B) with a gradient of mobile phase as follows: 0–15 min, 98%~90% A; 15–30 min, 90%~80% A; 30–40 min, 80%~70% A; 40–50 min, 70%~50% A; 50–55 min, 50%~35% A; 55–58 min,35%~30% A; 58–64 min, 30–10% A; 64–66 min, 10–10% A.

#### 4.4.2. Mass Spectrometry Conditions

Sampling was performed using an electrospray ionization (ESI) source in full scan mode under both positive and negative ion modes. The ion spray voltages were set at 5500 V for positive mode and −4500 V for negative mode. The ion source temperature was maintained at 550 °C. The nebulizer gas (N_2_) and drying gas (N_2_) pressure were set at 55 psi, and the curtain gas (N_2_) pressure was 35 psi. In TOF-MS mode, the scan range was *m*/*z* 50–1500 Da, with a declustering potential (DP) of 80 V (positive ion mode) or −80 V (negative ion mode), and a collision energy (CE) of 10 V (positive) or −10 V (negative). In TOF-MS/MS mode, the scan range remained *m*/*z* 50–1500 Da, with the same declustering potential settings (DP: 80 V/−80 V), while the collision energy was set to 35 V (positive) or −35 V (negative).

#### 4.4.3. Analysis of Differential Compounds Before and After Fermentation

Samples of Yaomu before and after fermentation from were analyzed by UHPLC-Q-TOF-MS under the chromatographic and mass spectrometric conditions described in Section 4.4.1 and Section 4.4.2. The SCIEX OS software’s predicted molecular formula function was employed to predict molecular formulas and match them with characteristic fragment ions for chemical structure identification. The mass spectrometry data of Yaomu before and after fermentation were processed using MS Convert and MS-DIAL software. The processed data were subsequently imported into SIMCA 14.1 for PCA and OPLS-DA analyses.

#### 4.4.4. Qualitative Data Analysis

MS/MS fragmentation data were manually interpreted and compiled using Microsoft Word 365 (Microsoft Corp., Redmond, WA, USA), including retention times, fragment ions, and structural annotations. Multivariate statistical analysis including principal components analysis (PCA) and orthogonal partial least-squares discriminant analysis (OPLS-DA) was performed to visualize the metabolic variations using SIMCA-P v.14.1 (Umetrics, Umeå, Sweden). Extracted ion chromatograms (EICs), a pie chart, and hierarchical cluster analysis (HCA) were generated using OriginPro^®^ 2025 (Origin Lab Corporation, Northampton, MA, USA).

### 4.5. Quantitative Method Validation

This study did not involve quantitative analysis in biological matrices. All calibration and quantitative analyses were performed using traditional herbal extract solutions rather than biomaterials such as plasma, tissue, or other biological fluids.

#### 4.5.1. Standard and Sample Solutions

Optimization Solution: An optimization solution containing 500 ng/mL of AC, HA, MA, BHA, BMA, ACN, HAN, LPC (18:0), and LPC (18:1) was prepared in 70% Methanol in Water (*v*/*v*). BBR and MF were selected as internal standards (IS), with BBR serving as the internal standard for AC, HA, MA, BHA, BMA, ACN, and HAN, and MF for LPC (18:0) and LPC (18:1). A standard optimization solution (500 ng/mL) containing all target analytes and internal standards was infused into the mass spectrometer to optimize MRM parameters, including mass spectrometry parameters such as Q1, Q3, DP, CE, EP, and CXP.

Standard solution: A primary stock solution containing all reference standards was prepared by dissolving 1.0 mg of each standard compound in methanol. According to records from PubChem and Pharmacopoeia of the People’s Republic of China, aconitum alkaloids are described as readily soluble in methanol and ethanol (>100 mg/mL). The LIPID MAPS database classifies LPC lipids as soluble in methanol (5–50 mg/mL). To establish calibration curves with concentrations ranging from 1 to 750 ng/mL, the stock solution was serially diluted with 70% methanol (*v*/*v*). This process generated nine calibration points. The primary stock solution was stored at −20 °C for future use. No precipitation or phase separation was observed during storage within the study period.

Quality Control (QC) Samples: QC samples were prepared from the primary stock solution. Appropriate aliquots of the stock solution were diluted appropriately with 70% Methanol (*v*/*v*) to prepare low, medium, and high QC samples at final concentrations of 5 ng/mL, 50 ng/mL, and 500 ng/mL, respectively.

Test Samples: Test samples were prepared as described in Section 4.3.

#### 4.5.2. LC-MS Analysis of Yaomu

The UPLC-QQQ-MS/MS systems consisted of an HPLC separation system (Shimadzu, Kyoto, JPN) and an API 4500 Qtrap mass spectrometer equipped with an ESI interface (AB Sciex, Framingham, MA, USA). Chromatographic separation was conducted using an ACQUITY^TM^ Premier BEH C18 column (2.1 × 100 mm, and 1.7 μm particle size, Waters Corp., Milford, MA, USA) with column temperature maintained at 35 °C. Mobile phase A consisted of 0.1% formic acid in water (*v*/*v*), and mobile phase B was acetonitrile. The injection volume was set to 3 μL, and the column was re-equilibrated at initial gradient conditions for 10 min. The gradient elution program with a flow rate of 0.3 mL/min was used as follows: 0–1 min, 10%~20% B; 1–8 min, 20%~35% B; 8–10 min, 35%~60% B; 10–15 min, 60%~90%. The analysis was performed using electrospray ionization (ESI) in positive ion mode. Quantification was carried out using the multiple reaction monitoring (MRM) mode. The source parameters were set as follows: curtain gas (CUR), 35 psi; collision gas (CAD), 9; ion spray voltage (IS), 5500 V; nebulizer gas (GS1) and auxiliary gas (GS2), 45 psi each; and source temperature (TEM), 500 °C.

#### 4.5.3. Method Validation

This study evaluated key analytical parameters including linearity (R^2^), limit of quantification (LOQ), limit of detection (LOD), accuracy, stability, repeatability, extraction recovery (%), and relative standard deviation (RSD).

Linearity and Calibration Curve: The linearity of the method was evaluated by analyzing a series of standard solutions at concentrations ranging from 1 to 750 ng/mL. Calibration curves were constructed by plotting the peak area ratio to internal standard versus the known concentrations. A least-squares linear regression analysis was performed. The linearity was considered acceptable when the correlation coefficient (R^2^) exceeded 0.99.

Sensitivity: The limit of detection (LOD) and limit of quantification (LOQ) were determined based on the signal-to-noise ratio (S/N). LOD was defined as the concentration with an S/N ratio of 3:1, while LOQ was defined as the concentration with an S/N ratio of 10:1.

Precision, Accuracy and Stability: Intra-day precision was determined by analyzing QC samples at three different concentrations within a single day, while inter-day precision was assessed by analyzing the same QC samples on three different days. Sample stability was conducted by storing the QC samples in an autosampler at 15 °C, with injections performed at 0, 2, 4, 8, 12, and 24 h.

Repeatability: To evaluate the repeatability of the method, a single batch of sample was divided into six aliquots and each aliquot was analyzed independently under identical conditions. The relative standard deviation (RSD%) of the measured results was used to assess repeatability.

Extraction Recovery: Extraction recovery was assessed by comparing the peak areas of analytes extracted from samples with those of post-extraction spiked samples at equivalent concentrations. Recovery (%) = (Measured value of Spiked Sample − Measured value of non-spiked Sample/Nominal spiked concentration) × 100.

#### 4.5.4. Quantitative Data Analysis

All analytical data were processed using Analyst^®^ software 1.7 (SCIEX, Framingham, MA, USA). Results were organized using Microsoft Excel 365 (Microsoft Corporation, Redmond, WA, USA), and expressed as the mean (±SD). Statistical analysis was performed using SPSS version 27 (IBM Corp., Armonk, NY, USA). When the data satisfied the assumption of normal distribution, an independent samples *t*-test was performed; otherwise, the Mann–Whitney *U* test was applied to assess the statistical significance of differences in the same compounds between the pre- and post-fermentation groups. A *p*-value of less than 0.05 was considered statistically significant.

### 4.6. Liver On-a-Chip

#### 4.6.1. Preparation of Yaomu Before and After Fermentation for Liver On-a-Chip Experiment

After multivariate statistical analysis one representative batch each of the pre-fermentation and post-fermentation Yaomu samples showing the greatest differences was selected for subsequent LOC experiments. The Yaomu extracts were prepared according to the procedure described in Section 4.4, and the resulting extracts were concentrated under reduced pressure until no residual methanol odor was detectable. The concentrated unfermented Yaomu (WFJ) and fermented Yaomu (YM) samples were then subjected to freeze-drying for subsequent organ-on-a-chip experiments. The yields of WFJ and YM were 19.68% and 6.71%, respectively. Since the yields of YM and WFJ differ significantly, all subsequent experiments will be uniformly converted into crude drug concentration for analysis.

#### 4.6.2. Cytotoxicity Assay

THLE-2 cells in the logarithmic growth phase were harvested by trypsinization and resuspended to obtain a single-cell suspension. The cell suspension was transferred into centrifuge tubes and centrifuged at 1000 rpm for 5 min, after which the supernatant was discarded. Complete culture medium was added to the tubes, and the cells were gently resuspended by pipetting to obtain a uniform cell suspension for cell counting. Cells were seeded into 96-well plates at a density of 8000 cells per well, with three replicates for each group, and incubated at 37 °C in a humidified atmosphere containing 5% CO_2_ for 24 h. After cell attachment, the culture medium was aspirated and replaced with medium containing the prepared drug solutions at different concentrations, followed by incubation for an additional 24 h. After treatment, the drug-containing medium was removed, and blank culture medium supplemented with 10% CCK-8 reagent was added to each well. The plates were incubated for 40 min in the incubator, and absorbance was measured using a microplate reader to determine the appropriate concentrations for subsequent treatments.

#### 4.6.3. 3D Cell Culture in the Liver-on-a-Chip Mode

One day prior to cell seeding, the organ-on-a-chip devices were coated with Matrigel diluted in serum-free medium and incubated overnight in a cell culture incubator. The THLE-2 cell line was maintained in its specific complete medium at 37 °C in a humidified atmosphere containing 5% CO_2_. THLE-2 cells in the logarithmic growth phase were digested with 0.25% trypsin and diluted with complete medium to a final cell density of 1 × 10^7^ cells/mL. Subsequently, 4 μL of the cell suspension was carefully injected into the pre-coated microchannels of the chip. The chips were then placed in the incubator and kept static to allow cell attachment. After cell adhesion was achieved, culture medium was added dropwise to both ends of the channel to prevent evaporation of the medium within the channel. The culture medium was replaced daily.

#### 4.6.4. Perfusion

Set up four groups: a static group, a dynamic group, a YM group, and a WFJ group. Place the chips in an incubator and perform perfusion culture at a flow rate of 2 μL/min using a microfluidic syringe pump for 12 h. Subsequently, perfuse the drug solution diluted with the culture medium at a flow rate of 1 μL/min. After 12 h of perfusion, collect the chips and the effluent from the chips for subsequent analysis.

#### 4.6.5. Validation of Drug Hepatotoxicity Test

To verify the feasibility of drug toxicity testing on the chip, APAP was used to verify liver toxicity on the chip. After 12 h of perfusion culture, culture media containing different concentrations of APAP (0, 0.5, 1, 2.5, 5 mM) were added. After 12 h of perfusion, the supernatant was removed, and the cells were washed three times with PBS. The Calcein AM/PI double-staining kit was used to detect the survival/death status of THLE-2 cells, and the morphology of the cells was observed using a fully motorized inverted fluorescence microscope.

#### 4.6.6. Immunofluorescence

After completion of perfusion drug administration, the samples were fixed with methanol for 20 min and washed three times with PBS. Cell permeabilization was performed by incubation with Triton™ X-100 for 10 min at room temperature, followed by three washes with PBS. The samples were then blocked with QuickBlock™ Blocking Buffer for Immunofluorescence Staining for 10 min. The primary antibody was injected into the chip channels and incubated at 4 °C overnight. On the following day, the samples were washed three times with PBS, and a Cy3-conjugated goat anti-rabbit IgG (H+L), cross-adsorbed secondary antibody diluted 1:500 in immunofluorescence secondary antibody dilution buffer was added. After incubation at 4 °C for 1 h in the dark, the samples were washed three times with PBS. Subsequently, the samples were counterstained with DAPI for 5 min, washed three times with PBS, and finally imaged using a fully automated inverted fluorescence microscope. Image analysis was performed using ImageJ software (version 1.54p, National Institutes of Health (NIH), Bethesda, MD, USA).

#### 4.6.7. Measurement of Albumin, ALT and AST

After the drug administration and perfusion are completed, collect the culture medium flowing out of the chips in each group. Albumin levels in the liver-on-a-chip culture supernatants were determined using an ELISA kit. Samples were diluted according to the manufacturer’s instructions and incubated in antibody-coated 96-well plates at 37 °C, followed by incubation with enzyme conjugate and TMB substrate. After stopping the reaction, absorbance was measured at 450 nm, and albumin concentrations were calculated from the standard curve. ALT and AST activities were measured using commercial biochemical assay kits according to the manufacturer’s protocols. Measurements were conducted using a multifunctional microplate reader, and the results were expressed as U/L.

#### 4.6.8. Measurement of Intracellular ROS and ΔΨm

After stimulating the liver-on-a-chip with different concentrations of WFJ (7.8 mg/mL) and YM (7.8 mg/mL) for 12 h, they were incubated with DCFH-DA diluted 1000-fold at 37 °C for 20 min to determine the presence of ROS. Mitochondrial membrane potential was evaluated using the JC-1 probe. Cells were washed with PBS, incubated with 1× JC-1 working solution at 37 °C for 20 min, and washed three times with JC-1 buffer. Fluorescence was recorded using a fluorescence microscope. The red/green fluorescence ratio was calculated to assess mitochondrial functional status.

#### 4.6.9. Detection of Cell Apoptosis

To specifically evaluate apoptosis in the liver microfluidic chip induced by fermented and unfermented Yaomu extracts, immunofluorescence staining for cleaved caspase-3 was performed. The fluorescence intensity and the proportion of cleaved caspase-3-positive cells were analyzed to assess apoptotic responses induced by fermented and unfermented Yaomu under dynamic perfusion conditions. In addition, after extracts of WFJ (7.8 mg/mL) and YM (7.8 mg/mL) treatment for 12 h, apoptosis was detected with the calcein-AM/PI double-staining kit. The fluorescent probe incubated the cells in darkness at 37 °C for 20 min. The image was analyzed using ImageJ software.

#### 4.6.10. Liver-on-a-Chip Data Analysis

Fluorescence intensity was semi-quantitatively analyzed using ImageJ software (National Institutes of Health, Bethesda, MD, USA). The statistical analysis was performed in GraphPad Prism 9.1.0 (GraphPad Software, LLC, Boston, MA, USA). All data are presented as mean ± standard deviation (SD). Differences among multiple groups were analyzed using one-way analysis of variance (ANOVA), followed by appropriate post hoc multiple comparison tests when applicable.

## 5. Conclusions

In summary, the integrated qualitative and quantitative analyses revealed that fermentation was associated with substantial remodeling of the chemical composition of Yaomu, accompanied by decreased levels of several alkaloid subclasses previously linked to toxicity. The in vitro organ-on-a-chip platform provided a physiologically relevant system for evaluating hepatocellular responses under a controlled microenvironment and enabled characterization of cellular injury patterns and functional alterations. Within this model, extracts obtained after fermentation were associated with attenuated hepatocellular stress, as reflected by improved cell viability, preserved metabolic activity, and lower levels of liver injury-related biomarkers compared with unfermented materials. Collectively, these observations suggest that fermentation as a traditional processing strategy may contribute to reduced hepatotoxic potential of Yaomu, although a direct causal relationship between specific chemical transformations and toxicity outcomes cannot be conclusively established in the present study. The combined application of high-resolution mass spectrometry and organ-on-a-chip technology provides an integrative framework for toxicity evaluation and offers translationally relevant evidence for safety assessment of complex traditional medicines undergoing fermentation-based processing. Future studies integrating mechanistic validation and in vivo investigations will be required to further clarify the relationships between fermentation-induced metabolic changes and toxic effects.

## Data Availability

No new data were created or analyzed in this study. Data sharing is not applicable to this article.

## Supplementary Materials

The following supporting information can be downloaded at: https://www.mdpi.com/article/10.3390/molecules31060994/s1, Figure S1. Total ion chromatograms (TICs) of Yaomu samples analyzed in positive and ion mode using UHPLC-Q-TOF-MS. (a) unfermented samples in positive ion; (b) unfermented samples in negative ion; (c) fermented samples in positive ion; (d) fermented samples in negative ion. Figure S2. Typical mass spectra of 9 compounds and 2 ISs in standard substances. (a) Standard sample; (b) unfermented Yaomu samples; (c) fermented Yaomu samples; (d) blank matrices. Table S1. Mass data of unfermented Yaomu sample in negative ion. Table S2. Mass data of fermented Yaomu sample in negative ion. Table S3. Mass data of unfermented Yaomu sample in positive ion. Table S4. Mass data of fermented Yaomu sample in positive ion. Table S5. Significantly different compounds up- and down-regulated in unfermented Yaomu vs fermented Yaomu. Table S6. MRM parameters of 9 compounds and 2 ISs. Table S7. The calibration curves, linearity range, and LOQs, LODs of 9 compounds (*n* = 6). Table S8. Precision, accuracy, and stability for 9 compounds. Table S9. Repeatability for 9 compounds (*n* = 6). Table S10. Extraction recovery for 9 compounds (*n* = 3). Table S11. Content of 9 components in the YM before and after fermentation (*n* = 6, mean ± SD).

## Abbreviations

The following abbreviations are used in this manuscript:

HFD	Huafeng Dan
LPCs	Lysophosphatidylcholines
LPC	Lysophosphatidylcholine
TCM	Traditional Chinese medicine
LOC	Lab-on-a-chip
WFJ	Unfermented Yaomu
YM	Fermented Yaomu
PCA	Principal Component Analysis
OPLS-DA	Orthogonal partial least squares discriminant analysis
VIP	Variable importance in projection
AC	Aconitine
HA	Hypaconitine
MA	Mesaconitine
BHA	Benzoylhypaconitine
BMA	Benzoylmesaconitine
ACN	Aconine
HAN	Hypaconine
LPC (18:0)	1-Stearoyl-2-Hydroxysn-Glycero-3-Phosphatidylcholine
LPC (18:1)	1-oleoyl-2-hydroxy -sn-glycero-3-phosphocholine
BBR	Berberine
MF	Miltefosine
APAP	Acetaminophen
CYP3A4	Cytochrome p450 3A4
DAPI	4′,6-Diamidino-2-Phenylindole
ALT	Alanine Aminotransferase
AST	Aspartate Aminotransferase
ROS	Reactive Oxygen Species
ΔΨm	Mitochondrial Membrane Potential
DCFH-DA	2′,7′-Dichlorodihydrofluorescein diacetate
