# Supplementary material for: Integrated UHPLC-Q-TOF/MS and Liver-on-a-Chip Evaluation of Chemical Composition Changes and Hepatotoxicity Differences in Yaomu Before and After Fermentation
Source: Molecules. 2026 Mar 16;31(6):994. doi: 10.3390/molecules31060994 (PMC13029428; doi:10.3390/molecules31060994)
Supplement: Supplementary file 1 [file molecules-31-00994-s001.zip › molecules-4159593-supplementary.pdf]

Integrated UHPLC-Q-TOF/MS and Liver-on-a-Chip Evaluation of Chemical Composition Changes and Hepatotoxicity Differences in Yaomu Before and After Fermentation

Kexin Ma [1,2,3,#], Lijun An[3,#], Guo Feng[1,2,*], Wei Li[1,2], Tingting Liu[1], Jinxin Hou[1], Ping Wang[3], Meifang Li[3], Bing Wang[3,*], Xie-an Yu[3,*]

[1] Department of Chinese Materia Medica, Guizhou University of Traditional Chinese Medicine, Guiyang 550025, China
[2] Guizhou Inheritance Base of Traditional Chinese Medicine Processing Technology, Guizhou, Guiyang 550025, China
[3] NMPA Center for Innovation and Research in Regulatory Science，Shenzhen Institute for Drug Control, Shenzhen China, 518057
* Correspondence: Guo Feng：453989352@qq.com; Bing Wang: wangbingszyj@163.com; Xie-an Yu: yuxieanalj@126.com.

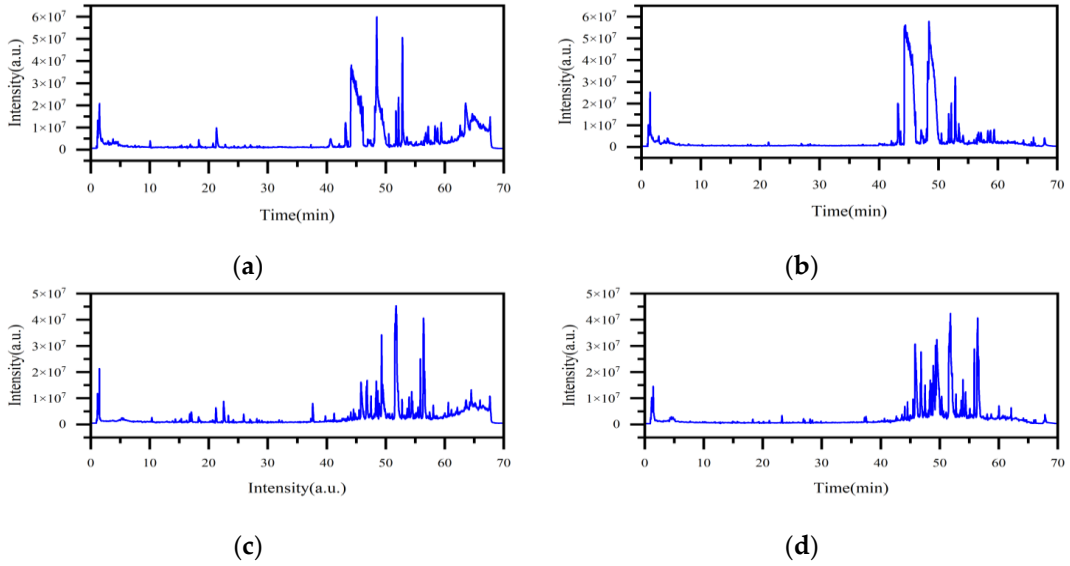

Figure S1. Total ion chromatograms (TICs) of Yaomu samples analyzed in positive and ion mode using UHPLC-Q-TOF-MS. (**a**) unfermented samples in positive ion; (**b**) unfermented samples in negative ion; (**c**) fermented samples in positive ion; (**d**) fermented samples in negative ion.

Table S1. Mass data of unfermented Yaomu sample in negative ion.

Peak No.	RT (min)	Formula	iron	Mass (m/z)	Fragment Ions (m/z)	Identification	ppm	Confidence Level
1	48.49	$C_{26}H_{43}NO_6$	M-H	464.3017	464.3022,428.2297,74.0248	Glycocholic acid	-0.1	Level 2
2	57.00	$C_{26}H_{43}NO_6$	M-H	464.3013	464.3032,420.3124,402.3019,74.0245	Isomer of Glycocholic acid	-1.0	Level 3
3	52.17	$C_{26}H_{43}NO_5$	M-H	448.3067	448.3077,386.3069,74.0248	Glycohyodeoxycholic acid	2.1	Level 2
4	48.51	$C_{26}H_{41}NO_5$	M-H	446.2915	446.2919,402.3037,348.2914,74.0245	6-keto-glycohyodeoxycholic acid	0.7	Level 2
5	47.21	$C_{26}H_{43}NO_5$	M-H	448.3068	448.3064,386.3052,74.0252	Glycodeoxycholic acid	0.1	Level 2
6	51.73	$C_{24}H_{40}O_5$	M-H	407.2803	407.2815,345.2806,289.2182	Hyocholic acid	0.3	Level 2
7	46.87	$C_{24}H_{40}O_5$	M-H	407.2803	407.2808,389.2701,371.2601,345.2795,343.2639	Cholic acid	1.5	Level 2
8	50.00	$C_{26}H_{43}NO_5$	M-H	448.3067	448.3065,386.3069,74.0246	Glycochenodeoxycholic acid	-0.3	Level 2
9	50.53	$C_{26}H_{41}NO_5$	M-H	446.2912	446.2919,402.3028,348.2910,74.0249	7-keto-glycochenodeoxycholic acid	-0.9	Level 3
10	56.40	$C_{24}H_{40}O_4$	M-H	391.2850	391.2914,355.2648,345.2798,327.2695	Chenodeoxycholic acid	-1.0	Level 2
11	55.92	$C_{24}H_{40}O_4$	M-H	391.2852	391.2847,355.2644,345.2796,327.2693	Hyodeoxycholic acid	-0.5	Level 2
12	56.45	$C_{24}H_{40}O_4$	M-H	391.2854	391.2845,355.2645,345.2798,327.2693	Deoxycholic acid	-1.0	Level 2
13	28.47	$C_{26}H_{28}O_{14}$	M-H	563.1409	353.0661,383.0766,413.0811,443.1007	Schaftoside	0.5	Level 2
14	19.58	$C_{11}H_{12}O_6$	M-H	239.0561	96.965,170.88785	(3R,4S)-4,6,8-trihydroxy-7-methoxy-3-methyl-3,4-dihydroisochromen-1-one	1.6	Level 2

15	6.35	$C_{10}H_{13}N_5O_5$	M-H	282.0848	108.019 29,108.598 31,133.014 6,136.822 36,150.041 66	Guanosine	1.4	Level 2
16	4.57	$C_5H_4N_4O_2$	M-H	151.0261	108.02159	Xanthine	2.3	Level 2
17	5.44	$C_9H_{11}NO_3$	M-H	180.0660	93.0401 9,93.045 75,119.056 9,119.059 41,121.494 9,163.043 17	Tyrosine	1.0	Level 2
18	25.85	$C_{10}H_{13}NO_3$	M-H	194.0823	179.05382	N-Methyltyrosine	-1.4	Level 2
19	28.08	$C_{26}H_{28}O_{14}$	M-H	563.1406	293.043 79,413.081 12	Isovitexin-2''-O-arabinoside	-0.1	Level 2
20	4.69	$C_9H_{12}N_2O_6$	M-H	243.0623	66. 041 35,82.033 26,110.025 82,111.022 42,122.028 31,140.036 53,152.034 04,200.051 94	Uracil arabinofuranoside	0.2	Level 2
21	1.30	$C_5H_7NO_3$	M-H	128.0353	109,89	Pyroglutamic acid	0.7	Level 2
22	18.98	$C_{15}H_{22}O_5$	M-H	281.1394	263,237,219,191	Zedoalactone B	1.3	Level 2
23	53.11	$C_{15}H_{20}O_3$	M-H	247.1340	197,187,161,153,119	Codonolactone	-1.5	Level 2
24	54.16	$C_{20}H_{32}O_3$	M-H	319.2279	319,273,233,219,187	Curcuminol A	-3.0	Level 2
25	65.09	$C_{19}H_{30}O_2$	M-H	289.2173	267,233,197,161,113	Curcuminol E	-2.1	Level 2
26	25.27	$C_{26}H_{28}O_{14}$	M-H	563.1406	545,503,473	Apigenin-6-C-β-D-xylopyranosyl-8-C-α-L-arabinopyranoside	1.0	Level 2
27	1.46	$C_{12}H_{22}O_{11}$	M-H	341.1089	113.0246,101.0243,89.0244,71.0140,59.0138	Lactulose	2.2	Level 2
28	1.53	$C_6H_8O_7$	M-H	191.0197	191.0535,129.0178,111.0087,87.0084,85.0297	Citric acid	2.5	Level 2
29	10.21	$C_{13}H_{16}O_9$	M-H	315.0722	153.0118,109.0287	Protocatechuic acid 4-O-β-glucoside	0.1	Level 2

30	24.13	$C_8H_8O_4$	M-H	167.0350	108.0207,123.0441	Vanillic acid	0.7	Level 2
31	14.83	$C_9H_8O_4$	M-H	179.0350	135.0454,107.0520	Caffeic acid	1.8	Level 2
32	10.67	$C_{11}H_{12}O_7$	M-H	255.0514	195.0296,149.0610,123.0450	Hydroxyleucine	1.5	Level 2
33	15.80	$C_9H_8O_3$	M-H	163.0403	119.0511	P-coumaric acid	1.4	Level 2
34	10.39	$C_7H_6O_4$	M-H	153.0193	109.0291	Protocatechuic acid	1.7	Level 2
35	31.51	$C_{21}H_{20}O_{10}$	M-H	431.0984	269.0505,268.0530,311.0550	Apigenin-7-O-glucoside	-0.9	Level 2
36	30.61	$C_{21}H_{20}O_{11}$	M-H	447.0933	285.0390,151.0048,107.0151	Cynaroside	0.3	Level 2
37	8.37	$C_7H_6O_3$	M-H	137.0247	75.0237,93.0344	Protocatechuic aldehyde	2.1	Level 2

Table S2. Mass data of fermented Yaomu sample in negative ion.

Peak No.	RT (min)	Formula	iron	Mass (m/z)	Fragment Ions (m/z)	Identification	ppm	Confidence Level
1	48.4	$C_{26}H_{43}NO_6$	M-H	464.3018	464.3022,428.2297,74.0248	Glycocholic acid	2.5	Level 2
2	44.47	$C_{26}H_{43}NO_6$	M-H	464.3018	464.3032,420.3124,402.3019,74.0245	Isomer of Glycocholic acid	3.7	Level 3
3	52.64	$C_{26}H_{43}NO_5$	M-H	448.3068	448.3077,386.3069,74.0248	Glycohyodeoxycholic acid	2.3	Level 2
4	50.52	$C_{26}H_{41}NO_5$	M-H	446.2912	446.2919,402.3037,348.2914,74.0245	6-keto-glycohyodeoxycholic acid	3.1	Level 3
5	47.15	$C_{26}H_{43}NO_5$	M-H	448.3068	448.3064,386.3052,74.0252	Glycodeoxycholic acid	3.2	Level 2
6	46.87	$C_{24}H_{40}O_5$	M-H	407.2815	407.2815,345.2806,289.2182	Hyocholic acid	3.0	Level 2
7	51.88	$C_{24}H_{40}O_5$	M-H	407.2803	407.2808,389.2701,371.2601,345.2795,343.2639	Cholic acid	2.2	Level 2
8	48.46	$C_{26}H_{43}NO_5$	M-H	448.3068	448.3065,386.3069,74.0246	Glycochenodeoxycholic acid	3.2	Level 2
9	52.76	C26H41NO5	M-H	446.2912	446.2919,402.3028,348.2910,74.0249	7-keto-glycochenodeoxycholic acid	2.0	Level 3
10	53.97	$C_{24}H_{40}O_4$	M-H	391.2854	391.2914,355.2648,345.2798,327.2695	Chenodeoxycholic acid	2.6	Level 2
11	56.37	$C_{24}H_{40}O_4$	M-H	391.2863	391.2847,355.2644,345.2796,327.2693	Hyodeoxycholic acid	2.3	Level 2
12	55.90	$C_{24}H_{40}O_4$	M-H	391.2854	391.2845,355.2645,345.2798,327.2693	Deoxycholic acid	3.6	Level 2
13	27.05	$C_{26}H_{28}O_{14}$	M-H	563.1431	353.066 1,383.076 6,413.081 1,443.100 7	Schaftoside	4.4	Level 2

14	19.46	$C_{11}H_{12}O_6$	M-H	239.0561	96.965,170.88785	(3R,4S)-4,6,8-trihydroxy-7-methoxy-3-methyl-3,4-dihydroisochromen-1-one	3.3	Level 2
15	57.61	$C_{30}H_{46}O_4$	M-H	469.3323	425.33066	18β-Hydroxyglycyrrhetinic acid	1.4	Level 3
16	6.62	$C_{10}H_{13}N_5O_5$	M-H	282.0844	108.019 29,108.598 31,133.014 6 ,136.822 36,150.041 66	Guanosine	2.2	Level 2
17	4.90	$C_5H_4N_4O_2$	M-H	151.0261	108.02159	Xanthine	2.3	Level 2
18	5.12	$C_9H_{11}NO_3$	M-H	180.0675	93.040 19,93.045 75,119.056 9,119.059 41,12 .494 9,163.043 17	Tyrosine	4.9	Level 2
19	13.58	$C_{10}H_{13}NO_3$	M-H	194.0823	179.05382	N-Methyltyrosine	2.7	Level 2
20	27.05	$C_{26}H_{28}O_{14}$	M-H	563.1406	293.043 79,413.081 12	Isovitexin-2''-O-arabinoside	4.4	Level 2
21	5.14	$C_9H_{12}N_2O_6$	M-H	243.0623	66.041 35,82.033 26,110.025 82,111.022 42,122.028 31,140.036 53,152.034 04,200.051 94	Uracil arabinofuranoside	2.6	Level 2
22	3.19	$C_5H_7NO_3$	M-H	128.0353	109,89	Pyroglutamic acid	2.2	Level 2
23	14.46	$C_{15}H_{22}O_5$	M-H	281.1404	263,237,219,191	Zedoalactone B	3.4	Level 2
24	48.56	$C_{22}H_{36}O_5$	M-H	379.2490	361,347,343,241,197	Curcuminol C	0.8	Level 2
25	48.30	$C_{15}H_{24}O_3$	M-H	251.1653	233,219,207,197,187,161,119	(*1S,4S,5S,10R*)-Zedoaronediol	4.1	Level 3
26	53.10	$C_{15}H_{20}O_3$	M-H	247.1340	197,187,161,153,119,000	Codonolactone	1.3	Level 2
27	54.08	$C_{20}H_{32}O_3$	M-H	319.2279	319,273,233,219,187,000	Curcuminol A	0.1	Level 2
28	65.05	$C_{19}H_{30}O_2$	M-H	289.2173	267,233,197,161,113,000	Curcuminol E	1.4	Level 2
29	25.13	$C_{26}H_{28}O_{14}$	M-H	563.1441	545,503,473	Apigenin-6-C-arabinose-8-C-galactoside	4.4	Level 3

30	3.79	$C_6H_8O_7$	M-H	191.0197	191.0535,129.0178,111.0087,87.0084,85.0297	Citric acid	4.1	Level 2
31	28.99	$C_8H_8O_4$	M-H	167.0350	108.0207,123.0441	Vanillic acid	4.3	Level 2
32	13.02	$C_{11}H_{12}N_2O_2$	M-H	203.0826	159.0880,116.0508	DL-tryptophan	3.9	Level 2
33	14.48	$C_9H_8O_4$	M-H	179.0350	135.0454,107.0520	Caffeic acid	4.6	Level 2
34	10.29	$C_{11}H_{12}O_7$	M-H	255.0519	195.0296,149.0610,123.0450	Hydroxyleucine	3.4	Level 2
35	15.67	$C_9H_8O_3$	M-H	163.0401	119.0511	P-coumaric acid	3.3	Level 2
36	35.40	$C_{16}H_{18}O_9$	M-H	353.0878	191.0560,135.0450,179.0347	Neochlorogenic acid	4.8	Level 2
37	10.17	$C_7H_6O_4$	M-H	153.0199	109.0291	Protocatechuic acid	3.7	Level 2
38	31.50	$C_{21}H_{20}O_{10}$	M-H	431.1005	269.0505,268.0530,311.0550	Apigenin-7-O-glucoside	4.9	Level 2
39	28.65	$C_{21}H_{20}O_{11}$	M-H	447.0938	285.0390,151.0048,107.0151	Cynaroside	1.2	Level 2
40	13.83	$C_7H_6O_3$	M-H	137.0244	75.0237,93.0344	Protocatechuic aldehyde	3.5	Level 2

Table S3. Mass data of unfermented Yaomu sample in positive ion.

Peak No.	RT (min)	Formula	iron	Mass (m/z)	Fragment Ions (m/z)	Identification	ppm	Confidence Level
1	1.36	$C_5H_{11}NO_2$	M+H	118.0871	118.0860,58.0645	Valine	4.6	Level 2
2	4.52	$C_6H_{13}NO_2$	M+H	132.1025	132.1121,116.0576,86.0962	Isoleucine	4.5	Level 2
3	2.84	$C_5H_5N_5$	M+H	136.0623	136.0614,119.0349,92.0240	Adenine	3.9	Level 2
4	3.95	$C_5H_4N_4O$	M+H	137.0461	137.0641,119.0348,110.0351	Hypoxanthine	2.3	Level 2
5	4.61	$C_9H_{12}N_2O_6$	M+H	245.0680	130.9670,110.0251	Uridine	4.8	Level 2
6	7.52	C_8H_9N	M+H	120.0811	120.0804,103.0540,77.0382	N-Benzylidene(methyl)amine	2.7	Level 3
7	9.68	$C_{10}H_{13}N_5O_5$	M+H	284.1440	150.0421,133.0156,108.0202	Guanosine	-3.1	Level 2
8	5.63	$C_{10}H_{13}N_5O_4$	M+H	268.1048	136.0612,119.0351	Adenosine	2.9	Level 2
9	4.62	$C_9H_{10}O_2$	M+H	151.0754	91.0534,77.0363,	3-Phenylpropionic acid	4.9	Level 2
10	4.4	$C_5H_4N_4O_2$	M+H	153.0411	153.1245,136.0135,110.0353	2,6-Dihydroxypurine	2.6	Level 2
11	5.31	$C_{23}H_{37}NO_9$	M+H	472.2557	472.2563,440.2257,422.2173,404.2089,58.0647	Beiwutinine	3.4	Level 2
12	13.94	$C_{23}H_{37}NO_7$	M+H	440.2642	440.2643,422.2549,390.2273,58.0647	Isomer of 9-Hydroxysenbusine A	-0.2	Level 3
13	16.31	$C_{23}H_{37}NO_6$	M+H	424.2691	424.2698,406.2581,374.2336,58.0642	Isomer of Senbusine A	-0.6	Level 3
14	15.39	$C_{24}H_{39}NO_9$	M+H	486.2708	486.2703,454.2648,436.2327,404.2075,58.0641	Isomer of Mesaconine	0.5	Level 3
15	13.15	$C_{11}H_{12}N_2O_2$	M+H	205.0980	188.0712,146.0600	Tryptophan	4.1	Level 2
16	13.10	$C_{11}H_9NO_2$	M+H	188.0709	188.0401,170.0606,142.0652,11	3-Amino-2-naphthoic acid	1,6	Level 2

					5.0549			
17	14.81	$C_{22}H_{35}NO_6$	M+H	410.2550	410.2550,378.2559,328.1939,58.0646	Isomer of N-Deethylaconine	3.1	Level 3
18	15.11	$C_{21}H_{33}NO_4$	M+H	364.2477	364.2480,346.2381,328.2282,58.0649	16-Hydroxycardiopetaline	-1.5	Level 3
19	15.39	$C_{24}H_{39}NO_9$	M+H	486.2670	486.2673,454.2435,436.2318,404.2068,155.0852	Mesaconine	0.5	Level 1
20	19.08	$C_{15}H_{29}NO_4$	M+H	288.2177	288.2162,270.2058,210.1853	N-Lauroyl-L-Serine	2.7	Level 2
21	16.26	$C_{16}H_{17}NO_3$	M+H	272.1286	255.1021,237.0914,161.0599,107.0489	Higenamine	1.8	Level 2
22	14.94	$C_{22}H_{33}NO_4$	M+H	376.2484	376.2491,358.2385,326.2127,58.0650	Beiwusine A	0.4	Level 2
23	15.83	$C_{24}H_{39}NO_7$	M+H	454.2813	454.2805,436.2708,404.2431,58.0657	Senbusine C	3.0	Level 2
24	16.88	$C_{22}H_{35}NO_4$	M+H	378.2641	378.2616 360.2516,342.2430,58.0646	Karakoline	0.6	Level 2
25	17.21	$C_{23}H_{37}NO_5$	M+H	408.2745	408.2719 390.2610,358.2367,58.0644	Talatisidine	0.1	Level 2
26	9.98	$C_{23}H_{37}NO_7$	M+H	440.2653	440.26503,422.2574,408.2402,390.2308,58.0646	9-Hydroxysenbusine A	2.3	Level 2
27	17.41	$C_{23}H_{37}NO_6$	M+H	424.2707	424.2703,392.2438,374.2333,58.0646	Senbusine A	3.1	Level 2
28	18.51	$C_{25}H_{41}NO_9$	M+H	500.2862	500.2830,468.2598,450.2480,418.2230,58.0646	Aconine	1.6	Level 1
29	24.30	$C_{24}H_{39}NO_6$	M+H	438.2858	438.2844,406.2591,388.2491,58.	6-epi-Forsticine	1.8	Level 3

					0652			
30	20.51	$C_{25}H_{39}NO_7$	M+H	466.2799	448.2686,416.2452,58.0644	Isomer of Delbruine	4.0	Level 3
31	18.22	$C_{25}H_{41}NO_8$	M+H	484.2919	484.28897,466.2773,434.2557,58.0637	Deoxyaconine	2.9	Level 2
32	3.11	$C_{11}H_{19}NO_6$	M+H	262.1290	262.1368,202.0873,184.0747	N-(4-methyl-2-pentanoyl)-glutamic acid	1.9	Level 3
33	23.35	$C_{25}H_{39}NO_7$	M+H	466.2799	466.2816,448.2687,416.2443,58.0652	Delbruine	2.1	Level 2
34	6.97	$C_{22}H_{35}NO_6$	M+H	410.2548	410.2512,378.2559,360.2185,105.076058.0643	N-Deethylaconine	2.6	Level 2
35	21.32	$C_{23}H_{35}NO_6$	M+H	422.2556	422.2544,404.2444,372.2199,58.0654	Gadesine	4.5	Level 2
36	56.02	$C_{15}H_{16}O_2$	M+H	229.1226	229.1232 201.1252,187.0770	Curcumin A	1.3	Level 2
37	13.50	$C_{24}H_{37}NO_6$	M+H	436.2709	436.2318,418.2222,386.1962,58.0647	Guiwuline	3.5	Level 2
38	21.08	$C_{24}H_{39}NO_8$	M+H	470.2753	470.2725,438.2472,406.2224	Hypaconine	1.0	Level 1
39	13.17	$C_{11}H_9NO_2$	M+H	188.0709	170.0951,146.0597,118.0659	6-Amino-2-naphthoic acid	1.6	Level 2
40	24.06	$C_{25}H_{39}NO_6$	M+H	450.2850	450.2835,418.2607,400.2433,58.0645	Isomer of Condelphine	0.0	Level 3
41	10.09	$C_{24}H_{33}NO_5$	M+H	416.2422	416.2440,330.2075,312.1972,103.0387	Guanfu base Z	-2.3	Level 2
42	21.30	$C_{24}H_{37}NO_6$	M+H	436.2703	436.2708,418.1618,404.2449,58.0657	Isomer of Guiwuline	2.1	Level 3
43	21.31	$C_{24}H_{39}NO_7$	M+H	454.2789	454.2775,436.2691,404.2433,58.0647	Fuziline	-2.3	Level 2

44	22.56	$C_{23}H_{37}NO_5$	M+H	408.2757	408.2748,390.2647,376.2502,58.0650	Isotaatizidine	3.1	Level 3
45	46.71	$C_{15}H_{20}O_4$	M+H	265.1440	247.1332,229.1219,201.1272	Zedoarofuran	2.1	Level 2
46	18.37	$C_{24}H_{37}NO_8$	M+H	468.2597	468.2945,436.2326,418.2596,386.232658.0646	1,7,8-trihydroxy-16-methoxy-4-(methoxymethyl) aconitane-14-ylacetate	1.1	Level 3
47	19.87	$C_{24}H_{39}NO_7$	M+H	454.2803	422.2520,404.2433,58.0647	Isomer of Fuziline	0.8	Level 3
48	28.72	$C_{25}H_{41}NO_7$	M+H	468.2963	468.2976,436.2697,404.2454,58.0640	Isomer of Lycoctonin	1.5	Level 3
49	24.94	$C_{26}H_{41}NO_7$	M+H	480.2964	480.2969,448.2692,430.2618,58.0663	Bullatine C	1.7	Level 2
50	18.69	$C_{22}H_{33}NO_3$	M+H	360.2533	360.2538,342.2432,298.2155,58.0642	Spiramine H	0.5	Level 2
51	29.17	$C_{23}H_{33}NO_6$	M+H	452.3008	420.2748,388.2489,356.2229,58.0645	Isomer of Giraldine F	1.2	Level 3
52	28.28	$C_{25}H_{41}NO_6$	M+H	452.3020	452.3009,420.2750,388.2490,58.0650	Chasmanine	3.0	Level 2
53	25.58	$C_{23}H_{37}NO_4$	M+H	392.2802	392.2785,360.2528,342.2428,58.0646	Sachaconitine	1.7	Level 2
54	27.06	$C_{26}H_{28}O_{14}$	M+H	565.1554	563.1402,353.0659	Apigenin-6-C-β-D-Galactose-8-C-α-L-arabinopyranoside	0.4	Level 3
55	14.52	$C_{22}H_{33}NO_5$	M+H	392.2437	392.2783,360.2530,342.2436,58.0648	Heteratisine or Pengshenine A	1.4	Level 3
56	25.59	$C_{25}H_{39}NO_6$	M+H	450.2850	450.2534,418.2597,400.2501,58.0647	Condelphine	4.2	Level 2
57	28.67	$C_{23}H_{33}NO_6$	M+H	420.2394	420.2751,388.2497,356.2219,58.	Giraldine F	3.2	Level 2

					0647			
58	22.99	$C_{24}H_{39}NO_6$	M+H	438.2861	438.282,406.2572,388.2479,58.0645	Neoline	2.5	Level 2
59	27.06	$C_{15}H_{29}NO_3$	M+H	272.2220	254.2117,81.0693	Tridecanoylglycine	2.0	Level 2
60	22.22	$C_{25}H_{41}NO_7$	M+H	468.2972	468.2954,436.2697,404.2454,58.0646	Lycoctonine	3.5	Level 2
61	24.92	$C_{26}H_{41}NO_7$	M+H	480.2970	480.2964,448.2701,430.2595,58.0646	Isomer of Bullatine C	3.0	Level 3
62	17.76	$C_{27}H_{43}NO_8$	M+H	510.3061	510.3079,492.2967,460.2708,58.0658	14-O-Acetylbrowniine	3.2	Level 2
63	29.90	$C_{15}H_{24}O_3$	M+H	253.1808	253.1807,235.1696,177.1274,159.1168	Zedoarondiol	3.9	Level 2
64	41.18	$C_{31}H_{43}NO_9$	M+H	574.3014	574.2655,542.2393,510.2133,105.0334	Isomer of Benzoylhypacoitine	0.6	Level 3
65	49.31	$C_{15}H_{18}O_2$	M+H	231.1385	231.1114,203.0815	Epicurzerenone	2.4	Level 2
66	46.12	$C_{26}H_{41}NO_6$	M+H	464.3002	464.2967,432.2730,414.2651,58.0646	Monoacetyltalatisamine	-1.0	Level 2
67	36.19	$C_{31}H_{43}NO_{11}$	M+H	606.2908	556.2565,524.2301,105.0326	(-) - (A-b)-14α-benzoyloxy-3α,10β,13β,15α-pentahydroxy-1α,6α,16β,18-tetramethoxy-N-methylaconitane	-0.1	Level 3
68	31.70	$C_{15}H_{20}O_4$	M+H	265.1447	265.0971,247.0868,219.0919	Curcumenolactone C	4.8	Level 2
69	18.43	$C_{19}H_{20}O$	M+H	265.1576	265.0975,247.0874,219.1251	trans, trans-1,7-diphenyl-1,3-heptadien-5-ol	-4.1	Level 3

70	56.78	$C_{15}H_{18}O$	M+H	215.1438	215.1429,197.1335,187.1481,167.0851	(+)-agassizin	3.5	Level 2
71	63.90	$C_{22}H_{37}NO_3$	M+H	364.2842	364.2868,346.2746,328.2615,310.2540	N-(2-hydroxyethyl) eicosapentaenamide	-1.2	Level 3
72	45.49	$C_{31}H_{41}NO_8$	M+H	556.2918	556.2554,524.2267,492.2075,105.0340	Dehydrated benzoylhypaconine	2.3	Level 2
73	40.62	$C_{31}H_{43}NO_{10}$	M+H	590.2960	590.2977,540.2570,508.2334,105.0323	Isomer of Benzoylmesaconine	0.0	Level 3
74	61.25	$C_8H_4O_3$	M+H	149.0239	121.0278,93.0327	Phthalic anhydride	3.9	Level 2
75	53.15	$C_{21}H_{37}NO$	M+H	320.2951	302.2582,287.8937	(2S)-2-(dodecylamino)-3-phenyl-1-propanol	1.0	Level 2
76	52.89	$C_{22}H_{37}NO_2$	M+H	348.2898	348.2748,330.2423,312.2335,	Arachidonylethanolamide	0.3	Level 2
77	40.62	$C_{31}H_{43}NO_{10}$	M+H	590.2960	590.2937,540.2570,508.2330,105.0326	Benzoylmesaconine	0.0	Level 1
78	39.21	$C_{31}H_{43}NO_9$	M+H	574.3024	574.3020,542.2758,524.2592,105.0339,58.0648	Benzoylhypaconine	2.0	Level 1
79	44.17	$C_{32}H_{45}NO_{10}$	M+H	604.3122	604.3090,572.2861,554.2745,522.2495,105.0330	Benzoylaconine	1.0	Level 2
80	41.02	$C_{30}H_{41}NO_7$	M+H	528.2961	528.2956,510.2857,496.2714,105.0336	6-Demethoxy Benzoylneoline	1.0	Level 3
81	51.51	$C_{15}H_{18}O_3$	M+H	247.1329	247.355,229.1243,201.1263,189.0910	Zederone	1.3	Level 2
82	48.38	$C_{26}H_{45}NO_6S$	M+H	500.3032	498.2845,454.2968,408.291,179.9591	Taurochenodeoxycholic acid	-1.7	Level 2
83	48.74	$C_{33}H_{45}NO_9$	M+H	600.3178	600.2822,540.2615,508.2691,48	Isomer of 13-Deoxyhypaconitine	1.8	Level 3

					0.2749,105.0327			
84	8.93	$C_{21}H_{33}NO_4$	M+H	364.2496	364.2854,346.2779,328.2626,276.2319	Isomer of 16β-Hydroxycardiopetaline	3.7	Level 3
85	44.58	$C_{32}H_{43}NO_9$	M+H	596.3004	586.3022,554.2763,536.2724,	1,15-dimethoxy-3-hydroxy-14-benzoyl-16-keto-neoline	-1.1	Level 3
86	56.78	$C_{15}H_{20}O_2$	M+H	233.1543	233.1532,215.1440,175.1123	Furanogermenone	3.0	Level 2
87	48.47	$C_{34}H_{47}NO_{11}$	M+H	646.3226	646.3233,586.3121,554.2711,105.0335	Aconitine	0.6	Level 1
88	37.68	$C_{15}H_{22}O_3$	M+H	251.1642	251.1627,233.1531,215.1442	Aerugidiol	4.9	Level 2
89	44.97	$C_{33}H_{45}NO_{12}$	M+H	648.3008	648.3013,588.2806,556.2553,105.0338	Beiwutine	-1.0	Level 2
90	45.49	$C_{31}H_{41}NO_8$	M+H	556.2905	556.2924,524.2660,506.2617,105.0327	Isomer of Dehydrated benzoylhypaconine	2.3	Level 3
91	43.20	$C_{30}H_{41}NO_7$	M+H	528.2971	528.2969,496.2714,478.2590,105.0344	(-) - (A-b)-14α-benzoyloxy-N-ethyl-1α,8β,15α-trihydroxy-16β,18-dimethoxyaconitane	2.9	Level 3
92	44.32	$C_{31}H_{43}NO_8$	M+H	558.3061	558.3041,526.2795,508.2707,105.0332	(-) - (A-b)-14α-benzoyloxy-N-ethyl-1α,8β,15α-trihydroxy-6α,16β,18-trimethoxyaconitane	3.5	Level 3
93	45.32	$C_{32}H_{45}NO_9$	M+H	588.3175	588.3145,556.2897,524.2657,105.0336	14-Benzoylpseudaconine	1.3	Level 2
94	52.78	$C_{15}H_{20}O$	M+H	217.1595	217.1599,199.1512,161.0945,105.0700	Comosone II	3.7	Level 2
95	45.78	$C_{33}H_{45}NO_{11}$	M+H	632.3050	632.3066,572.2866,540.2593,508.2358,105.0327	10-Hydroxy-hypaconitine	-2.4	Level 3

96	55.59	$C_{26}H_{48}NO_7P$	M+H	518.3241	518.3229,500.3025,459.1897,114.0658	LPC (18:3)	-2.3	Level 2
97	56.21	$C_{15}H_{16}O_3$	M+H	245.1176	245.1172,229.0857,181.1007	Curcumin C	1.5	Level 2
98	49.46	$C_{34}H_{47}NO_{10}$	M+H	630.3274	630.2002,570.1844,510.1603,105.0330	Isomer of 3-Deoxyaconine	0.2	Level 3
99	45.26	$C_{32}H_{43}NO_9$	M+H	586.3019	586.3020,554.2783,536.2647,526.2810	Isomer of 1,15-dimethoxy-3-hydroxy-14-benzoyl-16-ketoneoline	1.4	Level 3
100	49.95	$C_{22}H_{37}NO_2$	M+H	348.2904	348.2900,330.2788,312.2691	2-aminoethyl(5Z,8Z,11Z,14Z)-icosa-5,8,11,14-tetraenoate	2.0	Level 2
101	45.95	$C_{33}H_{45}NO_{11}$	M+H	632.3071	632.3050,572.2858,540.2614,105.0331	Mesaconitine	0.9	Level 1
102	48.54	$C_{33}H_{45}NO_{10}$	M+H	616.3101	616.3136,556.2924,524.2646,105.0316	Hypaconitine	-2.5	Level 1
103	45.54	$C_{31}H_{43}NO_7$	M+H	542.3126	542.3097,510.2853,492.2758,105.0332	Isomer of 14-Benzoylneoline	2.5	Level 3
104	45.97	$C_{34}H_{47}NO_{12}$	M+H	662.3174	662.3161,602.2973,570.2117,105.0330	10-hydroxyaconitine	0.4	Level 2
105	45.32	$C_{32}H_{45}NO_9$	M+H	588.3175	588.3021,556.2911,524.2670,105.0306	14-Benzoyldeoxyaconine	1.3	Level 2
106	47.41	$C_{33}H_{45}NO_8$	M+H	584.3232	584.3222522.2976,534.2886,58.0655	Patentine	2.4	Level 2
107	44.96	$C_{34}H_{47}NO_{11}$	M+H	646.3222	646.3219,586.3020,554.2758,105.0339	(-) - (A-b)-8β-acetoxy-14α-benzoyloxy-N-ethyl-3α,10β,13β-trihydroxy-1α,6α,16β,18-tetra-methoxyaconitane	2.5	Level 3
108	62.20	$C_{20}H_{37}NO_2$	M+H	324.2902	306.2797,288.2686,256.1093	Linoleoyl ethanolamide	1.5	Level 2

109	48.58	$C_{33}H_{46}NO_7$	M+H	568.3258	568.3284,550.3178,518.2933,58.0637	8-O-cinnamoylneoline	4.6	Level 2
110	47.39	$C_{33}H_{45}NO_9$	M+H	600.3169	600.3165,540.2953,508.2699,480.2749,105.0328	13-Deoxyhypaconitine	0.3	Level 2
111	50.56	$C_{34}H_{47}NO_9$	M+H	614.3329	614.3331,554.3103,522.2861,490.2592,105.0331	Chasmaconitine	0.9	Level 2
112	49.20	$C_{34}H_{47}NO_{10}$	M+H	630.3285	630.3261,570.3055,538.2805,510.2855,	3-Deoxyaconine	1.9	Level 2
113	49.31	$C_{15}H_{18}O_2$	M+H	231.1385	231.1383,213.1256,203.1444	Curzerenone	2.4	Level 2
114	26.33	$C_{15}H_{22}O_2$	M+H	235.1704	235.1679,217.1625,189.1632	Curcumenol	4.9	Level 2
115	49.63	$C_{27}H_{43}NO_5$	M+H	462.3215	444.3101,426.3013,337.2535,90.0548	6-Keto-glycohyodeoxycholic acid methyl ester	0.2	Level 3
116	49.65	$C_{27}H_{43}NO_5$	M+H	462.3214	444.3133,426.3012,337.2534,90.0548	7-Keto-glycochenodeoxycholic acid methyl ester	0.6	Level 3
117	49.65	$C_{27}H_{45}NO_6$	M+H	480.3321	462.2317,444.3109,426.2990,337.2520,90.0546	Glycohyocholic acid Methyl Ester	0.3	Level 3
118	49.15	$C_{34}H_{47}NO_9$	M+H	614.3331	614.3327,554.3126,522.2848,105.0333	Isomer of Chasmaconitine	1.2	Level 3
119	51.33	$C_{15}H_{22}O$	M+H	219.1748	219.1755,201.1649,191	Bisacumol	2.1	Level 2
120	52.30	$C_{23}H_{39}NO_2$	M+H	362.3061	362.3039,344.2947,326.2846,299.2365	N-(3-hydroxy-propyl) arachidonoylamide	2.1	Level 2
121	54.01	$C_{27}H_{45}NO_5$	M+H	464.3368	464.3573,428.3159,339.2678,90.0538	Glycochenodeoxycholic acid methyl ester	-0.5	Level 2
122	51.77	$C_{24}H_{38}O_4$	M+H	391.2857	373.2750,355.2619,337.2534,319.2425	7-Ketolithocholic acid	3.6	Level 2

123	53.79	$C_{20}H_{41}NO_2$	M+H	328.3214	328.3203,310.3105,292.1033,98.0967	N-(2-hydroxyethyl) stearamide	1.0	Level 3
124	53.77	$C_{18}H_{33}NO$	M+H	280.2634	280.2634,262.2530,250.2524,233.2276	Crucigasterin E	-0.3	Level 2
125	53.77	$C_{18}H_{35}NO_2$	M+H	298.2743	280.2632,262.2535,250.2536	(2S,3R)-2-amino-1,3-dihydroxyoctadec-4-yne	0.8	Level 3
126	54.14	$C_{18}H_{39}NO_3$	M+H	318.2998	318.2985,300.2882,282.2776,270.2785	Phytosphingosine	-1.5	Level 2
127	46.71	$C_{15}H_{18}O_3$	M+H	247.1335	229.1221,159.0806,139.0385	Zedoarol	2.5	Level 2
128	40.33	$C_{27}H_{45}NO_5$	M+H	464.3349	464.3270,428.3148,339.2680,90.0545	Glycohyodeoxycholic acid methyl ester	-4.6	Level 2
129	35.64	$C_{15}H_{20}O$	M+H	217.1594	217.1581,189.1629,105.0694	Furanodiene	3.3	Level 2
130	52.80	$C_{15}H_{22}O_2$	M+H	235.1699	235.1678,217.1593,177.1271,105.0694	Curcumenone	2.7	Level 2
131	55.81	$C_{18}H_{39}NO_2$	M+H	302.3054	302.3048,284.2948,266.2843,256.2135	Dihydrosphingosine	0.1	Level 2
132	55.45	$C_{15}H_{24}O_2$	M+H	237.1849	219.1747,191.1789,135.1166	Neocurdione	2.1	Level 2
133	56.86	$C_{20}H_{43}NO_2$	M+H	330.3367	330.3358,312.3264,88.0754	Eicosasphinganine	2.6	Level 2
134	57.29	$C_{18}H_{39}NO$	M+H	286.3111	268.2996,226.2499,180.9724,97.1005	N-Hexadecylethanolamine	2.3	Level 2
135	54.15	$C_{20}H_{37}NO_3$	M+H	340.2831	340.2823,294.1332,109.1007	Oleoylglycine	-4.5	Level 2
136	37.75	$C_{15}H_{18}O_2$	M+H	231.1390	231.1381,213.1274,173.0955,149.0595	Furanodienone	4.5	Level 2
137	56.65	$C_{26}H_{50}NO_7P$	M+H	520.3398	520.3394,502.3309,184.0730,10	LPC (18:2)	-1.9	Level 2

					4.1066			
138	57.16	$C_{28}H_{48}NO_7P$	M+H	542.3225	542.3230,483.2493,337.2733,146.9815,104.1065	LPC (20:5)	-3	Level 2
139	65.26	$C_{16}H_{30}O_2$	M+H	255.2317	255.2138,237.2204,219.2114	2Z-Hexadecenoic acid	0	Level 2
140	60.93	$C_{19}H_{38}O_4$	M+H	331.2848	331.2625,313.2524,295.2419,257.2261	Glycerolmonopalmitate	1.6	Level 2
141	58.36	$C_{24}H_{50}NO_7P$	M+H	496.3390	496.3391,478.3278,184.072,104.1067	LPC (16:0)	-1.5	Level 2
142	51.33	$C_{15}H_{22}O$	M+H	219.1748	219.1477,210.1638,159.1140	Germacrone	2.1	Level 2
143	57.96	$C_{15}H_{22}O$	M+H	219.1748	219.1736,201.1633,159.1163,105.0696	(+)-Nootkatone	2.1	Level 2
144	58.72	$C_{15}H_{22}$	M+H	203.1804	203.1805,147.1165,105.0692,119.0852	α-curcumene	2.8	Level 2
145	59.02	$C_{23}H_{46}NO_7P$	M+H	480.3085	462.2982,339.2894,308.2941,184.0733,104.1074	LPC (15:1)	1.1	Level 2
146	57.25	$C_{28}H_{50}NO_7P$	M+H	544.3398	544.3390,485.26246,339.2901,104.10769	LPC (20:4)	-0.7	Level 2
147	58.76	$C_{26}H_{52}NO_7P$	M+H	522.3554	522.3556,504.3556,184.0731,104.1067	LPC (18:1)	-1.8	Level 1
148	26.28	$C_{15}H_{20}O$	M+H	217.1597	217.1581,189.1648,175.1095,91.0522	ar-Turmerone	4.6	Level 2
149	60.77	$C_{15}H_{22}O$	M+H	219.1743	219.1736,201.1633,163.1116	(S)-turmerone	1.2	Level 2
150	55.20	$C_{18}H_{30}O_2$	M+H	279.2323	279.0936,149.0229,57.0693	Linolenic acid	1.6	Level 2
151	62.34	$C_{26}H_{54}NO_7P$	M+H	524.3699	524.3742,506.4509,184.0728,10	LPC (18:0)	-2.2	Level 1

					4.1070			
152	61.71	$C_{15}H_{24}$	M+H	205.1951	149.0226,121.0285	α-Zingiberene	1.6	Level 2
153	62.02	$C_{18}H_{34}O_2$	M+H	283.2637	265.2518,247.2416,191.1795,209.1889	8E,11-Octadecenoic acid	1.9	Level 3
154	62.89	$C_{30}H_{62}O_{10}$	M+H	583.4437	415.2556,133.0853	Nonaethylene glycol monododecyl ether	3.6	Level 2
155	63.02	$C_{28}H_{58}O_9$	M+H	539.4165	371.2284,133.0860	Dodecyl octaethylene glycol ether	2.1	Level 2
156	64.37	$C_{21}H_{38}O_4$	M+H	355.2849	355.2645,337.2538,263.2365,245.2278	Linoleic acid glyceride	1.7	Level 2
157	65.16	$C_{16}H_{33}NO$	M+H	256.2635	256.2620,116.1062,88.0751	Hexadecanamide	0.0	Level 2
158	65.72	$C_{21}H_{36}O_4$	M+H	353.2692	353.2679,335.2575,291.2321	Methyl 2,3-dihydroxylinolenate	1.6	Level 3
159	65.85	$C_{19}H_{38}O_4$	M+H	331.2846	331.2181313.2726,257.2477	Isomer of Glycerol monopalmitate	0.9	Level 3
160	66.84	$C_{24}H_{50}O_6$	M+H	435.3672	239.1494,133.0855	Polypentyl glycol monotetradecyl ether	-1.9	Level 2
161	1.22	$C_6H_{14}N_4O_2$	M+H	175.1192	60.0552,70.0645,84.0805,116.0700	Arginine	1.4	Level 2
162	4.63	$C_9H_{11}NO_3$	M+H	182.0820	136.0752,165.0533	Tyrosine	4.6	Level 2
163	2.34	$C_{11}H_{21}NO_7$	M+H	280.1402	262.1273	N-Fructosylvaline	4.0	Level 2
164	9.34	$C_{23}H_{37}NO_7$	M+H	440.2656	440.2639,422.2526,58.0641	9-Hydroxysenbusine A	3.0	Level 2
165	2.89	$C_5H_5N_5O$	M+H	152.0571	135.0311,110.0347	Guanine	2.7	Level 2
166	18.32	$C_{22}H_{31}NO_3$	M+H	358.2374	340.2284	Songorine	-0.8	Level 2
167	7.53	$C_9H_{11}NO_2$	M+H	166.0869	120.081	Phenylalanine	3.9	Level 2
168	14.17	$C_{22}H_{29}NO_3$	M+H	356.2222	356.2206,296.1628,278.1522	Songoramine	0.5	Level 2
169	19.42	$C_{20}H_{27}NO_3$	M+H	330.2067	330.2058,312.1952,294.1858	Hetisine	1.0	Level 2

170	18.69	$C_{22}H_{33}NO_3$	M+H	360.2535	342.2398,155.0849,143.0837,129.0690	12-epi-Napelline	0.5	Level 2
171	18.00	$C_{10}H_{10}O_4$	M+H	195.0661	107.0496,135.0424	Ferulic acid	4.7	Level 2
172	18.87	$C_8H_8O_4$	M+H	169.0501	65.0380,93.0328	Vanillic acid	3.3	Level 2
173	20.01	$C_9H_6O_3$	M+H	163.0397	117.0319,135.0443,145.0280	7-Hydroxycoumarin	4.5	Level 2
174	54.17	$C_{22}H_{29}NO_5$	M+H	388.2105	388.2492,144.0762,131.0754,89.0595	Guanfu base Y/isomer	-3.5	Level 3
175	28.40	$C_{26}H_{28}O_{14}$	M+H	565.1552	547.1444,529.1337,511.1230	Apiin	-0.7	Level 2
176	28.54	$C_{26}H_{41}NO_7$	M+H	480.2968	462.2837,380.0560,167.0844,58.0697	8-deoxy-14-dehydroaconosine	2.5	Level 3
177	58.00	$C_{12}H_{16}O_2$	M+H	193.1226	175.1095,147.0803	4-Pentylbenzoic acid	1.5	Level 2
178	31.28	$C_{24}H_{33}NO_5$	M+H	416.2643	328.1893,310.1788,195.1160,179.0884,155.0835	Guanfu base Z/isomer	2.8	Level 3
179	16.72	$C_{15}H_{20}O_5$	M+H	281.1389	281.0503,245.1181,157.0988,107.0512	Zedoalactone B	2.0	Level 2
180	32.42	$C_{21}H_{20}O_{10}$	M+H	433.1144	313.0692	Apigenin-7-O-β-D-glucopyranoside	3.4	Level 2
181	40.62	$C_{31}H_{43}NO_{10}$	M+H	590.2960	590.2954,540.2568,105.0341	Benzoylmesaconine/isomer	1.2	Level 3
182	26.12	$C_{24}H_{39}NO_5$	M+H	422.2904	390.2631	Talatizamine/isomer	0.7	Level 3
183	35.86	$C_{27}H_{30}O_{14}$	M+H	579.1723	433.1139,271.0598	Rhoifolin	2.5	Level 2
184	33.92	$C_{24}H_{22}O_{14}$	M+H	535.1060	535.2844,288.0565,287.0545,153.0625	Luteolin7-(6"-malonylglucoside)	-4.2	Level 3
185	42.17	$C_{18}H_{35}NO_4$	M+H	330.2639	250.2527,312.2524	N-Dodecoxycarbonylvaline	1.9	Level 2
186	20.15	$C_{11}H_{10}O_4$	M+H	207.0561	105.0681,119.0022,147.0454,15	Scoparone	-0.4	Level 2

					1.0745			
187	44.73	$C_{31}H_{41}NO_9$	M+H	572.2864	105.0327,95.0114,344.1693,554.2762	Dehydrated benzoylmesaconine	1.7	Level 2
188	44.46	$C_{12}H_{16}O_3$	M+H	209.1182	191.1058,165.1272	Senkyunolide K	4.7	Level 2
189	44.60	$C_{32}H_{43}NO_9$	M+H	586.3011	536.2634,131.0662,111.0794,105.0323,75.0433	1,15-dimethoxy-3-hydroxy-14-benzoyl-16-keto-neoline	2.1	Level 3
190	45.10	$C_{31}H_{35}NO_8$	M+H	550.2442	550.2418,428.2056,326.1772,308.1630,105.0332	(+) - (13R,19S)-1β,11α-diacetoxy-2α-benzoyloxy-13,19-dihydroxyhetisan	1.2	Level 3
191	45.12	$C_{33}H_{45}NO_9$	M+H	600.3072	58.0644,105.0324,540.2940,508.2677	Isodelphinine	1.9	Level 3
192	60.77	$C_{10}H_{14}O$	M+H	151.1123	123.0793,91.0543	Carvone	3.7	Level 2
193	29.78	$C_{15}H_{24}O_3$	M+H	253.1798	235.1702,217.1656,151.0804	Zedoarondiol/isomer	2.7	Level 3
194	48.65	$C_{32}H_{45}NO_8$	M+H	572.3229	572.3200,540.2960,508.2707,105.0332	14-O-Anisoylneoline	-1.4	Level 2
195	48.62	$C_{22}H_{35}NO_2$	M+H	346.2731	58.0640,105.0323,282.1695	Dihydroatisine	-3.9	Level 2
196	49.41	$C_{34}H_{47}NO_{10}$	M+H	630.3273	570.3044,538.2776,105.0419	3-Deoxyaconitine	-0.1	Level 2
197	52.85	$C_{22}H_{37}NO_2$	M+H	348.2897	330.2790,128.1049	N-(2-hydroxyethyl) Eicosa-5,8,11,14-tetraenamide	2.0	Level 2
198	61.76	$C_{15}H_{22}$	M+H	203.1794	203.1774,147.1166,119.0852,105.0696	Curcumene	1.8	Level 2
199	50.78	$C_{16}H_{35}NO_2$	M+H	274.2739	256.262	16-Dihydrosphingosine	-0.6	Level 3
200	51.02	$C_{18}H_{39}NO_3$	M+H	318.3005	102.0911,256.2631,300.2902	2-Amino-octadecane-1,3,4-triol	0.7	Level 3
201	23.51	$C_{23}H_{43}NO_2$	M+H	366.2703	344.2916	Semiplenamide A	3.7	Level 2

202	51.93	$C_{21}H_{35}NO$	M+H	318.2785	91.0537	Funtumine	-2.0	Level 2
203	55.18	$C_{18}H_{34}O_4$	M+H	315.2534	171.1042,127.1124	Octadecanedioic acid	1.3	Level 2
204	54.27	$C_{21}H_{39}NO$	M+H	322.3103	304.3019	2-(1-azepanylmethyl)-1-vinylcyclododecanol	-0.4	Level 2
205	53.23	$C_{24}H_{49}NO_8$	M+H	480.3532	462.3425,444.3268	(2S,3S,4R)-2-Amino-4-dihydroxyoctadecyl-β-D-galactopyranoside	0.2	Level 3
206	41.45	$C_{18}H_{16}O_6$	M+H	329.1029	314.0780,296.0674,268.0726,240.0765,225.0530	Dihydrochalcone	2.8	Level 2
207	56.61	$C_{27}H_{48}O_9$	M+H	517.3381	337.2741,263.2362,245.2274,95.0849,355.2788	2,3-dihydroxypropyl (9Z,12Z)-9,12-octadecadieno-ate-hexose	1.9	Level 3
208	57.48	$C_{21}H_{36}O_4$	M+H	353.2692	335.2597,261.2201,243.2100	Glyceryl Linolenate	1.6	Level 2
209	60.94	$C_{27}H_{46}O_9$	M+H	515.3189	497.3210,353.2677,261.2204,243.2180,335.2604	2,3-dihydroxypropyl (9Z,12Z,15Z)-9,12,15-octadecatrienoate-hexose	-5.0	Level 3
210	60.90	$C_{19}H_{38}O_4$	M+H	331.2849	313.2719,95.0850,239.2415	Monopalmitin	1.9	Level 2
211	28.81	$C_{10}H_8O_3$	M+H	177.0550	117.0335,145.0268,149.0571	Methoxycoumarin	2.1	Level 2
212	60.19	$C_{18}H_{30}O_3$	M+H	295.2275	107.0871,93.0689,91.0528,81.0695,67.0545	13-oxo-9E,11E-octadecadienoic acid	2.5	Level 2
213	25.82	$C_{22}H_{33}NO_2$	M+H	344.2585	326.2468,58.0627	Denudatine	1.4	Level 2
214	13.99	$C_{23}H_{37}NO_7$	M+H	440.2642	440.2614,422.2505	9-Hydroxyl-senbusine A/isomer	-0.2	Level 3
215	33.63	$C_{12}H_{18}O_3$	M+H	211.1285	149.0955,209.1166	Jasmonic acid	3.1	Level 2
216	26.15	$C_{24}H_{39}NO_5$	M+H	422.2901	390.2623	Talatizamine	1.2	Level 2
217	31.65	$C_{12}H_{16}O_4$	M+H	225.1118	207.1078,165.0905,192.9991	Senkyunolide H	-1.5	Level 2

218	38.48	$C_{32}H_{45}NO_{11}$	M+H	620.3074	556.2479,514.2616,105.0328	(-) - (A-c)-14α-benzoyloxy-3α,10β,13β,15α-tetrahydroxy-1α,6α,8β,16β,18-pentamethoxy-N-methylaconitane	1.4	Level 3
219	38.85	$C_{28}H_{47}NO_{8}$	M+H	526.3386	508.3256,364.2835	5,8,11,14-pentadecanamide	2.2	Level 3
220	53.02	$C_{18}H_{30}O_{4}$	M+H	311.2217	291.1952,185.1228,137.0235	9-oxo-11-(3-pentyloxiran-2-YL) undec-10-enoic acid	-4.8	Level 2
221	6.43	$C_{22}H_{35}NO_{6}$	M+H	410.2559	58.07,368.89,392.24,350.88	Isomer of N-Deethylaconine	3.6	Level 3
222	13.99	$C_{23}H_{37}NO_{7}$	M+H	440.2642	58.07,422.25,440.26	Isomer of 9-OH-senbusine A	-0.9	Level 3
223	15.76	$C_{23}H_{37}NO_{6}$	M+H	424.2705	58.07,108.08,406.06	Senbusine B/isomer	2.7	Level 3
224	16.31	$C_{23}H_{37}NO_{6}$	M+H	424.2691	58.07,406.26,388.25	Senbusine A	-0.6	Level 2
225	13.23	$C_{23}H_{37}NO_{7}$	M+H	440.2650	58.07,422.25,440.26	Isomer of 9-OH-senbusine	1.6	Level 3
226	8.27	$C_{22}H_{35}NO_{5}$	M+H	394.2589	58.07,376.25,358.24,326.21	Karakolidine	2.1	Level 2
227	17.18	$C_{23}H_{37}NO_{5}$	M+H	408.2744	58.07,390.26	Isotalatizidine	-0.1	Level 2
228	15.99	$C_{22}H_{35}NO_{5}$	M+H	394.2594	58.07,376.25,358.24,394.26	Isomer of aconicarmine	1.5	Level 3
229	7.67	$C_{22}H_{35}NO_{4}$	M+H	378.2653	58.07,360.25,342.24,378.26	Isomer of karakoline	3.7	Level 3
230	13.83	$C_{24}H_{39N}O_{7}$	M+H	454.2819	454.2784,422.2534,404.2445,58.0647	Foresticine	4.3	Level 2
231	7.83	$C_{22}H_{35}NO_{4}$	M+H	378.2652	58.07,360.25,360.25,378.27,332.22	Aconicarchamine A	3.5	Level 2
232	10.05	$C_{22}H_{35}NO_{5}$	M+H	394.2586	58.07,376.25,328.22	Chuanfumine	-0.5	Level 2
233	15.11	$C_{21}H_{33}NO_{4}$	M+H	364.2477	58.07,328.23,346.24	Isomer of 16-OH-cardiopetaline	-1.5	Level 3

234	15.61	$C_{22}H_{33}NO_4$	M+H	376.2488	58.07,308.92,358.24,376.25	Karakanine	1.5	Level 2
235	9.40	$C_{22}H_{33}NO_5$	M+H	392.2431	374.23,58.07,304.19	Hokbusine B	2.2	Level 2
236	16.19	$C_{22}H_{33}NO_4$	M+H	376.2494	58.07,358.24,308.92	Isomer of karakanine	3.1	Level 3
237	23.97	$C_{24}H_{37}NO_5$	M+H	420.2754	58.07,402.26,388.89	N-Ethylhokbusine B	2.3	Level 2
238	17.45	$C_{24}H_{37}NO_6$	M+H	436.2706	58.07,436.27,340.19	Isomer of guiwuline	2.8	Level 3
239	45.98	$C_{34}H_{47}NO_{12}$	M+H	662.3171	105.03,58.07,384.18,602.09	Aconifine/isomer	0.4	Level 3
240	31.99	$C_{26}H_{41}NO_6$	M+H	464.3014	58.07,432.27,382.24	Monoacetyltalizamine	1.6	Level 2
241	46.15	$C_{26}H_{41}NO_6$	M+H	464.3007	58.07,131.09,432.28,416.29	Monoacetyltalizamine/isomer	-1.4	Level 3
242	41.9	$C_{31}H_{41}NO_7$	M+H	540.2956	105.03,522.28,340.23	12-epi-15-O-Acetyl-17-Benzoyl-16-Hydroxy-16,17-Dihydronapelline/isomer	0	Level 3
243	48.58	$C_{33}H_{45}NO_7$	M+H	568.3258	131.05,58.07,103.05	Isomer of 8-O-cinnamoylneoline	-1.9	Level 3
244	12.28	$C_{22}H_{35}NO_7$	M+H	426.2496	426.2483,408.2379,390.2257,358.2003,107.0491,58.0653	Delbine	2.3	Level 2
245	4.58	$C_{11}H_{17}NO_2$	M+H	196.1339	196.1332,137.0597,119.0490,91.0541,65.0387	Coryneine	3.5	Level 2
246	4.66	$C_{10}H_{13}NO_2$	M+H	180.1024	180.1016,163.0751,145.0648,117.0698,115.0541,91.0541	(-)-Salsolinol	2.7	Level 2
247	10.11	$C_{24}H_{39}NO_{10}$	M+H	502.2658	502.2643,484.2541,470.2405,452.2277,420.2015,402.1888	10-OH-mesaconine	2.2	Level 2
248	12.19	$C_{22}H_{31}NO_7$	M+H	422.2187	422.2162,404.2023,386.1962,58.0653	Hohenackeridine	3.2	Level 2
249	11.12	$C_{20}H_{29}NO_4$	M+H	348.2179	348.2165,330.2061,300.1959,10	Aconicarnine A	2.8	Level 2

					5.0699			
250	15.02	$C_{22}H_{31}NO_6$	M+H	406.2234	406.2218,388.2119,360.1819,58.0653	Akirine	2.4	Level 2
251	18.5	$C_{21}H_{33}NO_3$	M+H	348.2542	348.2532,330.2426,312.2323,58.0653	Dictysine	2.5	Level 2
252	20.55	$C_{23}H_{35}NO_5$	M+H	406.2596	406.2588,388.2482,328.2278,58.0653	Nevadenine	2	Level 2
253	23.72	$C_{24}H_{37}NO_7$	M+H	452.2657	452.2653,434.2573,420.2377,402.2307,388.2102,58.0654	Delbruninol	3.1	Level 2
254	20.18	$C_{20}H_{27}NO_4$	M+H	346.2015	46.2013,328.1909,310.1788,144.0810,105.0698	3-Epiignavinol	0.6	Level 2
255	23.84	$C_{25}H_{39}NO_8$	M+H	482.2767	482.2747,450.2482,418.2238,386.1899,58.0653	18-Demethylpubescenine	3.8	Level 2
256	25.01	$C_{26}H_{41}NO_8$	M+H	496.2902	496.2908,436.2695,404.2418,376.2475,344.2217,58.0654	8-Acetyl-15-hydroxyneoline	-0.6	Level 3
257	26.81	C24H35NO8	M+H	466.2440	466.2434,448.2331,433.2767,416.2041,402.2273,370.2005	Kusnezosine C	1	Level 2
258	6.83	$C_{22}H_{33}NO_6$	M+H	408.2390	408.2368,390.2257,358.2007,330.1701,58.0653	Excelsine	2.3	Level 2
259	27.03	$C_{23}H_{35}NO_7$	M+H	438.2494	438.2473,420.2384,406.2237,388.2074,58.0654	Tuguaconitine	1.8	Level 2
260	24	$C_{26}H_{41}NO_8$	M+H	496.2926	496.2915,478.2803,464.2293,446.2538,414.2255,58.0654	14-O-Acetyldelectinine	4.2	Level 2
261	29.59	$C_{27}H_{41}NO_8$	M+H	508.2905	508.2905,490.2799,430.2589,398.2323,154.1226,58.0653	Deltaline	3.9	Level 2

262	32.58	$C_{22}H_{29}NO_4$	M+H	372.2169	372.2168,354.2068,312.1960,294.1849,266.1914	11-Acetylhetisine	0.7	Level 2
263	33.09	$C_{25}H_{39}NO_5$	M+H	434.2901	434.2902,402.2635,384.2514,370.2372,58.0653	14-Acetylsachaconitine	1.8	Level 2
264	41.85	$C_{25}H_{37}NO_6$	M+H	448.2694	448.2689,430.2578,388.2483,370.2376,342.2058,58.0654	16-Hydroxyl-vilmorisine	1	Level 2
265	62.25	$C_{24}H_{33}NO_4$	M+H	400.2496	400.2483,342.9508,314.2114,296.2007,280.2058	1-O-Acetylsongorine	3.4	Level 2
266	26.41	$C_{26}H_{39}NO_6$	M+H	462.2850	462.2852,430.2591,402.2633,370.2375,352.2277,58.0653	Vilmorrisine	2.8	Level 2
267	47.05	$C_{29}H_{39}NO_7$	M+H	514.2812	514.2800,482.2539,464.2433,446.2343,105.0334,58.0653	Carmichaenine D	2.5	Level 2
268	39.99	$C_{30}H_{41}NO_{10}$	M+H	576.2821	576.2803,558.2677,526.2445,494.2182,468.2041,105.0334	N-Deethyl-14-benzoylaconine	3.1	Level 2
269	40.24	$C_{26}H_{35}NO_5$	M+H	442.2604	442.2587,340.1908,322.1803,158.0963	11α-hydroxy-19β-carbonyl-N-methyl-13α-(2β-methylbutyryloxy)-2α-hydroxyhetisanium	3.6	Level 3
270	45.92	$C_{31}H_{43}NO_7$	M+H	542.3107	542.3114,510.2853,442.2231,105.0334,58.0653	14-Benzoylneoline	-1	Level 2
271	46.27	$C_{31}H_{43}NO_6$	M+H	526.3168	526.3165,494.2898,462.2630,105.0335,58.0653	14-Benzoyltalatisamine	0.9	Level 2
272	48.56	$C_{39}H_{41}NO_{11}$	M+H	700.2761	700.2748,640.2551,578.2380,105.0335	Trifoliolasine E	1.2	Level 2
273	65.03	$C_{24}H_{31}NO_4$	M+H	398.2319	398.2326,305.2475,149.0234,121.0284	15-Acetylsongoramine	-1.7	Level 2

274	63.44	$C_{49}H_{71}NO_{10}$	M+H	834.5143	834.5142,802.4905,556.2900,524.2645,496.2742,492.2376,464.2482,370.2009,338.1750,105.0335	8-linolen-benzoylhypaconine	-0.9	Level 2
275	64.15	$C_{50}H_{73}NO_{10}$	M+H	848.5293	848.5308,570.3068,538.2801,510.2867,352.1903	8-linolen-benzoyldeoxyaconine	-1.7	Level 2
276	63.69	$C_{49}H_{73}NO_{11}$	M+H	852.5256	852.5248,572.2850,540.2597,522.2482,512.2637,354.1700,105.0334	8-lino-benzoylmesaconine	-4.2	Level 2
277	64.49	$C_{50}H_{75}NO_{11}$	M+H	866.5395	866.5405,586.3008,554.2736,536.2645,526.2809,494.2529,404.2461,105.0336	8-lino-benzoylaconine	-2.1	Level 2
278	64.70	$C_{49}H_{73}NO_{10}$	M+H	836.5285	836.5304,556.2908,524.2644,496.2775,492.2381	8-lino-benzoylhypaconine	-2.7	Level 2
279	65.27	$C_{50}H_{75}NO_{10}$	M+H	850.5436	850.5459,570.3058,538.2795,510.2868,478.279,352.1905,105.0333	8-lino-benzoyldeoxyaconine	-3.3	Level 2
280	64.90	$C_{47}H_{73}NO_{11}$	M+H	828.5246	828.5256,572.2854,540.2580,512.2645,480.2374,354.1696,105.0335	8-pal-benzoylmesaconine	-1.3	Level 2
281	65.16	$C_{49}H_{75}NO_{11}$	M+H	854.5383	854.5409,804.5105,572.2857,540.2577,512.2648,354.1702,105.0334	8-Oleicacid-benzoylmesaconine	-3.5	Level 3
282	67.1	$C_{50}H_{75}NO_{9}$	M+H	834.5521	834.5510,575.5035,554.3106,522.2852,494.2918,462.2627,105.0333	8-lino-benzoyl-3,13-deoxyaconine	0.8	Level 2

283	66.34	$C_{50}H_{77}NO_{11}$	M+H	868.5567	868.5557,818.5156,586.3011,554.2743,494.2497,368.1857,105.0336	14-benzoylaconine-8-oleate	-0.3	Level 2
284	67.04	$C_{49}H_{75}NO_{10}$	M+H	838.5487	838.5465,556.2902,526.2644,496.2746,492.2383,464.2451,338.1750,105.0335	8-ole-benzoylhypaconine	2.8	Level 2
285	66.75	$C_{47}H_{73}NO_{10}$	M+H	812.5312	812.5303,556.2913,524.2648,496.2672,492.2394,464.2495,338.1752	8-pal-benzoylhypaconine	0.6	Level 2
286	67.75	$C_{48}H_{75}NO_{10}$	M+H	826.5455	826.5463,570.3061,538.2795,510.2875,105.0345	8-pal-benzoyldeoxyaconine	-1.1	Level 2
287	65.73	$C_{49}H_{77}NO_{10}$	M+H	840.5620	840.5609,556.2908,524.2642,496.2702,492.2367,460.2113	8-str-benzoylhypaconine	0.7	Level 2
288	44.14	$C_{29}H_{39}NO_6$	M+H	498.2873	480.2751,462.2589,434.2466	Delavaconitine/Episcopalisine	4.6	Level 3
289	18.73	$C_{24}H_{35}NO_6$	M+H	434.2537	402.2262,374.2377,356.1836,324.1476,296.1012	6-Acetylheteratisine	-3.2	Level 2
290	45.13	$C_{35}H_{47}NO_{12}$	M+H	674.3180	614.2975,582.2621	N-Deethyl-3-O-acetylyunaconitine	1.3	Level 2
291	48.72	$C_{32}H_{45}NO_7$	M+H	556.3280	524.2987,492.2732,474.2597,460.2458	Hemsleyaconitine A	2	Level 2
292	44.51	$C_{32}H_{43}NO_8$	M+H	570.3081	538.2798,510.2845,478.2560,446.2139,324.1981	6-O-Benzoyleldelidine	3.4	Level 2
293	30.25	$C_{15}H_{22}O_4$	M+H	267.1598	231.1351,102.1269	Zedoalactone A	2.7	Level 2
294	49.26	$C_{15}H_{20}O_3$	M+H	249.1482	231.1359,185.1337,133.1017	Turmericlactone A	-1.3	Level 2
295	46.71	$C_{15}H_{18}O_3$	M+H	247.1335	229.1213,201.1270,139.0385,123.0435	Curcolone	2.5	Level 2

296	56.02	$C_{15}H_{16}O_2$	M+H	229.1226	211.1105,159.0794,114.0910,83.0132	Curzeone	1.3	Level 2
297	58.72	$C_{15}H_{22}$	M+H	203.1804	147.1159,105.0695,93.0696,55.0542	ar curcumene	4.8	Level 2
298	7.63	$C_9H_8O_2$	M+H	149.0604	131,120,103	Cinnamic acid	4.7	Level 2
299	54.16	$C_{22}H_{36}O_5$	M+H	381.2636	363,337,281,243,207	Curcuminol C	-3.5	Level 2
300	56.91	$C_{23}H_{44}NO_7P$	M+H	478.2928	337.27,478.28	3-{[(2-aminoethoxy) (hydroxy) phosphoryl] oxy}-2-hydroxy-propyl-9,12-octadecadienoate	0.0	Level 3
301	57.99	$C_{21}H_{44}NO_7P$	M+H	454.2933	313.24,393.24,436.28	3-{[(2-aminoethoxy) (hydroxy) phosphoryl] oxy}-2-hydroxy-propylpalmitate	1.1	Level 3
302	68.14	$C_{16}H_{32}O_2$	M+H	257.2475	258.24,149.13	Palmitic acid		Level 2
303	1.45	$C_{11}H_{17}N_4O_9P$	M+H	381.0806	345.11,381.07	2',3'-dideoxy-3'-[(hydroxy-carbamoyl) amino]-3,4-dihydro-thymidine5'-(dihydrogen-phosphate)	1.9	Level 3
304	57.67	$C_{25}H_{48}O_9$	M+H	493.3371	239.24,331.28,493.32	(2S)-2,3-Dihydroxypropyl Hexosyl Palmitate	2.2	Level 3
305	57.64	$C_{31}H_{61}NO_{14}$	M+H	672.4165	331.28,239.24	3-hydroxypropylpalmitate-glc-glucosamine	0.9	Level 3
306	66.09	$C_{18}H_{32}O_2$	M+H	281.2475	245.23	Linoleic acid	5.0	Level 2
307	49.75	$C_{34}H_{47}NO_8$	M+H	598.3374	538.3217,506.2853,474.2668,460.2515	Dolichotine A	1.3	Level 2
308	39.15	$C_{27}H_{31}NO_5$	M+H	450.2275	450.2849,432.2748,400.2487,58.0646	Ignavine	1.1	Level 2

309	42.17	$C_{18}H_{35}NO_4$	M+H	330.2639	330.2633,312.2555,57.0337	N-(dodecyloxycarbonyl) valine	2.2	Level 2
310	12.09	$C_{21}H_{27}NO_4$	M+H	358.2013	358.2008,340.1905,312.1963,300.1963	Sczukidine	3.4	Level 2
311	49.26	$C_{15}H_{20}O_3$	M+H	249.1485	249.1541,231.1382,161.0949	Curdionolide B	-1.3	Level 2
312	51.35	C15H24O2	M+H	237.1849	219.1747,191.1789,135.1166	Curcumol	2.9	Level 2
313	63.14	$C_{26}H_{54}O_8$	M+H	495.3907	327.2002,133.0857	heptaethylene glycol monododecyl ether	3.1	Level 2
314	18.43	$C_{19}H_{20}O$	M+H	265.1576	265.0975,247.0874,219.1251	trans, trans-1,7-Diphenyl-1,3-heptadien-5-ol	-4.1	Level 3
315	60.93	$C_{19}H_{38}O_4$	M+H	331.2848	331.2625,313.2524,295.2419,257.2261	Glycerol monopalmitate	1.6	Level 2
316	62.89	$C_{30}H_{62}O_{10}$	M+H	583.4437	415.2556,133.0853	Nonaethylene glycol monododecyl ether	3.6	Level 2
317	65.85	$C_{19}H_{38}O_4$	M+H	331.2846	331.2625,313.2524,295.2419,257.2261	Isomer of Glycerol monopalmitate	0.9	Level 3
318	2.34	$C_{11}H_{21}NO_7$	M+H	280.1402	262.1273	N-Fructosyl valine	4.0	Level 2
319	33.92	$C_{24}H_{22}O_{14}$	M+H	535.1060	535.2844,288.0565,287.0545,153.0625	luteolin 7-O-(6"-O-malonylglucoside)	-4.2	Level 3
320	56.61	$C_{27}H_{48}O_9$	M+H	517.3381	337.2741,263.2362,245.2274,95.0849,355.2788	2,3-dihydroxypropyl (9Z,12Z)-9,12-octadecadienoate-hexose	1.9	Level 3
321	60.94	$C_{27}H_{46}O_9$	M+H	515.3189	497.3210,353.2677,261.2204,243.2180,335.2604	2,3-dihydroxypropyl (9Z,12Z,15Z)-9,12,15-octadecatrienoate-hexose	-5.0	Level 3
322	1.45	$C_{11}H_{17}N_4O_9P$	M+H	381.0806	345.11,381.07	2',3'-dideoxy-3'-［(hydroxycarbamoyl) amino］-3,4-dihydrothymidine5'-(dihydrogen phosphate)	1.9	Level 3
323	57.64	$C_{31}H_{61}NO_{14}$	M+H	672.4165	331.28,239.24	3-hydroxypropyl palmitate-glc-	0.9	Level 3

						glucosamine		

Table S4. Mass data of fermented Yaomu sample in positive ion.

Peak No.	RT (min)	Formula	iron	Mass (m/z)	Fragment Ions (m/z)	Identification	ppm	Confidence Level
1	1.33	$C_5H_{11}NO_2$	M+H	118.0863	118.0860,58.0645	Valine	0.4	Level 2
2	1.47	$C_7H_7NO_2$	M+H	138.0556	138.054,392.0491,94.0647,78.0336,	Trigonelline	4.7	Level 2
3	5.62	$C_6H_{13}NO_2$	M+H	132.1022	132.1121,116.0576,86.0962	Isoleucine	2.2	Level 3
4	2.57	$C_5H_5N_5$	M+H	136.0624	136.0614,119.0349,92.0240	Adenine	4.6	Level 2
5	4.15	$C_5H_4N_4O$	M+H	137.0458	137.0641,119.0348,110.0351	Hypoxanthine	4.5	Level 2
6	7.7	C_8H_9N	M+H	120.0813	120.0804,103.0540,77.0382	N-Benzylidene(methyl)amine	4.4	Level 3
7	5.69	$C_5H_9NO_4$	M+H	148.0604	148.0779,130.0485,84.0440	L-Glutamic acid	3.8	Level 2
8	4.58	$C_{10}H_{13}N_5O_5$	M+H	284.0989	152.0562,135.0305	Guanosine	4.8	Level 2
9	6.31	$C_{10}H_{13}N_5O_4$	M+H	268.1051	136.0612,119.0351	Adenosine	4	Level 2
10	4.84	$C_9H_{10}O_2$	M+H	151.0754	91.0534,77.0363	3-Phenylpropionic acid	4.9	Level 2
11	4.53	$C_5H_4N_4O_2$	M+H	153.0411	153.1245,136.0135,110.0353	2,6-Dihydroxypurine	2.6	Level 2
12	7.21	$C_{23}H_{37}NO_9$	M+H	472.2542	472.2563,440.2257,422.2173,404.2089,58.0647	Beiwutinine	0.2	Level 2
13	20.86	$C_{23}H_{37}NO_6$	M+H	424.2693	424.2698,406.2581,374.2336,58.0642	Isomer of Senbusine A	-0.2	Level 3
14	17.16	$C_{23}H_{37}NO_5$	M+H	408.2749	408.2754,390.2292,358.2407,58.0647,105.0698	Isotalatizidine	1.1	Level 3
15	15.35	$C_{24}H_{39}NO_9$	M+H	486.2705	486.2703,454.2648,436.2327,404.2075,58.0641	Isomer of Mesaconine	1.5	Level 2
16	13.1	$C_{11}H_{12}N_2O_2$	M+H	205.0977	188.0712,146.0600,	Tryptophan	2.7	Level 2
17	19.17	$C_{11}H_9NO_2$	M+H	188.0714	188.0401,170.0606,142.0652,115.0549	3-Amino-2-naphthoic acid	4.2	Level 2

18	18.92	$C_{22}H_{35}NO_6$	M+H	410.2549	410.2550,378.2559,328.1939,58.0646	N-Deethylaconine isomer	2.9	Level 3
19	15.02	$C_{21}H_{33}NO_4$	M+H	364.2484	364.2480,346.2381,328.2282,58.0649	16-Hydroxycardiopetaline	0.5	Level 2
20	11.2	$C_{24}H_{39}NO_9$	M+H	486.2704	486.2703,454.2648,436.2327,404.2075,58.0641	Mesaconine	1.3	Level 1
21	19.01	$C_{15}H_{29}NO_4$	M+H	288.2173	288.2162,270.2058,210.1853	N-Lauroyl-L-Serine	1.3	Level 2
22	16.15	$C_{16}H_{17}NO_3$	M+H	272.1290	255.1021,161.0599,237.0914,107.0489	Higenamine	3.2	Level 2
23	14.84	$C_{22}H_{33}NO_4$	M+H	376.2491	376.2491,358.2385,326.2127,58.0650	Beiwusine A	2.3	Level 2
24	16.66	$C_{24}H_{39}NO_7$	M+H	454.2801	454.2805,436.2708,404.2431,58.0657	Senbusine C	0.4	Level 2
25	16.75	$C_{22}H_{35}NO_4$	M+H	378.2635	378.2616,360.2516,342.2430,58.0646	Karakoline	-1	Level 2
26	17.02	$C_{23}H_{37}NO_5$	M+H	408.2740	408.2754,390.2292,358.2407,58.0647,105.0698	Talatisidine	-1.1	Level 2
27	11.79	$C_{23}H_{37}NO_7$	M+H	440.2659	440.26503,422.2574,408.2402,390.2308,58.0646	9-Hydroxysenbusine A	3.7	Level 2
28	17.3	$C_{23}H_{37}NO_6$	M+H	424.2704	424.2698,406.2581,374.2336,58.0642	Senbusine A	2.4	Level 2
29	18.39	$C_{25}H_{41}NO_9$	M+H	500.2858	500.2830,468.2598,450.2480,418.2230,58.0646	Aconine	0.8	Level 1
30	18.82	$C_{24}H_{39}NO_6$	M+H	438.2859	438.2844,406.2591,388.2491,58.0652	6-epi-Forsticine	2	Level 2
31	20.75	$C_{25}H_{41}NO_8$	M+H	484.2915	484.28897,466.2773,434.2557,5	Deoxyaconine	2.1	Level 2

					8.0637			
32	18.6	$C_{25}H_{39}NO_7$	M+H	466.2807	448.2686,416.2452,58.0644	Delbruine	1.7	Level 2
33	21.73	$C_{22}H_{35}NO_6$	M+H	410.2548	410.2550,378.2559,328.1939,58.0646	N-Deethylaconine	2.6	Level 2
34	19.99	$C_{23}H_{35}NO_6$	M+H	422.2539	422.2542,404.2094,372.2342,58.0651	Gadesine	0.4	Level 2
35	18.96	$C_{15}H_{16}O_2$	M+H	229.1231	229.1232 201.1252,187.0770	Curcumin A	3.5	Level 2
36	22.23	$C_{24}H_{37}NO_6$	M+H	436.2704	436.2318,418.2222,386.1962,58.0647	Guiwuline	2.4	Level 2
37	20.93	$C_{24}H_{39}NO_8$	M+H	470.2745	470.2725,438.2472,406.2224	Hypaconine	-0.7	Level 1
38	13.3	$C_{11}H_9NO_2$	M+H	188.0711	188.0401,170.0606,142.0652,115.0549	6-Amino-2-naphthoica cid	2.6	Level 3
39	24.05	$C_{25}H_{39}NO_6$	M+H	450.2864	450.2835,418.2607,400.2433,58.0645	Isomer of Condelphine	3.1	Level 3
40	10.05	$C_{24}H_{33}NO_5$	M+H	416.2430	416.2440,330.2075,312.1972,103.0387	Guanfu Base Z	-0.4	Level 2
41	20.4	$C_{24}H_{37}NO_6$	M+H	436.2700	436.2318,418.2222,386.1962,58.0647	Isomer of Guiwuline	1.5	Level 3
42	21.2	$C_{24}H_{39}NO_7$	M+H	454.2795	454.2805,436.2708,404.2431,58.0657	Fuziline	-0.9	Level 2
43	18.87	$C_{23}H_{37}NO_5$	M+H	408.2744	408.2754,390.2292,358.2407,58.0647,105.0698	Isotaatizidine	-0.1	Level 2
44	20.55	$C_{24}H_{37}NO_8$	M+H	468.2611	468.2945,436.2326,418.2596,386.2326,58.0646	1,7,8-Trihydroxy-16-methoxy-4-(methoxymethyl) aconitane-14-yl acetate	4.1	Level 3
45	23.83	$C_{24}H_{37}NO_5$	M+H	420.2743	420.2731,402.2638,384.2545,370.2380,58.0646	N-Ethylhokbusine B	-0.4	Level 2

46	35.49	$C_{27}H_{31}NO_5$	M+H	450.2290	450.2849,432.2748,400.2487,58.0646	Ignavine	3.3	Level 2
47	20.45	$C_{25}H_{39}NO_7$	M+H	466.2815	448.2686,416.2452,58.0644	Isomer of Delbrusine	3.4	Level 3
48	20.17	$C_{24}H_{39}NO_7$	M+H	454.2811	454.2805,436.2708,404.2431,58.0657	Isomer of Fuziline	2.6	Level 3
49	23.73	$C_{25}H_{41}NO_7$	M+H	468.2954	468.2976,436.2697,404.2454,58.0640	Isomer of Lycoctonin	-0.4	Level 3
50	31.6	$C_{26}H_{41}NO_7$	M+H	480.2964	480.2969,448.2692,430.2618,58.0663	Bullatine C	1.7	Level 2
51	28.88	$C_{22}H_{33}NO_3$	M+H	360.2533	360.2538,342.2432,298.2155,58.0642	Spiramine H	0.8	Level 2
52	29.03	$C_{23}H_{33}NO_6$	M+H	452.3008	420.2751388.2497,356.2219,58.0647	Giraldine F isomer	2	Level 3
53	29.06	$C_{25}H_{41}NO_6$	M+H	452.3007	420.2748,388.2489,356.2229,58.0645	Chasmanine	-0.4	Level 2
54	25.46	$C_{23}H_{37}NO_4$	M+H	392.2793	392.2785,360.2528,342.2428,58.0646	Sachaconitine	-0.6	Level 2
55	29.22	$C_{26}H_{28}O_{14}$	M+H	565.1549	563.1402,353.0659	Apigenin-6-C-β-D-Galactose-8-C-α-L-arabinopyranoside	-0.5	Level 3
56	10.05	$C_{22}H_{35}NO_5$	M+H	394.2588	394.2586,362.2327,330.2070,58.0642	Chuanfumine	-0.5	Level 2
57	14.49	$C_{22}H_{33}NO_5$	M+H	392.2431	392.2783,360.2530,342.2436,58.0648	Heteratisine or Pengshenine A	2.2	Level 3
58	25.51	$C_{25}H_{39}NO_6$	M+H	450.2865	450.2835,418.2607,400.2433,58.0645	Condelphine	3.3	Level 2
59	23.85	$C_{23}H_{33}NO_6$	M+H	420.2381	420.2751,388.2497,356.2219,58.	Giraldine F	2	Level 2

					0647			
60	24.24	$C_{24}H_{39}NO_6$	M+H	438.2855	438.2844,406.2591,388.2491,58.0652	Neoline	1.1	Level 2
61	27.09	$C_{15}H_{29}NO_3$	M+H	272.2230	254.2117,81.0693	Tridecanoylglycine	3.6	Level 2
62	40.66	$C_{27}H_{31}NO_5$	M+H	450.2290	450.2849,432.2748,400.2487,58.0646	Isomer of ignavine	3.3	Level 3
63	28.6	$C_{25}H_{41}NO_7$	M+H	468.2967	468.2976,436.2697,404.2454,58.0640	Lycoctonine	2.4	Level 2
64	27.13	$C_{26}H_{41}NO_7$	M+H	480.2974	480.2969,448.2692,430.2618,58.0663	Isomer of Bullatine C	3.8	Level 3
65	19.29	$C_{27}H_{43}NO_8$	M+H	510.3074	510.3079,492.2967,460.2708,58.0658	14-O-acetylbrowniine	2.5	Level 2
66	29.82	$C_{15}H_{24}O_3$	M+H	253.1798	253.1807,235.1696,177.1274,159.1168	Zedoarondiol	3.1	Level 2
67	39.37	$C_{31}H_{43}NO_9$	M+H	574.3022	574.2655,542.2393,510.2133,105.0334	Isomer of Benzoylhypacoitine	2	Level 3
68	49.29	$C_{15}H_{18}O_2$	M+H	231.1380	231.1114,203.0815	Epicurzerenone	2.8	Level 2
69	31.95	$C_{26}H_{41}NO_6$	M+H	464.2938	464.2967,432.2730,414.2651,58.0646	Monoacetyltalatisamine	-1.4	Level 2
70	34.29	$C_{31}H_{43}NO_{11}$	M+H	606.2909	556.2565,524.2301,105.0326	(-) - (A-b)-14α-benzoyloxy-3α,10β,13β,15α-pentahydroxy-1α,6α,16β,18-tetramethoxy-N-methylaconitane	1.7	Level 3
71	31.52	$C_{15}H_{20}O_4$	M+H	265.1439	247.1332,229.1219,201.1272	Curcumenolactone C	1.8	Level 2
72	38.48	$C_{15}H_{18}O$	M+H	215.1438	215.1429,197.1335,187.1481,167.0851	(+)-agassizin	3.5	Level 2

73	46.02	$C_{15}H_{20}O_3$	M+H	249.1492	249.1541,231.1382,161.0949	Curdionolide B	2.7	Level 2
74	36.33	$C_{22}H_{37}NO_3$	M+H	364.2860	364.2868,346.2746,328.2615,310.2540	N-(2-hydroxyethyl) eicosapentaenamide	3.8	Level 3
75	44.94	$C_{31}H_{41}NO_8$	M+H	556.2915	556.2554,524.2267,492.2075,105.0340	Dehydrated benzoylhypaconine	1.8	Level 2
76	35.33	$C_{31}H_{43}NO_{10}$	M+H	590.2975	590.2977,540.2570,508.2334,105.0323	Isomer of Benzoylmesaconine	2.6	Level 3
77	36.12	$C_{18}H_{35}NO_4$	M+H	330.2646	330.2633,312.2555,57.0337	N-[(dodecyloxy) carbonyl]-Valine	2.2	Level 2
78	61.09	$C_8H_4O_3$	M+H	149.0233	121.0278,93.0327	Phthalic anhydride	3.2	Level 2
79	52.46	$C_{21}H_{37}NO$	M+H	320.2955	302.2582,287.8937	(2S)-2-(dodecylamino)-3-phenyl-1-propanol	2.2	Level 2
80	53.45	$C_{22}H_{37}NO_2$	M+H	348.2898	348.2748,330.2423,312.2335,	Arachidonylethanolamide	0.3	Level 2
81	37.63	$C_{31}H_{43}NO_{10}$	M+H	590.2940	590.2977,540.2570,508.2334,105.0323	Benzoylmesaconine	-3.3	Level 1
82	36.13	$C_{31}H_{43}NO_9$	M+H	574.3019	574.2655,542.2393,510.2133,105.0334	Benzoylhypaconine	1.5	Level 1
83	43.33	$C_{32}H_{45}NO_{10}$	M+H	604.3141	604.3090,572.2861,554.2745,522.2495,105.0330	Benzoylaconine	4.1	Level 2
84	40.13	$C_{30}H_{41}NO_7$	M+H	528.2967	528.2956,510.2857,496.2714,105.0336	6-Demethoxy benzoylneoline	2.1	Level 2
85	48.28	$C_{15}H_{18}O_3$	M+H	247.1334	247.1346,229.1277,201.1262	Zederone	2.1	Level 2
86	39.58	$C_{26}H_{45}NO_6S$	M+H	500.3040	498.2845,454.2968,408.2911,79.9591	Taurochenodeoxycholic acid	-4.1	Level 2
87	41.44	$C_{33}H_{45}NO_9$	M+H	600.3172	600.2822,540.2615,508.2691,480.2749,105.0327	Isomer of 13-Deoxyhypaconitine	0.8	Level 3

88	15.16	$C_{21}H_{33}NO_4$	M+H	364.2492	364.2480,346.2381,328.2282,58.0649	Isomer of 16β-Hydroxycardiopetaline	2.6	Level 3
89	41.79	$C_{32}H_{43}NO_9$	M+H	586.3013	586.3022,554.2763,536.2724	1,15-dimethoxy-3-hydroxy-14-benzoyl-16-keto-neoline	0.4	Level 3
90	46.37	$C_{15}H_{20}O_2$	M+H	233.1543	233.1532,215.1440,175.1123	Furanogermenone	3	Level 2
91	46.63	$C_{34}H_{47}NO_{11}$	M+H	646.3221	646.3233,586.3121,554.2711,105.0335	Aconitine	-0.1	Level 1
92	35.44	$C_{15}H_{22}O_3$	M+H	251.1642	251.1627,233.1531,215.1442	Aerugidiol	2.9	Level 2
93	42.87	$C_{33}H_{45}NO_{12}$	M+H	648.3017	648.3013,588.2806,556.2553,105.0338	Beiwutine	0.4	Level 2
94	45.84	$C_{31}H_{41}NO_8$	M+H	556.2915	556.2554,524.2267,492.2075,105.0340	Isomer of Dehydratedbenzoylhypaconine	1.8	Level 3
95	40.55	$C_{30}H_{41}NO_7$	M+H	528.2952	528.2956,510.2857,496.2714,105.0336	(-) - (A-b)-14α-benzoyloxy-N-ethyl-1α,8β,15α-trihydroxy-16β,18-dimethoxyaconitane	-0.7	Level 3
96	40.16	$C_{31}H_{43}NO_8$	M+H	558.3059	558.3041,526.2795,508.2707,105.0332	(-) - (A-b)-14α-benzoyloxyN-ethyl-1α,8β,15α-trihydroxy-6α,16β,18-trimethoxyaconitane	-0.4	Level 3
97	43.31	$C_{32}H_{45}NO_9$	M+H	588.3159	588.3145,556.2897,524.2657,105.0336	Isomer of 14-benzoyldeoxyaconine	-1.4	Level 3
98	52.93	$C_{15}H_{20}O$	M+H	217.1590	217.1599,199.1512,161.0945,105.0700	Comosone II	1.4	Level 2
99	44.86	$C_{33}H_{45}NO_{11}$	M+H	632.3065	632.3066,572.2866,540.2593,508.2358,105.0327	10-Hydroxy-hypaconitine	-0.1	Level 2
100	47.4	$C_{15}H_{16}O_3$	M+H	245.1179	245.1172,229.0857,181.1007	Curcumin C	2.8	Level 2
101	43.75	$C_{34}H_{47}NO_{10}$	M+H	630.3273	630.2002,570.1844,510.1603,10	Isomer of 3-Deoxyaconine	1.6	Level 3

					5.0330			
102	44.5	$C_{32}H_{43}NO_9$	M+H	586.3018	586.3020,554.2783,536.2647,526.2810	1,15-Dimethoxy-3-hydroxy-14-benzoyl-16-keto-neoline isomer	1.3	Level 3
103	52.73	$C_{22}H_{37}NO_2$	M+H	348.2895	348.2748,330.2423,312.2335	2-aminoethyl(5Z,8Z,11Z,14Z)-icosa-5,8,11,14-tetraenoate	-0.6	Level 2
104	44.78	$C_{33}H_{45}NO_{11}$	M+H	632.3064	632.3066,572.2866,540.2593,508.2358,105.0327	Mesaconitine	-0.2	Level 1
105	46.65	$C_{33}H_{45}NO_{10}$	M+H	616.3130	616.3136,556.2924,524.2646,105.0316	Hypaconitine	2.2	Level 1
106	42.32	$C_{31}H_{43}NO_7$	M+H	542.3126	542.3115,510.2864,492.2797,105.0344	Isomer of 14-Benzoylneoline	2.5	Level 3
107	45.07	$C_{34}H_{47}NO_{12}$	M+H	662.3164	662.3161,602.2973,570.2117,105.0330	10-Hydroxyaconitine	-1.1	Level 2
108	42.26	$C_{32}H_{45}NO_9$	M+H	588.4096	588.3145,556.2897,524.2657,105.0336	14-Benzoyldeoxyaconine	-1.7	Level 2
109	46.12	$C_{33}H_{45}NO_8$	M+H	584.3216	584.3222,522.2976,534.2886,58.0655	Patentine	-0.3	Level 2
110	44.93	$C_{34}H_{47}NO_{11}$	M+H	646.3234	646.3233,586.3121,554.2711,105.0335	(-) - (A-b)-8β-Acetoxy-14α-benzoyloxy-N-ethyl-3α,10β,13β-trihydroxy-1α,6α,16β,18-tetramethoxyaconitane	1.9	Level 3
111	62.18	$C_{20}H_{37}NO_2$	M+H	324.2902	306.2797,288.2686,256.1093	Linoleoyl Ethanolamide	1.5	Level 2
112	46.99	$C_{33}H_{45}NO_7$	M+H	568.3283	568.3284,550.3178,518.2933,58.0637	8-O-cinnamoylneoline	2.5	Level 2
113	47.15	$C_{33}H_{45}NO_9$	M+H	600.3181	600.2822,540.2615,508.2691,480.2749,105.0327	13-Deoxyhypaconitine	2.3	Level 2

114	48.23	$C_{34}H_{47}NO_9$	M+H	614.3324	614.3331,554.3103,522.2861,490.2592105.0331	Chasmaconitine	3	Level 2
115	48.5	$C_{34}H_{47}NO_{10}$	M+H	630.3276	630.2002,570.1844,510.1603,105.0330	3-Deoxyaconine	0.5	Level 2
116	36.09	$C_{15}H_{18}O_2$	M+H	231.1387	231.1114,203.0815	Curzerenone	3.2	Level 2
117	59.09	$C_{15}H_{22}O_2$	M+H	235.1704	235.1679,217.1625,189.1632	Curcumenol	4.9	Level 2
118	49.64	$C_{27}H_{43}NO_5$	M+H	462.3223	444.3101,426.3013,337.2535,90.0548	6-Keto-glycohyodeoxycholic acid methyl ester	1.9	Level 3
119	48.51	$C_{27}H_{43}NO_5$	M+H	462.3222	444.3101,426.3013,337.2535,90.0548	7-Keto-glycochenodeoxycholic acid methyl ester	1.7	Level 3
120	49.64	$C_{27}H_{45}NO_6$	M+H	480.3327	462.2317,444.3109,426.2990,337.2520,90.0546	Glycohyocholic acid Methyl Ester	1.5	Level 2
121	50.32	$C_{34}H_{47}NO_9$	M+H	614.3324	614.3331,554.3103,522.2861,490.2592105.0331	Isomer of Chasmaconitine	1.4	Level 3
122	40.73	$C_{15}H_{22}O$	M+H	219.1754	219.1755,201.1649,191	Bisacumol	4.8	Level 2
123	52.18	$C_{23}H_{39}NO_2$	M+H	362.3059	362.3039,344.2947,326.2846,299.2365	N-(3-hydroxy-propyl) arachidonoylamide	1.5	Level 2
124	52.83	$C_{27}H_{45}NO_5$	M+H	464.3391	464.3573,428.3159,339.2678,90.0538	Glycochenodeoxycholic acid methyl ester	4.4	Level 2
125	51.79	$C_{24}H_{38}O_4$	M+H	391.2843	373.2750,355.2619,337.2534,319.2425	7-Ketolithocholic acid	2.3	Level 2
126	53.70	$C_{20}H_{41}NO_2$	M+H	328.3216	328.3203,310.3105,292.1033,98.0967	N-(2-hydroxyethyl) stearamide	1.8	Level 3
127	53.98	$C_{18}H_{33}NO$	M+H	280.2639	280.2634,262.2530,250.2524,233.2276	Crucigasterin E	1.5	Level 2

128	53.66	$C_{18}H_{35}NO_2$	M+H	298.2745	280.2632,262.2535,250.2536	(2S,3R)-2-amino-1,3-dihydroxyoctadec-4-yne	1.5	Level 3
129	54.01	$C_{18}H_{39}NO_3$	M+H	318.3000	318.2985,300.2882,282.2776,270.2785	Phytosphingosine	-0.9	Level 2
130	47.2	$C_{15}H_{18}O_3$	M+H	247.1336	247.1346,229.1277,201.1262	Zedoarol	3	Level 2
131	53.98	$C_{27}H_{45}NO_5$	M+H	464.3375	464.3573,428.3159,339.2678,90.0538	Glycohyodeoxycholic acid methyl ester	1	Level 2
132	56.86	$C_{15}H_{20}O$	M+H	217.1594	217.1599,199.1512,161.0945,105.0700	Furanodiene	3.3	Level 2
133	52.92	$C_{15}H_{22}O_2$	M+H	235.1699	235.1679,217.1625,189.1632	Curcumenone	2.7	Level 2
134	55.67	$C_{18}H_{39}NO_2$	M+H	302.3057	302.3048,284.2948,266.2843,256.2135	Dihydrosphingosine	1.1	Level 2
135	54.56	$C_{15}H_{24}O_2$	M+H	237.1854	219.1747,191.1789,135.1166	Neocurdione	2.1	Level 2
136	55.44	$C_{15}H_{24}O_2$	M+H	237.1855	219.1747,191.1789,135.1166	Curcumol	2.5	Level 2
137	56.8	$C_{20}H_{43}NO_2$	M+H	330.3367	330.3358,312.3264,88.0754	Eicosasphinganine	1	Level 2
138	57.14	$C_{18}H_{39}NO$	M+H	286.3104	268.2996,226.2499,180.9724,97.1005	N-Hexadecylethanolamine	0.2	Level 2
139	64.6	$C_{20}H_{37}NO_3$	M+H	340.2853	340.2823,294.1332,109.1007	Oleoylglycine	2	Level 2
140	56.84	$C_{15}H_{18}O_2$	M+H	231.1388	231.1114,203.0815	Furanodienone	3.7	Level 2
141	57.16	$C_{26}H_{50}NO_7P$	M+H	520.3398	520.3394,502.3309,184.0730,104.1066	LPC (18:2)	0.8	Level 2
142	57.15	$C_{28}H_{48}NO_7P$	M+H	542.3241	542.3230,483.2493,337.2733,146.9815,104.1065	LPC (20:5)	-2.2	Level 2
143	65.29	$C_{16}H_{30}O_2$	M+H	255.2316	255.2138,237.2204,219.2114	2Z-Hexadecenoic acid	-1	Level 2
144	58.38	$C_{24}H_{50}NO_7P$	M+H	496.3398	496.3391,478.3278,184.0729,104.1067	LPC (16:0)	3.3	Level 2

145	60.72	$C_{15}H_{22}O$	M+H	219.1743	219.1755,201.1649,191	Germacrone	2.1	Level 2
146	60.7	$C_{15}H_{22}O$	M+H	219.1748	219.1755,201.1649,191	(+)-Nootkatone	2.1	Level 2
147	58.59	$C_{15}H_{22}$	M+H	203.1801	203.1805,147.1165,105.0692,119.0852	α-curcumene	3.3	Level 2
148	58.98	$C_{23}H_{46}NO_7P$	M+H	480.3086	462.2982,339.2894,308.2941,184.0733,104.1074	LPC (15:1)	0.3	Level 2
149	34.83	$C_{28}H_{50}NO_7P$	M+H	544.3398	544.3390,485.26246,339.2901,104.10769	LPC (20:4)	-4.5	Level 2
150	59.41	$C_{26}H_{52}NO_7P$	M+H	522.3554	522.3556,504.3556,184.0731,104.1067	LPC (18:1)	2.1	Level 1
151	55.78	$C_{15}H_{20}O$	M+H	217.1592	217.1599,199.1512,161.0945,105.0700	ar-Turmerone	2.3	Level 2
152	51.34	$C_{15}H_{22}O$	M+H	219.1743	219.1755,201.1649,191	(S)-Turmerone	3	Level 2
153	58.75	$C_{18}H_{30}O_2$	M+H	279.2316	279.0936,149.0229,57.0693	Linolenic acid	-0.9	Level 2
154	60.28	$C_{26}H_{54}NO_7P$	M+H	524.3711	524.3742,506.4509,184.0728,104.1070	LPC (18:0)	2.9	Level 1
155	61.75	$C_{15}H_{24}$	M+H	205.1954	205.1929,149.0232,135.1184,109.1004	β-Elemene	1.6	Level 2
156	61.5	$C_{15}H_{24}$	M+H	205.1957	205.1929,149.0232,135.1184,109.1004	α-Zingiberene	3	Level 2
157	62.18	$C_{18}H_{34}O_2$	M+H	283.2630	265.2518,247.2416,191.1795,209.1889	8E,11-Octadecenoic acid	-0.6	Level 3
158	63.41	$C_{26}H_{54}O_8$	M+H	495.2722	327.2002,133.0857	Heptaethylene Glycol Monododecyl Ether	-3.9	Level 2
159	63.24	$C_{24}H_{50}O_7$	M+H	451.3629	283.1749,133.0869	Hexaethylene Glycol Monododecyl Ether	2.6	Level 2

160	63.67	$C_{21}H_{38}O_4$	M+H	355.2834	355.2645,337.2538,263.2365,245.2278	Linoleic acid glyceride	-2.5	Level 2
161	65.15	$C_{16}H_{33}NO$	M+H	256.2630	256.2620,116.1062,88.0751	Hexadecanamide	-1.9	Level 2
162	65.71	$C_{21}H_{36}O_4$	M+H	353.2696	353.2679,335.2575,291.2321	Methyl 2,3-dihydroxylinolenate	2.7	
163	66.89	$C_{24}H_{50}O_6$	M+H	435.3683	239.1494,133.0855	Polypentyl glycol monotetradecyl ether	0.7	Level 2
164	1.2	$C_6H_{14}N_4O_2$	M+H	175.1196	60.0552,70.0645,84.0805,116.0700	Arginine	3.7	Level 2
165	2.89	$C_6H_5NO_2$	M+H	124.0396	96.0433	Nicotinic acid	2.4	Level 2
166	13.42	$C_{23}H_{37}NO_7$	M+H	440.2653	440.2643,422.2549,390.2273,58.0647	9-Hydroxysenbusine A	2.3	Level 2
167	2.89	$C_5H_5N_5O$	M+H	152.0571	135.0311,110.0347	Guanine	2.7	Level 2
168	18.25	$C_{22}H_{31}NO_3$	M+H	358.2379	340.2284	Songorine	0.6	Level 2
169	4.46	$C_9H_{11}NO_2$	M+H	166.0866	120.081	Phenylalanine	2.1	Level 2
170	14.08	$C_{22}H_{29}NO_3$	M+H	356.2221	356.2206,296.1628,278.1522	Songoramine	0.2	Level 2
171	19.35	$C_{20}H_{27}NO_3$	M+H	330.2066	330.2058,312.1952,294.1858	Hetisine	0.7	Level 2
172	18.61	$C_{22}H_{33}NO_3$	M+H	360.2535	360.2538,342.2432,298.2155,58.0642	12-epi-Napelline	0.5	Level 2
173	18.5	$C_{10}H_{10}O_4$	M+H	195.0657	107.0496,135.0424	Ferulic acid	2.6	Level 2
174	25.63	$C_{22}H_{33}NO_2$	M+H	344.2585	326.2468,58.0627	Denudatine	0.3	Level 2
175	18.86	$C_8H_8O_4$	M+H	169.0495	65.0380,93.0328	Vanillic acid	2.7	Level 2
176	17.22	$C_9H_6O_3$	M+H	163.0390	117.0319,135.0443,145.0280	7-Hydroxycoumarin	-0.4	Level 2
177	28.65	$C_{24}H_{37}NO_5$	M+H	420.2743	420.2731,402.2638,384.2545,370.2380,58.0646	N-Ethylhokbusine B/isomer	-0.1	Level 3
178	26.96	$C_{22}H_{29}NO_5$	M+H	388.2137	388.2492,144.0762,131.0754,89.0595	Guanfu base Y/isomer	4.8	Level 3

179	28.54	$C_{26}H_{28}O_{14}$	M+H	565.1552	563.1402,353.0659	Apiin	0.6	Level 2
180	27.95	$C_{26}H_{41}NO_7$	M+H	480.2958	480.2969,448.2692,430.2618,58.0663	8-deoxy-14-dehydroaconosine	0.5	Level 3
181	57.98	$C_{12}H_{16}O_2$	M+H	193.1223	175.1095,147.0803	4-Pentylbenzoic acid	3.1	Level 2
182	23.82	$C_{24}H_{33}NO_5$	M+H	416.2447	416.2440,330.2075,312.1972,103.0387	Guanfu base Z/isomer	3.7	Level 3
183	28.42	$C_{15}H_{20}O_5$	M+H	281.1384	281.0503,245.1181,157.0988,107.0512	Zedoalactone B	4.4	Level 2
184	32.78	$C_{21}H_{20}O_{10}$	M+H	433.1143	313.0692	Apigenin-7-O-β-D-glucopyranoside	3.2	Level 3
185	33.93	$C_{31}H_{43}NO_{10}$	M+H	590.2967	590.2977,540.2570,508.2334,105.0323	Benzoylmesaconine/isomer	2.8	Level 3
186	25.92	$C_{24}H_{39}NO_5$	M+H	422.2901	390.2631	Talatizamine/isomer	0.9	Level 3
187	42.39	$C_{15}H_{10}O_6$	M+H	287.055	269.0444	Luteolin	4.1	Level 2
188	35.86	$C_{27}H_{30}O_{14}$	M+H	579.1723	433.1139,271.0598	Rhoifolin	2.5	Level 2
189	20.15	$C_{11}H_{10}O_4$	M+H	207.0561	105.0681,119.0022,147.0454,151.0745	Scoparone	-0.4	Level 2
190	40.38	$C_{31}H_{41}NO_9$	M+H	572.2854	105.0327,95.0114,344.1693,554.2762	Dehydrated benzoylmesaconine	-0.7	Level 2
191	44.47	$C_{12}H_{16}O_3$	M+H	209.1172	191.1058,165.1272	Senkyunolide K	-0.1	Level 2
192	44.46	$C_{32}H_{43}NO_9$	M+H	586.3018	586.3022,554.2763,536.2724	1,15-dimethoxy-3-hydroxy-14-benzoyl-16-ketoneoline	1.3	Level 2
193	42.27	$C_{18}H_{24}O_4$	M+H	305.1757	287.1002,259.1708	(-)-mono-(1R)-Menthyl phthalate	3.2	Level 2
194	42.87	$C_{31}H_{35}NO_8$	M+H	550.2446	550.2418,428.2056,326.1772,308.1630,105.0332	(+) - (13R,19S)-1β,11α-diacetoxy-2α-benzoyloxy-13,19-dihydroxyhetisan	2.1	Level 3
195	43.08	$C_{33}H_{45}NO_9$	M+H	600.3072	558.3041,526.2795,508.2707,105.0332	Isodelphinine	-0.4	Level 3

196	45.7	$C_{32}H_{45}NO_9$	M+H	588.3183	588.3145,556.2897,524.2657,105.0336	14-Benzoylpseudaconine	2.7	Level 2
197	32.19	$C_{15}H_{24}O_3$	M+H	253.1803	253.1807,235.1696,177.1274,159.1168	Zedoarondiol/isomer	1.9	Level 3
198	46.56	$C_{32}H_{45}NO_8$	M+H	572.3213	572.3200,540.2960,508.2707,105.0332	14-O-Anisoylneoline	-0.9	Level 2
199	62.16	$C_{22}H_{35}NO_2$	M+H	346.2731	58.0640,105.0323,282.1695	Dihydroatisine	-2.8	Level 2
200	48.52	$C_{34}H_{47}NO_{10}$	M+H	630.3276	630.2002,570.1844,510.1603,105.0330	3-Deoxyaconitine	0.5	Level 2
201	52.8	$C_{22}H_{37}NO_2$	M+H	348.2913	348.2748,330.2423,312.2335,	N-(2-hydroxyethyl) eicosa-5,8,11,14-tetraenamide	4.6	Level 2
202	59.38	$C_{15}H_{22}$	M+H	203.1794	203.1805,147.1165,105.0692,119.0852	Curcumene	3.3	Level 2
203	50.61	$C_{16}H_{35}NO_2$	M+H	274.2744	256.262	16-Dihydrosphingosine	1.3	Level 3
204	54.03	$C_{18}H_{39}NO_3$	M+H	318.3003	318.2985,300.2882,282.2776,270.2785	2-Amino-octadecane-1,3,4-triol	-1.2	Level 3
205	52.15	$C_{23}H_{43}NO_2$	M+H	366.2703	362.3039,344.2947,326.2846,299.2365	Semiplenamide A	1.8	Level 2
206	51.85	$C_{21}H_{35}NO$	M+H	318.2799	91.0537	Funtumine	2.4	Level 2
207	55.14	$C_{18}H_{34}O_4$	M+H	315.2535	171.1042,127.1124	Octadecanedioic acid	1.6	Level 2
208	54.16	$C_{21}H_{39}NO$	M+H	322.3104	304.3019	2-(1-azepanylmethyl)-1-vinylcyclododecanol	2	Level 2
209	57.73	$C_{30}H_{46}O_4$	M+H	471.3473	407.3357,453.3091	Pomonic acid	0.9	Level 2
210	54.72	$C_{24}H_{49}NO_8$	M+H	480.3533	462.3425,444.3268	(2S,3S,4R)-2-Amino-4-dihydroxyoctadecyl-β-D-galactopyranoside	0.4	Level 3

211	65.69	$C_{21}H_{36}O_4$	M+H	353.2703	353.2679,335.2575,291.2321	Glyceryllin olenate	4.7	Level 2
212	65.78	$C_{19}H_{38}O_4$	M+H	331.2841	331.2625,313.2524,295.2419,257.2261,	Monopalmitin	-0.6	Level 2
213	28.67	$C_{10}H_8O_3$	M+H	177.0553	117.0335,145.0268,149.0571	Methoxycoumarin	3.8	Level 2
214	64.37	$C_{21}H_{32}O_3$	M+H	333.2424	315.2307	Agallochin O	-0.1	Level 2
215	60.14	$C_{18}H_{30}O_3$	M+H	295.228	107.0871,93.0689,91.0528,81.0695,67.0545	13-oxo-9E,11E-octadecadienoic acid	4.2	Level 2
216	13.9	$C_{23}H_{37}NO_7$	M+H	440.2643	440.2614,422.2505	9-Hydroxyl-senbusine A/isomer	0.5	Level 3
217	25.97	$C_{24}H_{39}NO_5$	M+H	422.2897	390.2623	Talatizamine	-0.9	Level 2
218	25.01	$C_{12}H_{16}O_4$	M+H	225.1121	207.0306,165.0887,209.0283	Senkyunolide H	-4.2	Level 2
219	22.57	$C_{24}H_{37}NO_5$	M+H	420.2756	420.2731,402.2638,384.2545,370.2380,58.0646	N-ethylhokbusine B/isomer	2.7	Level 3
220	35.54	$C_{32}H_{45}NO_{11}$	M+H	620.3065	556.2479,514.2616,105.0328	(-) - (A-c)-14α-benzoyloxy-3α,10β,13β,15α-tetrahydroxy-1α,6α,8β,16β,18-pentamethoxy-N-methylaconitane	0.7	Level 3
221	44.63	$C_{18}H_{35}NO_4$	M+H	330.2643	330.2633,312.2555,57.0337	N-Dodecoxycarbonylvaline	1.3	Level 2
222	38.85	$C_{28}H_{47}NO_8$	M+H	526.3386	508.3256,364.2835	5,8,11,14-Pentadecanamide	2.2	Level 3
223	52.96	$C_{18}H_{30}O_4$	M+H	311.2206	291.1952,185.1228,137.0235	9-oxo-11-(3-pentyloxiran-2-YL) undec-10-enoicacid	-3.5	Level 3
224	6.94	$C_{22}H_{35}NO_6$	M+H	410.2550	58.07,368.89,392.24,350.88	Isomer of N-deethylaconine	3.1	Level 3
225	13.42	$C_{23}H_{37}NO_7$	M+H	440.2653	58.07,422.25,440.26	Isomer of 9-OH-senbusine A	2.3	Level 3
226	16.22	$C_{23}H_{37}NO_6$	M+H	424.2697	58.07,406.26,388.25	Senbusine A	0.8	Level 2
227	13.9	$C_{23}H_{37}NO_7$	M+H	440.2640	58.07,422.25,440.26	Isomer of 9-OH-senbusine A	-0.6	Level 3
228	8.29	$C_{22}H_{35}NO_5$	M+H	394.2587	58.07,376.25,358.24,326.21	Karakolidine	-0.3	Level 2

229	17.16	$C_{23}H_{37}NO_5$	M+H	408.2750	74.06,408.27,58.07,288.20	Isomer of isotalatizidine	1.3	Level 3
230	14.4	$C_{23}H_{37}NO_6$	M+H	424.2699	58.07,131.09,406.26,356.22	Senbusine B/isomer	1.3	Level 3
231	17.21	$C_{23}H_{37}NO_5$	M+H	408.2754	58.07,390.26	Isotalatizidine	2.3	Level 3
232	15.93	$C_{22}H_{35}NO_5$	M+H	394.2596	58.07,376.25,358.24,394.26	Isomer of aconicarmine	2	Level 3
233	7.76	$C_{22}H_{35}NO_4$	M+H	378.2646	58.07,360.25,342.24,378.26	Isomer of karakoline	1.9	Level 3
234	15.76	$C_{24}H_{39}NO_7$	M+H	454.2812	454.2784,422.2534,404.2445,58.0647	Foresticine	2.8	Level 2
235	7.83	$C_{22}H_{35}NO_4$	M+H	378.2648	58.07,360.25,360.25,378.27,332.22	Aconicarchamine A	2.4	Level 2
236	15.11	$C_{21}H_{33}NO_4$	M+H	364.2491	58.07,328.23,346.24	Isomer of 16-OH-cardiopetaline	2.4	Level 3
237	15.48	$C_{22}H_{33}NO_4$	M+H	376.2495	58.07,308.92,358.24,376.25	Karakanine	3.4	Level 2
238	9.47	$C_{22}H_{33}NO_5$	M+H	392.2441	374.23,58.07,304.19	hokbusine B	2.4	Level 2
239	16.11	$C_{22}H_{33}NO_4$	M+H	376.2493	58.07,358.24,308.92	Isomer of karakanine	2.8	Level 3
240	14.08	$C_{22}H_{29}NO_3$	M+H	356.2221	296.20,356.22,278.19	Songoramine	0.2	Level 2
241	23.88	$C_{24}H_{37}NO_5$	M+H	420.2749	58.07,402.26,388.89	N-ethylhokbusine B	1.1	
242	14.36	$C_{24}H_{37}NO_6$	M+H	436.2695	58.07,436.27,340.19	Isomer of guiwuline	0.3	Level 3
243	45.11	$C_{34}H_{47}NO_{12}$	M+H	662.3171	105.03,58.07,384.18,602.09	Aconifine/isomer	0	Level 3
244	31.98	$C_{26}H_{41}NO_6$	M+H	464.3014	58.07,432.27,382.24	Monoacetyltalizamine	1.6	Level 2
245	46.16	$C_{26}H_{41}NO_6$	M+H	464.3007	58.07,131.09,432.28,416.29	Monoacetyltalizamine/isomer	-1	Level 3
246	44.23	$C_{31}H_{41}NO_7$	M+H	540.2964	105.03,522.28,340.23	12-epi-15-O-acetyl-17-benzoyl-16-hydroxy-16,17-dihydronapelline/isomer	1.5	Level 3
247	47.02	$C_{33}H_{45}NO_7$	M+H	568.3281	131.05,58.07,103.05	Isomer of 8-O-cinnamoylneoline	2.1	Level 3
248	7.06	$C_{22}H_{35}NO_7$	M+H	426.2504	426.2483,408.2379,390.2257,358.2003,107.0491,58.0653	Delbine	4.2	Level 2
249	4.56	$C_{11}H_{17}NO_2$	M+H	196.1339	196.1332,137.0597,119.0490,91.	Coryneine	3.5	Level 2

					0541,65.0387			
250	4.56	$C_{10}H_{13}NO_2$	M+H	180.1024	180.1016,163.0751,145.0648,117.0698,115.0541,91.0541	(-)-Salsolinol	2.7	Level 2
251	10.1	$C_{24}H_{39}NO_{10}$	M+H	502.2656	502.2643,484.2541,470.2405,452.2277,420.2015,402.1888	10-OH-mesaconine	1.8	Level 2
252	12.02	$C_{21}H_{27}NO_4$	M+H	358.2021	358.2008,340.1905,312.1963,300.1963	Sczukidine	2.3	Level 2
253	12.11	$C_{22}H_{31}NO_7$	M+H	422.2186	422.2162,404.2023,386.1962,58.0653	Hohenackeridine	3	Level 2
254	11.07	$C_{20}H_{29}NO_4$	M+H	348.2184	348.2165,330.2061,300.1959,105.0699	Aconicarnine A	4.2	Level 2
255	14.91	$C_{22}H_{31}NO_6$	M+H	406.2240	406.2218,388.2119,360.1819,58.0653	Akirine	3.9	Level 2
256	18.5	$C_{21}H_{33}NO_3$	M+H	348.2545	348.2532,330.2426,312.2323,58.0653	Dictysine	3.4	Level 2
257	20.51	$C_{23}H_{35}NO_5$	M+H	406.2593	406.2588,388.2482,328.2278,58.0653	Nevadenine	1.2	Level 2
258	24.06	$C_{24}H_{37}NO_7$	M+H	452.3076	452.2653,434.2573,420.2377,402.2307,388.2102,58.0654	Delbruninol	-0.6	Level 2
259	20.09	$C_{20}H_{27}NO_4$	M+H	346.2015	346.2013,328.1909,310.1788,144.0810,105.0698	3-Epiignavinol	0.6	Level 2
260	22.95	$C_{25}H_{39}NO_8$	M+H	482.2770	482.2747,450.2482,418.2238,386.1899,58.0653	18-Demethylpubescenine	4.5	Level 2
261	24.91	$C_{26}H_{41}NO_8$	M+H	496.2909	496.2908,436.2695,404.2418,376.2475,344.2217,58.0654	8-Acetyl-15-hydroxyneoline	0.8	Level 2

262	6.94	$C_{22}H_{33}NO_6$	M+H	408.2396	408.2368,390.2257,358.2007,330.1701,58.0653	Excelsine	3.8	Level 2
263	11.64	$C_{23}H_{35}NO_7$	M+H	438.2498	438.2473,420.2384,406.2237,388.2074,58.0654	Tuguaconitine	2.7	Level 2
264	27.55	$C_{26}H_{41}NO_8$	M+H	496.2911	496.2915,478.2803,464.2293,446.2538,414.2255,58.0654	14-O-Acetyldelectinine	1.2	Level 2
265	29.69	$C_{27}H_{41}NO_8$	M+H	508.2905	508.2905,490.2799,430.2589,398.2323,154.1226,58.0653	Deltaline	3.6	Level 2
266	16.55	$C_{22}H_{29}NO_4$	M+H	372.2184	372.2168,354.2068,312.1960,294.1849,266.1914	11-Acetylhetisine	3.9	Level 2
267	31.6	$C_{25}H_{39}NO_5$	M+H	434.2901	434.2902,402.2635,384.2514,370.2372,58.0653	14-Acetylsachaconitine	1.4	Level 2
268	39.72	$C_{25}H_{37}NO_6$	M+H	448.2694	448.2689,430.2578,388.2483,370.2376,342.2058,58.0654	16-hydroxyl-vilmorisine	0.1	Level 2
269	31.95	$C_{24}H_{33}NO_4$	M+H	400.2489	400.2483,342.9508,314.2114,296.2007,280.2058	1-O-Acetylsongorine	1.7	Level 2
270	33.55	$C_{26}H_{39}NO_6$	M+H	462.2850	462.2852,430.2591,402.2633,370.2375,352.2277,58.0653	Vilmorrisine	0.4	Level 2
271	25.54	$C_{24}H_{35}NO_8$	M+H	466.2451	466.2437,448.2330,433.2776,402.2274,58.0653	Kusnezosine C	3.3	Level 2
272	37.53	$C_{29}H_{39}NO_7$	M+H	514.2799	514.2800,482.2539,464.2433,446.2343,105.0334,58.0653	Carmichaenine D	1.9	Level 2
273	37.37	$C_{30}H_{41}NO_{10}$	M+H	576.2811	576.2803,558.2677,526.2445,494.2182,468.2041,105.0334	N-Deethyl-14-benzoylaconine	1.3	Level 2

274	39.64	$C_{26}H_{35}NO_5$	M+H	442.2588	442.2587,340.1908,322.1803,158.0963	11α-hydroxy-19β-carbonyl-N-methyl-13α-(2β-methylbutyryloxy)-2α-hydroxyhetisanium	0.7	Level 3
275	44.88	$C_{31}H_{43}NO_7$	M+H	542.3112	542.3114,510.2853,442.2231,105.0334,58.0653	14-benzoylneoline	-1.5	Level 2
276	45.49	$C_{31}H_{43}NO_6$	M+H	526.3163	526.3165,494.2898,462.2630,105.0335,58.0653	14-Benzoyltalatisamine	2.3	Level 2
277	47.5	$C_{39}H_{41}NO_{11}$	M+H	700.2752	700.2748,640.2551,578.2380,105.0335	Trifoliolasine E	-0.1	Level 2
278	65.03	$C_{24}H_{31}NO_4$	M+H	398.2339	398.2326,305.2475,149.0234,121.0284	15-Acetylsongoramine	3.3	Level 2
279	63.43	$C_{49}H_{71}NO_{10}$	M+H	834.5147	834.5142,802.4905,556.2900,524.2645,496.2742,492.2376,464.2482,370.2009,338.1750,105.0335	8-linolen-benzoylhypaconine	-0.4	Level 2
280	64.1	$C_{50}H_{73}NO_{10}$	M+H	848.5318	848.5308,570.3068,538.2801,510.2867,352.1903	8-linolen-benzoyldeoxyaconine	1.3	Level 2
281	63.63	$C_{49}H_{73}NO_{11}$	M+H	852.5240	852.5248,572.2850,540.2597,522.2482,512.2637,354.1700,105.0334	8-lino-benzoylmesaconine	-1.9	Level 2
282	64.44	$C_{50}H_{75}NO_{11}$	M+H	866.5404	866.5405,586.3008,554.2736,536.2645,526.2809,494.2529,404.2461,105.0336	8-lino-benzoylaconine	-1	Level 2
283	64.68	$C_{49}H_{73}NO_{10}$	M+H	836.5291	836.5304,556.2908,524.2644,496.2775,492.2381	8-lino-benzoylhypaconine	-1	Level 2

284	65.27	$C_{50}H_{75}NO_{10}$	M+H	850.5471	850.5459,570.3058,538.2795,510.2868,478.279,352.1905,105.0333	8-lino-benzoyldeoxyaconine	0.9	Level 2
285	64.51	$C_{47}H_{73}NO_{11}$	M+H	828.5261	828.5256,572.2854,540.2580,512.2645,480.2374,354.1696,105.0335	8-pal-benzoylmesaconine	0.6	Level 2
286	65.27	$C_{49}H_{75}NO_{11}$	M+H	854.5404	854.5409,804.5105,572.2857,540.2577,512.2648,354.1702,105.0334	8-Oleicacid-benzoylmesaconine	-1	Level 3
287	66.54	$C_{50}H_{75}NO_{9}$	M+H	834.5536	834.5510,575.5035,554.3106,522.2852,494.2918,462.2627,105.0333	8-lino-benzoyl-3,13-deoxyaconine	2.6	Level 2
288	66.15	$C_{50}H_{77}NO_{11}$	M+H	868.5583	868.5557,818.5156,586.3011,554.2743,494.2497,368.1857,105.0336	14-benzoylaconine-8-oleate	1.6	Level 2
289	66.65	$C_{49}H_{75}NO_{10}$	M+H	838.5464	838.5465,556.2902,526.2644,496.2746,492.2383,464.2451,338.1750,105.0335	8-ole-benzoylhypaconine	0	Level 2
290	66.14	$C_{47}H_{73}NO_{10}$	M+H	812.5317	812.5303,556.2913,524.2648,496.2672,492.2394,464.2495,338.1752	8-pal-benzoylhypaconine	1.2	Level 2
291	67.11	$C_{48}H_{75}NO_{10}$	M+H	826.5461	826.5463,570.3061,538.2795,510.2875,105.0345	8-pal-benzoyldeoxyaconine	-0.3	Level 2
292	65.65	$C_{49}H_{77}NO_{10}$	M+H	840.5620	840.5609,556.2908,524.2642,496.2702,492.2367,460.2113	8-str-benzoylhypaconine	0.1	Level 2
293	34.18	$C_{29}H_{39}NO_{6}$	M+H	498.2857	480.2751,462.2589,434.2466	Delavaconitine/Episcopalisine	1.4	Level 3

294	18.19	$C_{24}H_{35}NO_6$	M+H	434.2537	402.2262,374.2377,356.1836,324.1476,296.1012	6-Acetylheteratisine	3.4	Level 2
295	43.62	$C_{35}H_{47}NO_{12}$	M+H	674.3190	614.2975,582.2621	N-Deethyl-3-O-acetylyunaconitine	2.8	Level 2
296	47.06	$C_{32}H_{45}NO_7$	M+H	556.3287	524.2987,492.2732,474.2597,460.2458	Hemsleyaconitine A	3.3	Level 2
297	45.22	$C_{32}H_{43}NO_8$	M+H	570.3085	538.2798,510.2845,478.2560,446.2139,324.1981	6-O-Benzoyleldelidine	4.1	Level 2
298	49.16	$C_{34}H_{47}NO_8$	M+H	598.3381	538.3217,506.2853,474.2668,460.2515	Dolichosin A	1.1	Level 2
299	56.86	$C_{15}H_{16}O$	M+H	213.1274	196.1306,123.0923,102.1272,51.0223	Pyrocurzerenone	1.4	Level 2
300	30.24	$C_{15}H_{22}O_4$	M+H	267.1591	231.1351,102.1269	Zedoalactone A	1.9	Level 2
301	50.27	$C_{15}H_{20}O_4$	M+H	265.1434	247.1332,229.1219,201.1272	Zedoarofuran	2.9	Level 2
302	46.02	$C_{15}H_{20}O_3$	M+H	249.1485	231.1359,185.1337,133.1017	Turmericlactone A	2.7	Level 2
303	48.28	$C_{15}H_{18}O_3$	M+H	247.1329	229.1213,201.1270,139.0385,123.0435	Curcolone	2.1	Level 2
304	7.88	$C_9H_8O_2$	M+H	149.0604	131,120,103	Cinnamic acid	4.7	Level 2
305	54.14	$C_{22}H_{36}O_5$	M+H	381.2636	363,337,281,243,207,000	Curcuminol C	-4.1	Level 2
306	62.13	$C_{15}H_{26}O$	M+H	223.2056	188.99,207.03	(+)-Cedrol	1.4	Level 2
307	68.08	$C_{16}H_{32}O_2$	M+H	257.2475	258.24,149.13	Palmitic acid	1.5	Level 2
308	66.01	$C_{18}H_{32}O_2$	M+H	281.2480	245.23	Linoleic acid	1.8	Level 2
309	4.94	$C_9H_{11}NO_3$	M+H	182.0820	136.0752,165.0533	Tyrosine	4.6	Level 2
310	46.02	$C_{15}H_{20}O_3$	M+H	249.1492	249.1541,231.1382,161.0949	Curdionolide B	2.7	Level 2

Table S5. Significantly different compounds up- and down-regulated in unfermented Yaomu vs fermented Yaomu.

NO.	Compound	VIP	P	FC	log_2(FC)	WFJ vs. YM
P1	Monopalmitin	1.55	1.20E-09	8.88E+01	6.47	↑
P2	Guanfu base Y/isomer	1.55	7.26E-09	5.93E+00	2.57	↑
P3	LPC (16:1)	1.54	6.05E-10	3.24E-02	-8.34	↓
P4	Taurochenodeoxycholic acid	1.54	4.97E-09	5.44E+02	9.09	↑
P5	Carmichaeline D	1.53	1.42E-08	1.10E+02	6.77	↑
P6	Glycochenodeoxycholic acid methyl ester	1.53	6.19E-08	2.13E-01	-2.23	↓
P7	LPC (18: 0)	1.52	5.05E-08	7.42E-02	-9.53	↓
P8	Zedoarofuran	1.52	9.54E-08	5.95E+00	2.57	↑
P9	Zedoarol	1.52	1.02E-07	5.09E+00	2.35	↑
P10	Linoleic acid glyceride	1.51	3.90E-07	1.81E+01	4.18	↑
P11	Hemsleyaconitine A	1.50	6.80E-07	5.26E-04	-10.89	↓
P12	Trifoliolasine E	1.50	5.71E-07	7.59E+02	9.57	↑
P13	LPC (20:5)	1.50	8.74E-07	7.60E+01	6.25	↑
P14	Arginine	1.49	1.54E-06	3.72E+01	5.22	↑
P15	7-Ketolithocholic acid	1.49	1.45E-06	2.87E-01	-1.80	↓
P16	Linoleic acid	1.49	1.80E-06	3.05E+00	1.61	↑
P17	LPC (20:4)	1.48	2.55E-06	2.05E+02	7.68	↑
P18	Tridecanoylglycine	1.48	2.31E-06	1.05E-02	-6.58	↓
P19	Scoparone	1.48	3.83E-06	2.85E-01	-1.81	↓
P20	10-Hydroxyaconitine	1.47	3.68E-06	2.09E+03	11.03	↑
P21	13-Oxo-9E,11E-octadecadienoic acid	1.47	1.07E-05	2.82E+00	1.49	↑
P22	Isomer of 8-O-Cinnamoylneoline	1.47	6.76E-06	1.22E-03	-9.68	↓
P23	13-Deoxyhypaconitine	1.47	8.48E-06	2.51E-03	-8.64	↓
P24	(-) - (A-b)-8β-acetoxy-14α-benzoyloxy-N-ethyl-3α,10β,13β-trihydroxy-1α,6α,16β,18-tetra-methoxyaconitane	1.47	1.09E-05	2.39E-02	-5.39	↓
P25	2-Aminooctadecane-1,3,4-triol	1.47	6.17E-06	1.07E-01	-3.23	↓
P26	14-Benzoyldeoxyaconine	1.46	1.25E-05	1.46E-01	-2.77	↓

P27	Aconitine	1.46	9.26E-06	5.90E+01	5.88	↑
P28	Isomer of 13-Deoxyhypaconitine	1.46	1.95E-05	1.20E-02	-6.38	↓
P29	Dodecyl octaethylene glycol ether	1.46	1.46E-05	3.72E+01	5.22	↑
P30	LPC (15:1)	1.45	1.51E-05	1.66E-01	-4.06	↓
P31	14-Benzoylneoline	1.45	1.12E-05	5.46E+01	5.77	↑
P32	Dehydrated Benzoylhypaconine	1.45	2.29E-05	1.52E-02	-6.04	↓
P33	LPC (18:2)	1.45	1.70E-05	8.83E-01	-6.46	↓
P34	Isomer of Dehydrated Benzoylhypaconine	1.44	1.67E-05	1.10E-01	-3.18	↓
P35	10-Hydroxy-hypaconitine	1.44	1.46E-05	2.79E+01	4.80	↑
P36	2Z-Hexadecenoic acid	1.44	3.11E-05	2.02E+00	1.01	↑
P37	Isomer of Benzoylmesaconine	1.43	2.72E-05	6.26E-03	-7.32	↓
P38	7-Ketoglycochenodeoxycholic acid methyl ester	1.43	2.80E-05	2.11E-01	-2.25	↓
P39	(-) - (A-b)-14α-benzoyloxy-N-ethyl-1α,8β,15α-trihydroxy-6α,16β,18-trimethoxyaconitane	1.42	3.44E-05	7.95E-04	-10.30	↓
P40	LPC (18:1)	1.42	4.78E-05	2.10E-02	-7.72	↓
P41	Benzoylhypaconine	1.42	3.10E-05	2.20E-02	-5.51	↓
P42	Mesaconitine	1.42	3.82E-05	1.92E+04	14.23	↑
P43	Isomer of 14-Benzoyldeoxyaconine	1.41	9.62E-05	6.93E-03	-7.17	↓
P44	Palmitoylethanolamide	1.41	8.98E-05	4.35E+00	2.12	↑
P45	(+) - (13R,19S)-1β,11α-diacetoxy-2α-benzoyloxy-13,19-dihydroxyhetisan	1.40	1.17E-04	1.01E+02	6.66	↑
P46	Chasmaconitine	1.40	9.83E-05	1.75E+02	7.46	↑
P47	Furanogermenone	1.40	7.82E-05	7.94E-02	-3.65	↓
P48	3-Deoxyaconitine	1.40	1.04E-04	2.69E-02	-5.22	↓
P49	16-Dihydrosphingosine	1.40	9.31E-05	1.38E+01	3.79	↑
P50	Beiwutine	1.38	8.16E-05	3.48E+03	11.77	↑
P51	Isomer of 1,15-dimethoxy-3-hydroxy-14-benzoyl-16-keto-neoline	1.37	1.70E-04	6.90E+00	2.79	↑

P52	Crucigasterin E	1.37	3.02E-04	7.88E-02	-3.67	↓
P53	(2S,3S,4R)-2-amino-4-dihydroxyoctadecyl-β-D-galactopyranoside	1.37	1.90E-04	2.88E-01	-1.79	↓
P54	Comosone II	1.36	1.60E-04	6.63E+00	2.73	↑
P55	Hypaconitine	1.36	2.93E-04	5.04E+01	5.66	↑
P56	Dihydroatisine	1.35	2.55E-04	2.55E+00	1.35	↑
P57	Dehydrated benzoylmesaconine	1.34	3.72E-04	1.92E-01	-2.38	↓
P58	N-(2-Hydroxyethyl)-Eicosapentaenoic acid	1.33	3.18E-04	1.04E-02	-6.59	↓
P59	Cucumin C	1.33	3.19E-04	9.75E-02	-3.36	↓
P60	Isomer of benzoylhypacoitine	1.32	3.68E-04	1.20E-01	-3.06	↓
P61	Benzoylmesaconine	1.32	5.19E-04	2.05E-04	-12.25	↓
P62	N-Deethyl-3-O-acetyl-yunaconitine	1.31	5.82E-04	5.66E+01	5.82	↑
P63	Deoxyaconine	1.31	7.52E-04	1.33E-01	-2.91	↓
P64	Isodelphinine	1.30	1.02E-03	3.81E+00	1.93	↑
P65	Octadecanedioic acid	1.29	1.43E-03	2.86E-01	-1.81	↓
P66	Curcumenolactone C	1.29	1.08E-03	4.14E+00	2.05	↑
P67	Oleoyl glycine	1.29	1.13E-03	3.44E+00	1.78	↑
P68	N-(2-Hydroxyethyl) stearamide	1.29	8.48E-04	4.39E-01	-1.19	↓
P69	Delbruine	1.29	9.75E-04	1.73E-02	-5.85	↓
P70	8-Linoleoylbenzoylmesaconine	1.28	1.13E-03	2.81E+00	1.49	↑
P71	14-O-Acetylneoline	1.28	1.52E-03	4.35E-02	-4.52	↓
P72	18-Demethylpubescenine	1.28	1.44E-03	9.54E+00	3.25	↑
P73	Dihydrosphingosine	1.28	1.55E-03	3.07E-01	-1.70	↓
P74	1,15-dimethoxy-3-hydroxy-14-benzoyl-16-ketoneoline	1.27	1.32E-03	2.64E-02	-5.24	↓
P75	(-) - (A-c)-14α-benzoyloxy-3α,10β,13β,15α-tetrahydroxy-1α,6α,8β,16β,18-pentamethoxy-N-methylaconitane	1.27	1.32E-03	2.62E-02	-5.25	↓
P76	Glyceryl Linolenate	1.26	1.33E-03	4.08E+00	2.03	↑

P77	Linolenic acid	1.25	2.77E-03	3.02E+00	1.59	↑
P78	Hypaconine	1.24	1.86E-03	2.00E-02	-5.64	↓
P79	Aconitane-1,8,14-triol	1.23	1.70E-03	1.04E-01	-3.26	↓
P80	Isomer of Chasmaconitine	1.22	2.72E-03	1.49E-03	-9.39	↓
P81	8-O-cinnamoylneoline	1.20	2.12E-03	1.04E-01	-3.26	↓
P82	Linoleoylethanolamide	1.19	6.00E-03	2.66E+00	1.41	↑
P83	2-Aminoethyl (5Z,8Z,11Z,14Z)-Icosa-5,8,11,14-tetraenoate	1.18	5.53E-03	2.37E+00	1.24	↑
P84	Gadesine	1.18	5.35E-03	2.48E-01	-2.01	↓
P85	3-epi-ignavinol	1.15	6.24E-03	1.62E-01	-2.63	↓
P86	14-O-acetylkarakoline	1.15	6.41E-03	1.83E-01	-2.45	↓
P87	Isomer of 14-Benzoylneoline	1.14	4.87E-03	1.39E-01	-2.85	↓
P88	14-O-anisoylneoline	1.13	7.05E-03	2.30E+01	4.52	↑
P89	Sachaconitine	1.12	1.01E-02	4.85E+00	2.28	↑
P90	(+)-Nootkatone	1.12	6.63E-03	1.60E-02	-5.97	↓
P91	N-(3-hydroxypropyl) arachidonamide	1.12	5.97E-03	3.07E-01	-1.71	↓
P92	Isomer of Delbruine	1.11	7.50E-03	1.11E-01	-3.17	↓
P93	2-(1-azepanylmethyl)-1-vinylcyclododecanol	1.11	8.89E-03	2.61E+01	4.71	↑
P94	Germacrone	1.11	1.09E-02	2.75E+00	1.46	↑
P95	3-Deoxyaconine	1.11	1.05E-02	2.33E+02	7.86	↑
P96	Deltaline	1.10	1.12E-02	1.27E-01	-2.98	↓
P97	α-Curcumene	1.10	1.09E-02	2.16E+00	1.11	↑
P98	8-linoleoylbenzoyl-3,13-deoxyaconine	1.09	9.81E-03	1.29E+01	3.69	↑
P99	Isomer of talatizamine	1.09	1.54E-02	6.37E+00	2.67	↑
P100	8-linoleoylbenzoylaconine	1.07	1.32E-02	8.13E+00	3.02	↑
P101	Zederone	1.06	1.36E-02	2.13E-01	-2.23	↓
P102	(+)-agasizine	1.06	1.28E-02	1.92E+01	4.26	↑
P103	Condelphine	1.05	2.02E-02	3.34E+01	5.06	↑
P104	Kusnezosine C	1.04	1.87E-02	4.47E+00	2.16	↑
P105	Aconine	1.04	2.15E-02	2.51E-01	-2.00	↓
P106	11-Acetylhetisine	1.03	1.86E-02	9.46E+00	3.24	↑
P107	8-O-acetyl-15-hydroxyneoline	1.03	2.06E-02	8.85E+00	3.15	↑

Table S6. MRM parameters of 9 compounds and 2 ISs

Identification	Q1 Mass	Q3 Mass	DP (V)	EP (V)	CE (V)	CXP (V)
hypaconitine	616.400	556.200	126.000	10.000	45.000	16.000
Berberine (IS_1)	336.100	292.200	125.000	12.000	37.000	13.000
aconitine	646.400	586.400	140.000	12.000	47.000	10.000
mesaconitine	632.200	572.300	119.000	10.000	41.000	23.000
benzoylhypaconine	574.100	542.300	122.000	10.000	47.000	13.000
benzoylmesaconine	590.200	105.100	135.000	9.000	90.000	10.000
aconine	500.200	58.200	120.000	10.000	86.000	16.000
hypaconine	470.200	438.200	105.000	10.000	53.000	12.000
Miltefosine (IS_2)	408.300	124.900	119.000	10.000	42.000	9.000
LPC (18:0)	524.400	104.000	136.000	10.000	31.000	10.000
LPC (18:1)	522.200	184.100	139.000	10.000	36.000	12.000

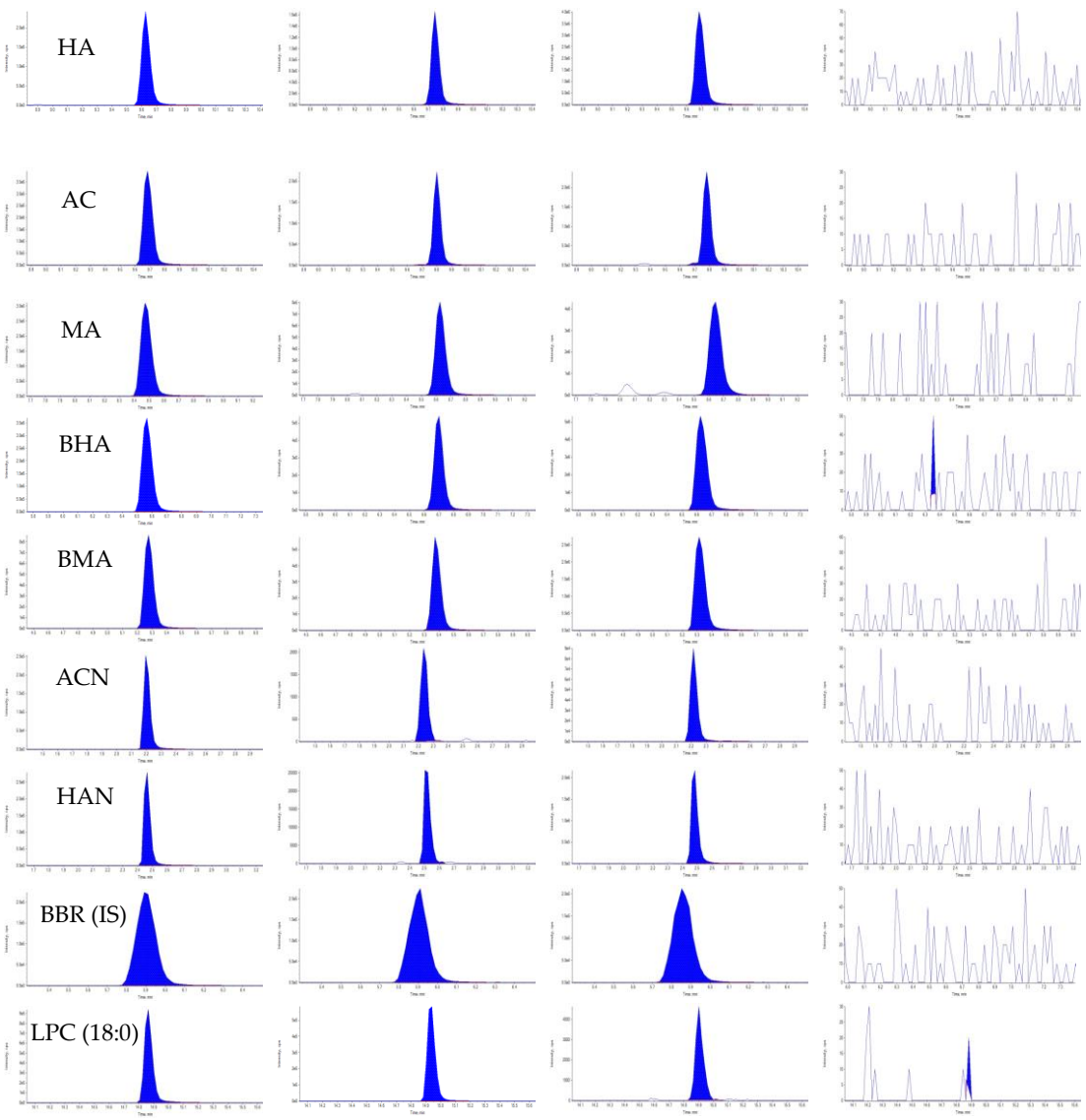

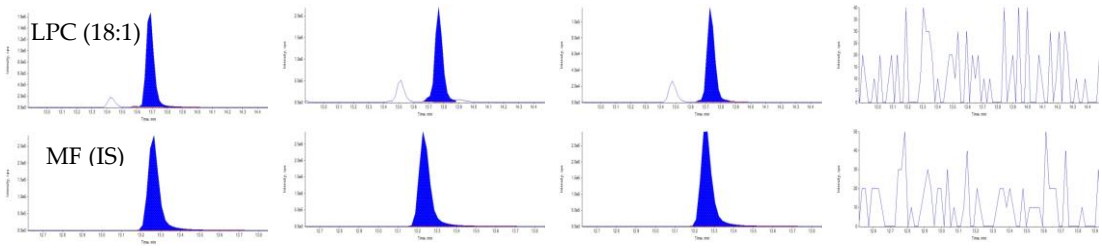

Figure S2. Typical mass spectra of 9 compounds and 2 ISs in standard substances. (**a**) Standard sample; (**b**) unfermented Yaomu samples; (**c**) fermented Yaomu samples; (**d**) blank matrices.

Table S7. The calibration curves, linearity range, and LOQs, LODs of 9 compounds (n=6)

Compounds	Regression equation	R^2	Linearity range (ng mL^{-1})	LOQ (ng mL^{-1})	LOD (ng mL^{-1})
aconitine	Y=0.00375x+0.00141	0.9928	1-500	0.05	0.015
hypaconitine	Y=0.00297x+0.0022	0.9941	1-500	0.05	0.015
mesaconitine	Y=0.00427x+0.00211	0.9952	1-500	0.1	0.03
benzoylhypaconine	Y=0.00481x+0.00481	0.9935	1-500	0.05	0.015
benzoylmesaconine	Y=0.00101x+0.0152	0.9983	50-750	0.1	0.03
aconine	Y=0.000243x+0.0000421	0.9931	1-500	0.05	0.015
hypaconine	Y=0.00247x+0.00162	0.9920	1-500	0.05	0.015
LPC (18:0)	Y=0.00137x-0.0041	0.9980	50-750	0.1	0.03
LPC (18:1)	Y=0.0019x+0.00342	0.9924	1-500	0.05	0.015

Table S8. Precision, accuracy, and stability for 9 compounds.

**Compounds**	**Spiked concentration (ng/mL)**	**Intra-day (n=3)**		**Inter-day (n=6)**		**Stability (n=6)**	
		**Accuracy (%)**	**RSD (%)**	**Accuracy (%)**	**RSD (%)**	**Accuracy (%)**	**RSD (%)**
aconitine	500	97.3	1.48	97.7	2.72	95.4	2.85
	50	96.0	0.90	95.1	2.10	100.1	1.21
	5	99.9	5.92	96.9	1.03	103.7	3.90
hypaconitine	500	95.6	0.92	98.4	1.61	95.2	2.17
	50	99.1	1.64	97.4	1.61	100.8	1.38
	5	97.4	6.73	98.3	6.08	103.5	1.81
mesaconitine	500	96.4	1.49	96.8	2.27	96.9	2.74
	50	97.5	2.27	100.9	0.98	98.9	2.14
	5	100.9	1.09	97.1	1.57	102.67	2.28
benzoylhypaconine	500	96.7	2.96	97.0	2.80	98.7	2.67
	50	96.2	1.00	95.0	2.08	100.1	2.19
	5	100.2	7.45	95.6	1.42	104.0	2.02
benzoylmesaconi	500	96.4	2.41	97.3	1.60	94.2	2.73

ne	250	99.9	1.72	102.7	2.70	101.4	1.08
	50	96.2	0.24	95.5	2.14	96.0	3.07
	500	96.4	1.29	95.2	2.74	96.2	3.05
aconine	50	97.5	1.33	96.2	2.36	103.1	3.94
	5	105.3	3.33	94.00	0.90	104	3.60
	500	96.0	0.54	97.6	2.63	94.7	3.75
hypaconine	50	96.2	1.62	95.6	3.41	103.1	3.94
	5	103.6	6.62	96.0	1.73	96.2	7.42
	500	98.9	2.46	102.1	2.80	98.1	1.35
LPC (18:0)	250	100.4	2.72	97.0	1.34	100.7	3.66
	50	100.7	3.85	103.7	2.08	97.4	3.56
	500	96.3	1.00	99.5	1.23	97.2	1.46
LPC (18:1)	50	97.4	1.17	96.0	2.09	95.5	1.93
	5	97.7	0.95	96.9	2.78	95.6	2.31

Table S9. Repeatability for 9 compounds (n=6)

Compounds	Test concentration (ng/mL)	RSD (%)
aconitine	66.27	1.72
hypaconitine	134.00	0.82
mesaconitine	154.50	1.99
benzoylhypaconine	78.10	1.88
benzoylmesaconine	209.00	2.26
aconine	14.30	1.59
hypaconine	9.31	2.68
LPC (18:0)	302.67	0.68
LPC (18:1)	97.58	2.05

Table S10. Extraction recovery for 9 compounds (n=3)

Compounds	Original concentration (ng/mL)	Spiked concentration (ng/mL)	Measured concentration (ng/mL)	Average recovery (%)	RSD (%)
aconitine	67.30	53.00	117.00	95.85	1.43
		68.00	139.00	105.44	3.42
		80.00	145.60	99.25	1.04
hypaconitine	134.00	107.00	238.83	97.95	1.30
		134.00	255.00	90.30	3.70
		160.00	278.00	90.00	7.05
mesaconitine	154.50	123.00	270.17	94.04	2.30
		154.50	308.00	99.35	1.30
		185.00	330.80	95.30	3.17
benzoylhypaconine	78.10	61.00	137.33	97.05	4.34
		78.00	157.80	102.31	1.04
		93.00	167.50	96.13	0.82

		167.00	366.30	94.21	3.34
benzoylmesaconine	209.00	209.00	416.60	99.33	1.62
		250.00	459.40	100.16	4.87
		11.00	25.80	102.68	5.61
aconine	14.30	14.00	27.50	94.29	1.42
		17.00	31.70	102.35	1.13
		7.00	16.30	104.29	2.36
hypaconine	9.00	9.00	18.37	104.11	1.41
		11.00	19.30	93.64	0.74
		242.00	546.75	100.87	3.01
LPC (18:0)	302.60	303.00	595.50	96.67	2.43
		363.00	661.20	98.79	2.44
		78.00	173.00	96.80	7.77
LPC (18:1)	97.50	97.00	185.70	90.93	1.73
		117.00	221.00	105.56	0.85

Table S11. Content of 9 components in the YM before and after fermentation (n=6, mean ± SD)

	batch number	Aconitine (μg/g)	hypaconitine (μg/g)	mesaconitine (μg/g)	benzoylhypaconine (μg/g)	benzoylmesaconine (μg/g)	aconine (μg/g)	hypaconine (μg/g)	LPC (18:0) (μg/g)	LPC (18:1) (μg/g)
Fermented	YM20220301	1.38±0.44	41.25±1.67	2.88±1.13	27.96±3.78	91.04±3.39	13.41±2.62	31.74±2.86	0.69±1.00	3.03±6.22
	YM20230510	0.75±0.09	24.77±2.34	1.01±0.25	27.58±4.18	60.37±7.78	8.74±1.70	19.98±2.42	0.18±3.69	1.59±0.23
	YM20220601	2.84±0.94	41.64±7.18	6.39±1.63	27.36±7.58	93.30±6.77	3.65±1.39	5.76±1.13	0.14±1.80	2.46±0.30
	YM20230328	0.53±0.05	18.95±1.21	1.59±0.23	40.23±2.56	100.81±5.56	9.18±1.14	27.99±1.51	0.07±0.94	0.41±0.15
	YM-PZ0050	0.72±0.04	31.43±1.17	1.27±0.17	25.32±1.33	79.58±5.99	12.17±2.21	28.40±2.10	0.04±1.10	0.92±0.79
	YM20240407	1.27±0.16	36.72±4.80	2.71±0.31	34.95±2.07	93.54±11.83	11.92±1.96	28.89±4.23	0.13±1.32	1.00±0.86
Unfermented	WFJ-S1	10.69±0.74	99.57±5.16	84.89±6.03	10.40±1.05	121.32±4.13	1.93±0.34	3.04±0.10	41.72±1.46	594.67±8.02
	WFJ-S2	14.67±0.45	80.14±2.37	42.95±2.59	14.67±0.98	146.12±4.63	2.79±0.23	2.31±1.05	39.96±7.27	170.36±7.27
	WFJ-S3	14.67±0.20	177.35±4.12	54.37±1.03	25.19±0.82	110.50±3.35	1.52±0.30	3.17±0.75	35.87±1.31	192.93±10.19
	WFJ-S4	8.79±1.14	71.11±1.10	20.50±3.08	10.09±1.43	110.92±4.73	2.09±1.13	1.34±0.80	40.16±2.07	258.94±2.00
	WFJ-S5	19.13±0.50	142.84±3.67	67.26±1.76	22.10±0.67	179.27±4.40	3.40±0.26	3.51±0.20	58.40±1.60	372.71±9.53
	WFJ-S6	15.36±1.45	171.60±8.84	57.76±4.28	26.35±2.49	108.70±9.12	1.58±0.23	3.00±0.32	37.12±1.75	142.98±5.21
